# Development of a multi faceted platform containing a tetrazine, fluorophore and chelator: synthesis, characterization, radiolabeling, and immuno-SPECT imaging

**DOI:** 10.1186/s41181-022-00164-1

**Published:** 2022-06-06

**Authors:** Anthony W. McDonagh, Brooke L. McNeil, Julie Rousseau, Ryan J. Roberts, Helen Merkens, Hua Yang, François Bénard, Caterina F. Ramogida

**Affiliations:** 1 Department of Chemistry, Simon Fraser University, Burnaby, BC V5A 1S6 Canada; 2 Life Sciences Division, TRIUMF, Vancouver, BC V6T 2A3 Canada; 3 Department of Molecular Oncology, BC Cancer, Vancouver, BC V5Z 1L3 Canada

**Keywords:** Probes, Indium-111, Actinium-225, BODIPY, Tetrazine, DOTA, Multi-modal, Fluorophore

## Abstract

**Background:**

Combining optical (fluorescence) imaging with nuclear imaging has the potential to offer a powerful tool in personal health care, where nuclear imaging offers in vivo functional whole-body visualization, and the fluorescence modality may be used for image-guided tumor resection. Varying chemical strategies have been exploited to fuse both modalities into one molecular entity. When radiometals are employed in nuclear imaging, a chelator is typically inserted into the molecule to facilitate radiolabeling; the availability of the chelator further expands the potential use of these platforms for targeted radionuclide therapy if a therapeutic radiometal is employed. Herein, a novel mixed modality scaffold which contains a tetrazine (Tz)––for biomolecule conjugation, fluorophore—for optical imaging, and chelator—for radiometal incorporation, in one construct is presented. The novel platform was characterized for its fluorescence properties, radiolabeled with single-photon emission computed tomography (SPECT) isotope indium-111 (^111^In^3+^) and therapeutic alpha emitter actinium-225 (^225^Ac^3+^). Both radiolabels were conjugated in vitro to trans-cyclooctene (TCO)-modified trastuzumab; biodistribution and immuno-SPECT imaging of the former conjugate was assessed.

**Results:**

Key to the success of the platform synthesis was incorporation of a 4,4′-dicyano-BODIPY fluorophore. The route gives access to an advanced intermediate where final chelator-incorporated compounds can be easily accessed in one step prior to radiolabeling or biomolecule conjugation. The DOTA (1,4,7,10-tetraazacyclododecane-1,4,7,10-tetraacetic acid) conjugate was prepared, displayed good fluorescence properties, and was successfully radiolabeled with ^111^In & ^225^Ac in high radiochemical yield. Both complexes were then separately conjugated in vitro to TCO modified trastuzumab through an inverse electron demand Diels–Alder (IEDDA) reaction with the Tz. Pilot small animal in vivo immuno-SPECT imaging with [^111^In]In-DO3A-BODIPY-Tz-TCO-trastuzumab was also conducted and exhibited high tumor uptake (21.2 ± 5.6%ID/g 6 days post-injection) with low uptake in non-target tissues.

**Conclusions:**

The novel platform shows promise as a multi-modal probe for theranostic applications. In particular, access to an advanced synthetic intermediate where tailored chelators can be incorporated in the last step of synthesis expands the potential use of the scaffold to other radiometals. Future studies including validation of ex vivo fluorescence imaging and exploiting the pre-targeting approach available through the IEDDA reaction are warranted.

**Supplementary Information:**

The online version contains supplementary material available at 10.1186/s41181-022-00164-1.

## Background

Radiometals, due to their distinct physical properties, are popular in medicine for the diagnosis and treatment of diseases. Several gamma (γ)- or positron- (β^+^) emitting radiometals have been identified for single photon emission computed tomography (SPECT) or positron-emission tomography (PET) imaging, respectively (Kostelnik and Orvig 2019; Blower 2015). For example, SPECT isotope indium-111 (^111^In, t_1/2_ = 2.8 d, EC (100%), I_γ_ 94.1% (245.4 keV), 90.6% (171.3 keV)) and PET isotope zirconium-89 (^89^Zr, t_1/2_ = 78.4 h, β^+^ (23%)) are popular isotopes for nuclear imaging of radiopharmaceuticals with long biological half-lives, such as monoclonal antibodies (mAbs). Therapeutic radiometals, on the other hand, emit cytotoxic radiation in the form of alpha (α) particles, beta (β^−^) particles, or Meitner-Auger electrons (MAEs) and within the last five years, actinium-225 (^225^Ac, t_1/2_ 9.9 d), a radiometal with four net α and two β^−^ decays, has emerged as a popular isotope for targeted alpha therapy (TAT) (Thierer and Tomson 2017; Morgenstern et al. 2018). Although in its early stages, promising ^225^Ac-labeled drug candidates have been generated, with some in clinical trials (Morgenstern et al. 2020).

In molecular imaging, combining fluorescence imaging with diagnostic radionuclides has sparked interest over the last decade (Bernhard et al. 2010, 2012; Maindron et al. 2016; Lhenry et al. 2015; Ariztia et al. 2022). This has led to the development of bimodal imaging probes, where a BODIPY fluorophore and either a SPECT (^111^In) or PET (^89^Zr) emitter complement one another. Their complementarity and extreme sensitivity of the techniques at very low concentrations is advantageous for preclinical and clinical use. For example, in intraoperative imaging guided surgery, PET or SPECT can locate tumors in a patient in the preoperative staging process while fluorescence imaging can improve therapeutic outcome by assisting surgeons to efficiently locate and remove them during surgery (Leeuwen et al. 2020). Using this technology, hybrid probes have become apparent in preclinical settings to identify and remove prostate-specific-membrane antigen (PSMA) and human epidermal growth factor receptor-2 (HER2) positive tumors in animal models (Hensbergen et al. 2020; Deken et al. 2019). There are, however, limited examples using this technology with theranostic isotopes (combination of diag*nostic* and *thera*peutic radionuclides).

There are several bioconjugate handles that have been exploited in radiopharmaceutical design in order to attach a probe to a targeting vector (e.g., antibody), for example isothiocyanates or succinimides are popular choices which react with available primary amines on the biomolecule. Alternatively, the inverse electron demand Diels–Alder (IEDDA) reaction between a 1,2,4,5-tetrazine (Tz) and *trans*-cyclooctene (TCO) has been utilized, where the TCO is attached to the antibody and the tetrazine is part of the radiopharmaceutical (Altai et al. 2017; Patra et al. 2016; Oliveira et al. 2017). The extreme selectivity, rapid kinetics, and biorthogonal nature of this “click” reaction allows both in vivo targeting and pre-targeting, where the latter allows injection of the slowly accumulating antibody-TCO first, prior to the injection of a rapidly clearing Tz-containing radioligand which, once administered, ligates to the antibody in vivo*.* With this protocol, circulation time of radioactivity and uptake of radioisotopes in healthy tissue (and thus radiation burden to non-target tissue) is significantly reduced. Radionuclides with short half-lives can also be facilitated.

To the best of our knowledge there are only two examples in the literature of probes bearing a tetrazine, chelator and a fluorophore. In 2016, Weissleder and co-workers disclosed the first example starting form *p*-cyanophenyl-BODIPY where they formed the tetrazine and subsequently introduced deferoxamine (DFO), an acyclic chelator used to coordinate ^89^Zr, on the BODIPY boron atom (Meimetis et al. 2016). The molecule was conjugated with trastuzumab-TCO, radiolabelled and its potential as a PET/fluorescence mAb imaging probe was investigated. In a more recent example, the Goncalves group explored commercially available dichlorotetrazine as a modular platform to conveniently introduce imaging probes via the S_N_Ar reaction (Canovas et al. 2019). Despite low to moderate yields, a series of scaffolds were developed with a DOTA macrocycle and either BODIPY, cyanate, and rhodamine fluorophores. A DOTAGA-cyanine-tetrazine probe was selected for further study and, in a similar fashion, was clicked to a functionalized trastuzumab, radiolabeled with ^111^In, and investigated as an imaging probe. Unfortunately, due to the presence of electronically enriching heteroatoms reducing the reactivity of the tetrazine, the IEDDA reaction was observed to be slow and took 16 h to achieve optimal conversion. Therefore, since tetrazine-TCO conjugations are completed within minutes, these scaffolds would be unsuitable for in vivo pretargeting.

There are opportunities for improving these scaffolds regarding an ideal synthetic route (in terms of yield and reproducibility) and application for diagnosis and therapy through in vivo pretargeting. To address this, we took a previously reported orthogonal protected dipeptide (Thiele et al. 2017) as a platform to synthesize a multi-modal scaffold with a BODIPY fluorophore, chelator and tetrazine. Key to its success was a 4,4′-dicyano-BODIPY. The synthesis is simple, convenient, high yielding and gives access to an advanced tetrazine-BODIPY-amine intermediate where, chelators specific to certain radiometals can be easily introduced in one step. Disclosed herein, is its synthesis, characterization, ^111^In and ^225^Ac radiolabeling, along with their proof-of-concept bioconjugation to TCO-modified trastuzumab (Herceptin, anti-HER2). Furthermore, pilot small animal in vivo immuno-SPECT imaging studies employing the pre-clicked ^111^In-labeled scaffold were also conducted to validate the targeted approach.

## Results

### Synthesis and characterization

**Scheme 1:**
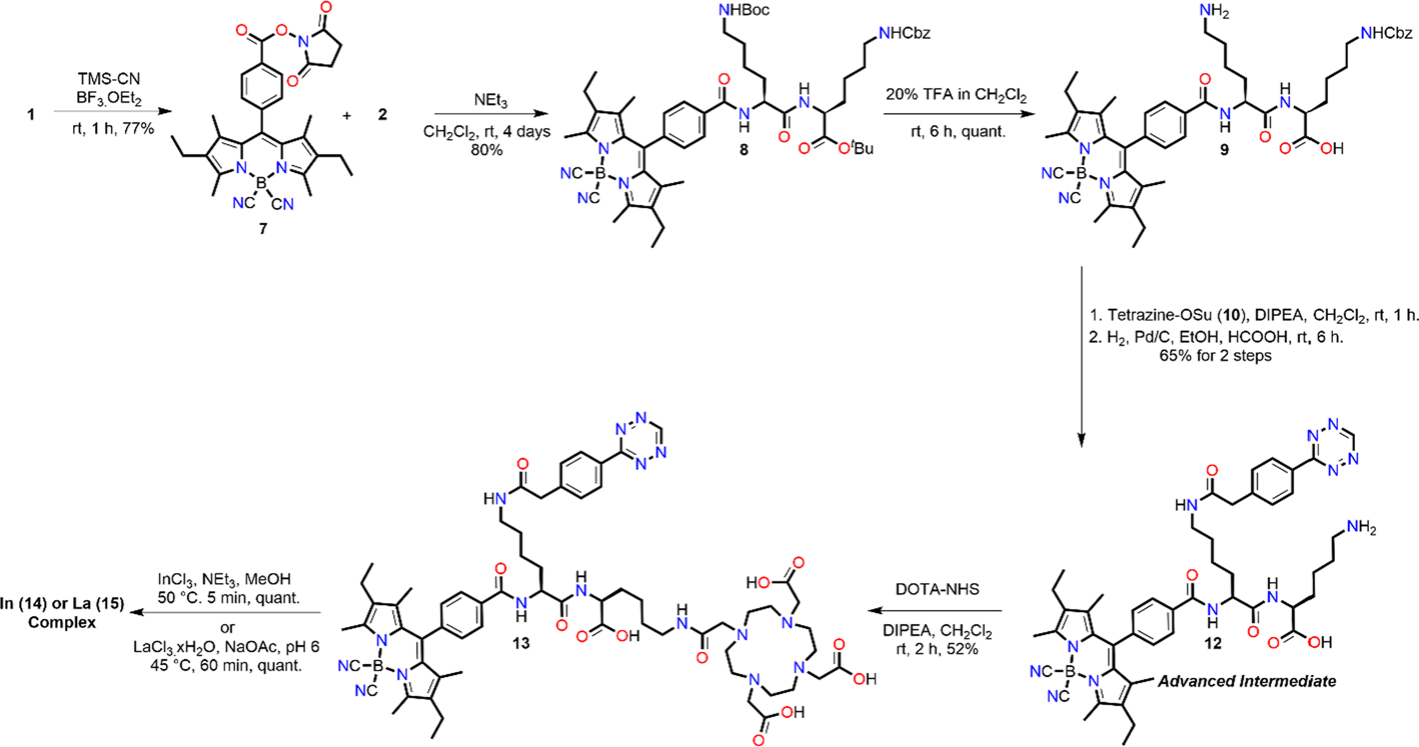
Synthesis of DO3A-BODIPY-Tz (**13**) via advanced intermediate **12**

Our synthetic route began with converting F-BODIPY succinimide ester **1** to the cyano derivative **7** (Scheme 1). This was easily accessible with BF_3_·OEt_2_ and TMS-CN to give **7** in a 77% yield after 1 h (Zhao et al. 2016). **7** was then reacted with secondary amine **2** to give **8** in 80% yield. Next the Boc and *tert*-butyl ester were deprotected with 20% TFA in CH_2_Cl_2_ over 6 h. The reaction was found to proceed smoothly giving **9** in quantitative yield. The crude amine was then reacted with tetrazine **10** to give **11** in 90% yield. Next the CBz was hydrogenated for 6 h to give **12** in 72% yield. Interestingly, the tetrazine was also reduced but spontaneously re-oxidized on exposure to air (Seibold et al. 2017). It is worth noting that prolonged reaction times, > 6 h, will result in decomposition. Finally, the chelator DOTA-NHS was reacted with **12** to give the final scaffold DO3A-BODIPY-Tz (**13**) in 52% yield.

**Fig. 1:**
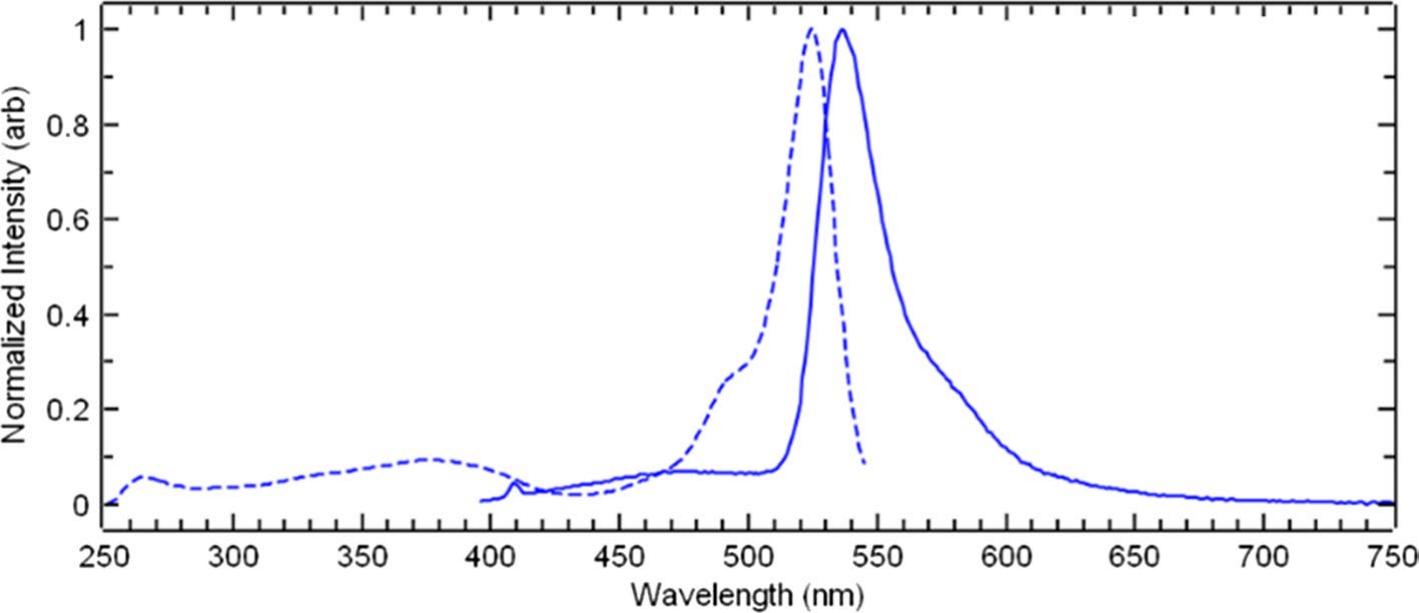
Excitation (dashed) and emission (solid) spectra for DO3A-BODIPY-Tz (**13**) in DMSO with *Φ*_*fl*_, (0.38), excitation λ_max_ = 524 nm, emission λ_max_ = 537 nm

**Scheme 2:**
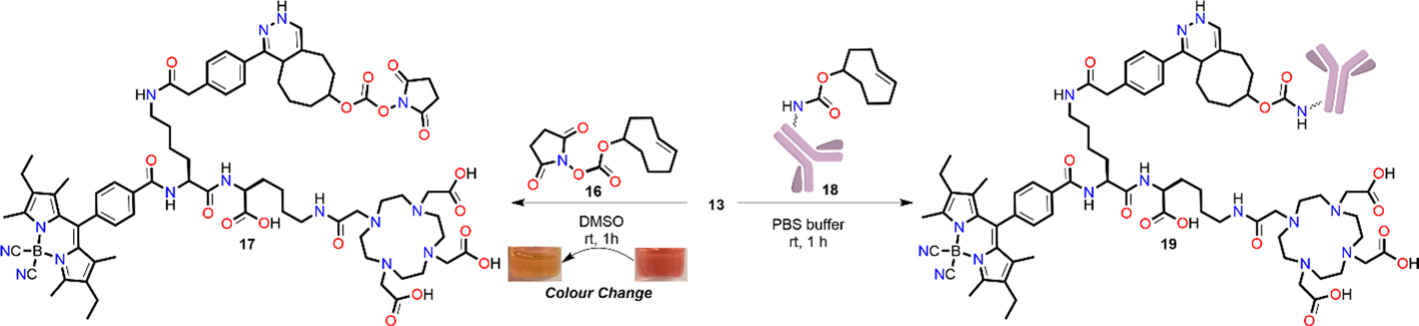
IEDDA reaction of TCO-NHS and TCO-trastuzumab with **13**

Following the synthesis of the final scaffold, the excitation and emission spectrum, and quantum yield were measured for the uncomplexed scaffold DO3A-BODIPY-Tz (**13**) in DMSO (Fig. 1), where an excitation λ_*max*_ = 524 nm, emission λ_*max*_ = 537 nm and a fluorescence quantum yield (*Φ*_*fl*_) of 0.38 was observed. There are reports of tetrazine residues deactivating the fluorescence properties of fluorophores when they belong to the same molecular entity. However, once the tetrazine is conjugated, these properties are reinstated (Oliveira et al. 2017; Wu and Devaraj 2018). To ensure this, **13** was conjugated to TCO-NHS **16** in DMSO (Scheme 2). A distinct colour change from red to orange was observed which clearly indicated tetrazine consumption. The fluorescence properties of **17** (see Additional file1: Fig. S36) were then measured and compared to **13** and 4,4′-dicyano-BODIPY (**7**) (see Additional file1: Fig. S35; λ_*max*_ excitation = 527 nm_,_ λ_*max*_ emission = 541 nm, *Φ*_*fl*_ = 0.87)**.** As expected, the *Φ*_*fl*_ increased from 0.38 to 0.79, (λ_*max*_ excitation = 525 nm_,_ λ_*max*_ emission = 537 nm), exhibiting a comparable efficiency to **7**.

To further validate our scaffold prior to radiolabelling, non-radioactive In^3+^ and La^3+^ (an ^225^Ac^3+^ surrogate) complexes were prepared in quantitative yields from scaffold **13** (Scheme 1). The compounds were found to precipitate during the reaction and could be isolated by centrifugation. In addition, the IEDDA reaction was performed with **13** and TCO-modified trastuzumab (**18**; Scheme 2, with 4.3 TCO’s per antibody as determined by MALDI-TOF MS/MS) (Membreno et al. 2019). The click reaction proceeded smoothly and after purification, the bioconjugate was confirmed using absorption spectroscopy (to measure protein concentration), fluorescence and MALDI-TOF MS/MS; the average number of DO3A-BODIPY moieties per antibody was determined to be 2.9.

### Radiolabeling

**Fig. 2:**
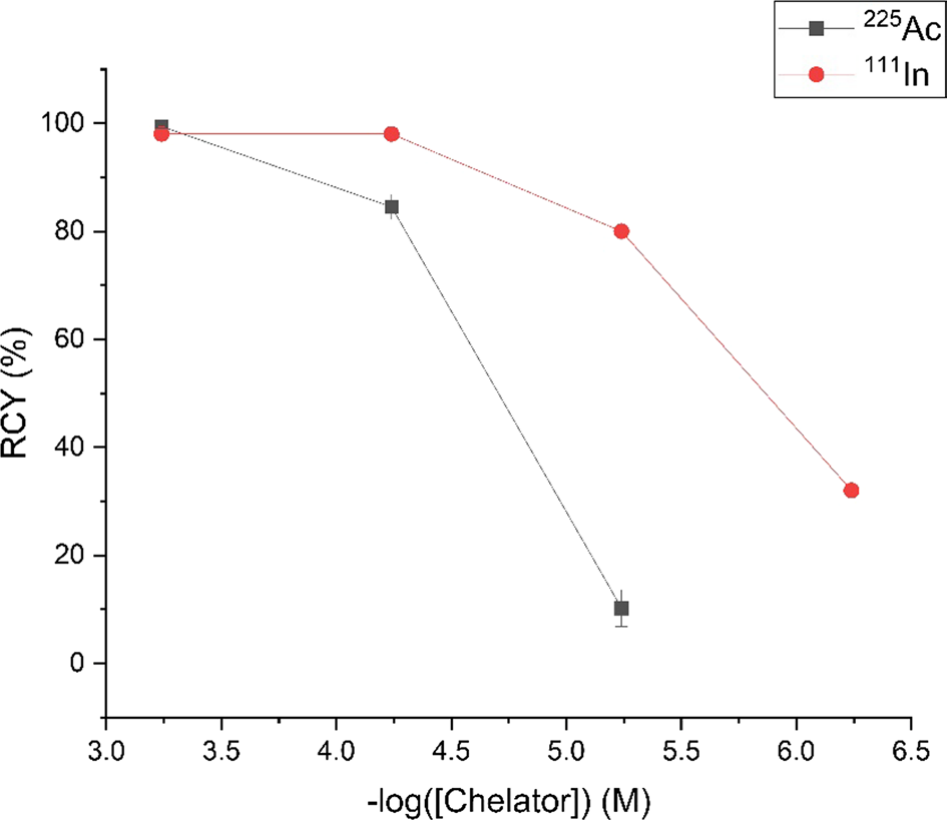
Radiochemical yields (RCYs, %) for ^225^Ac^3+^ (80 °C, 60 min, 0.15 M ammonium acetate, pH 7) and ^111^In^3+^ (45 °C, 60 min, 0.1 M sodium acetate, pH 5) radiolabeling reactions of DO3A-BODIPY-Tz (**13**) at ligand concentrations 5.8 × 10^–4^ to 10^–7^ M

Radiolabeling was performed with diagnostic ^111^In and therapeutic ^225^Ac. Varying amounts of DO3A-BODIPY-Tz (**13**) (100, 10, 1, and 0.1 µg) were radiolabeled with [^111^In]InCl_3_ (3.7 MBq) in sodium acetate (0.1 M, pH 5) with final chelator concentrations of 5.8 × 10^–4^ to 5.8 × 10^–7^ M. Each reaction was carried out at 45 °C and monitored by radio-TLC after 30 min and 60 min. Resulting radiochemical yields (RCYs) were determined to be 97, 94, 78, and 20% at 30 min, and 98, 98, 80, and 32% (*n* = 1) at 60 min for 100, 10, 1, and 0.1 µg of **13**, respectively (Fig. 2). Following C_18_ Sep-pak purification of the radiolabeled scaffold, the IEDDA reaction with [^111^In]In-DO3A-BODIPY-Tz and TCO-trastuzumab was then carried out. To ensure a sufficiently high specific activity of the final bioconjugate for preclinical in vivo studies (*vide infra*), 9.25 µg of the ligand was incubated with a high activity of [^111^In]InCl_3_ (272 MBq), and quantitative radiochemical conversion (RCY > 99%) was confirmed after 45 min at 45 °C. The reaction mixture was cooled and added to the previously prepared TCO modified antibody (TCO-trastuzumab; 2:1 radioligand-to-antibody ratio) in PBS buffer. After allowing to react at room temperature for 1 h (60% radiolabeling yield) the radioimmunoconjugate was purified by PD-10 size-exclusion chromatography and radiochemical purity (RCP) was determined to be > 99% by radioTLC, resulting in a final specific activity of 0.3 MBq/μg.

Similarly, scaffold **13** was incubated at concentrations of 5.8 × 10^–4^ to 5.8 × 10^–6^ M with 80 kBq of ^225^Ac in 0.15 M ammonium acetate (0.15 M, pH 7). The 100 µL reactions required heating to 80 °C to facilitate Ac^3+^ complexation with DOTA and were monitored by radio-TLC after 30 min and 60 min. RCYs were determined to be 99 ± 0.3, 82 ± 3 and 10 ± 5% for 30 min and 99 ± 0.3, 85 ± 2 and 10 ± 3% for 60 min for ligand concentrations of 5.8 × 10^–4^ to 5.8 × 10^–6^ M (*n* = 3), respectively.

In vitro human serum stability of [^225^Ac]Ac- and [^111^In]In-DO3A-BODIPY-Tz was also conducted over 5 or 6 days (Table 1), where an equal volume of human serum was added to the pre-formed radiometal complexes and incubated at 37 °C. After 4 h, the [^225^Ac]Ac-DO3A-BODIPY-Tz complex remained stable (98 ± 1%), with a moderate decrease to 84 ± 0.6% after 1 day and remained 54 ± 1% intact after 5 days. Similarly, [^111^In]In-DO3A-BODIPY-Tz remained 87 ± 4, 70 ± 2, and 50 ± 1% intact after 1, 3, and 6 days, respectively.

**Table 1:** Stability of ^225^Ac- and ^111^In-labeled DO3A-BODIPY-Tz (**13**) complexes in human serum at 37 °C (*n* = *3*)

	Time point (d)					
% intact	0.2	1	3	4	5	6
^111^In-**13**	ND^*a*^	87 ± 4	70 ± 2	ND^*a*^	ND^*a*^	50 ± 1
^225^Ac-**13**	98 ± 1	84 ± 1	69 ± 2	61 ± 5	54 ± 1	ND^*a*^

^*a*^ND = not determined

### Biodistribution and SPECT-CT imaging studies

**Fig. 3:**
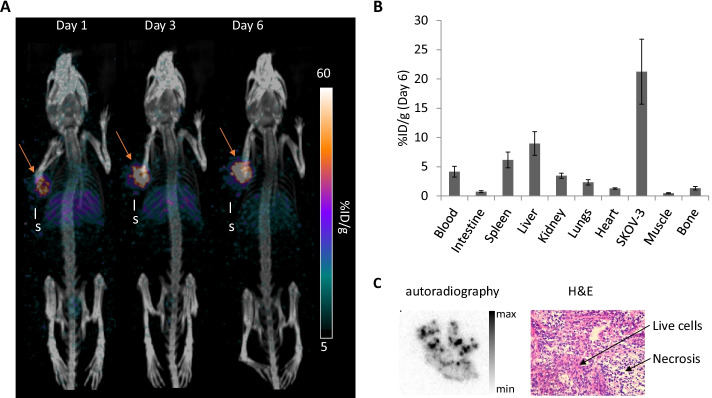
In vivo SPECT/CT imaging and biodistribution of [^111^In]In-DO3A-BODIPY-Tz-TCO-trastuzumab in SKOV-3 (HER2 +) tumors bearing nude mice. (A) SPECT/CT images in %ID/g are shown for 1, 3 and 6 days post-injection (26.7 µg, 4.2 MBq). The tumor is shown by the orange arrow, l: liver and s: spleen. (B) Biodistribution data are presented as mean ± SD of %ID/g for the main organs of interest and the SKOV-3 tumor (26.3 ± 0.3 μg, 4.1 ± 0.1 MBq, n = 4). (C) At 6 days post-injection, half of the tumor was frozen and sectioned for autoradiography. A representative image is shown for the ^111^In signal (left panel) and the consecutive H&E stained section (right panel)

The “pre-clicked” [^111^In]In-DO3A-BODIPY-Tz-TCO-trastuzumab was prepared as described above and was injected in SKOV-3 (human HER2-positive) tumor bearing mice. SPECT/CT images show high tumor uptake with high tumor-to-background ratios as early as 1-day post-injection, in addition to expected uptake in the spleen and the liver because of antibody metabolism and excretion (Fig. 3a). From Day 1 to Day 6 post-injection, an increase of both the tumor uptake and the contrast were observed, which is characteristic of radioimmunoconjugates. These results were confirmed by the biodistribution study performed 6 days post-injection of the [^111^In]In-DO3A-BODIPY-Tz-TCO-trastuzumab with a high tumor uptake of 21.2 ± 5.6%ID/g, demonstrating remaining binding capacity of the bioconjugate (Fig. 3b and Additional file1: Table S1). For the rest of the body, uptake in the spleen (4.2 ± 0.9%ID/g), the liver (9.0 ± 2.0%ID/g), kidneys (3.5 ± 0.4%ID/g), lungs (2.3 ± 0.5%ID/g) and the heart (1.3 ± 0.1%ID/g), all well-circulating organs, were observed. Other organs such as the muscle or the intestines remained below 1%ID/g. Finally, autoradiography was performed and shows a heterogeneous but efficient deep penetration into the tumor tissue (Fig. 3c). Expected presence of live tumor cells and necrotic areas, which are expected in SKOV-3 tumors was confirmed by histology.

## Discussion

Initially our scaffold design comprised of the conventional 4,4′-difluoro-BODIPY fluorophore (Additional file1: Scheme S1). Despite numerous attempts and alternative strategies for completing the synthesis of the platform using the F-BODIPY fluorophore, the route was unsuccessful resulting in either BF_2_ core removal or complete decomposition (see Additional File), despite there being reports in the literature of similar transformations being successful in the presence of F-BODIPY dyes (Alnajjar et al. 2018; Tsuji et al. 2018; Sui et al. 2016; Compton et al. 2019; Ieda et al. 2014). This documented instability of the BF_2_ core (Smithen et al. 2012; Crawford and Thompson 2010; Rumyantsev et al. 2013; Yu et al. 2015; Urieta et al. 2015; Liras et al. 2016; Lundrigan et al. 2014; Summers et al. 2017) was overcome by replacing it with the recently reported 4,4′-dicyano-BODIPY identified by Vicente and Bobadova-Parvanov as the most stable derivative under acidic conditions (Nguyen et al. 2016; Wang et al. 2018). The increased stability is believed to be a result of enhanced aromaticity, decreased charge density on the boron, and the formation of a stable 4,4′-dicyano-BODIPY-TFA complex. To the best of our knowledge there has only been one example of an investigation incorporating 4,4′-dicyano-BODIPYs into a chemical synthesis with protecting groups (Uriel et al. 2020). To reassure the stability of the cyano BODIPY core in acidic conditions, **7** was treated with TFA in CDCl_3_ and monitored via ^1^H NMR (see Additional file1: Fig. S11) (Wang et al. 2018). Unlike the BF_2_ counterpart, there was no indication of instability even after 11 days. An alternative route was initially investigated, where the Cbz on **9** underwent hydrogenolysis followed by insertion of the chelator, deprotection, then insertion of the tetrazine. However, this combination was found to be insufficient due to decomposition, poor yields, and lack of reproducibility. Some intermediates were also amphipathic, making them difficult to purify and characterise. It was rationalised that the complexity of the chelator was contributing to these observations. Gratifyingly in the presented route, no side reactions or decomposition were observed, suggesting the chelator was in fact interfering with the reaction outcome.

The key intermediate **12** is highly versatile; with the fluorophore, tetrazine and primary amine functionalities intact, chelators can be easily incorporated in one step. This is highly advantageous, and unlike other probes which require additional steps after introducing the ligand, **12** can easily give access to a library of multi-modal probes specific for therapeutic or diagnostic radiometals by conjugating the appropriate chelator. For example, DOTA, a chelator that coordinates a variety of radiometals was used in this study, and DO3A-BODIPY-Tz (**13**) was used in subsequent characterization and radiolabeling studies.^111^In and ^225^Ac radiolabeling of DO3A-BODIPY-Tz (**13**) was successful using conventional conditions and with similar efficiencies compared to other DOTA-constructs (Ramogida et al. 2019; Price et al. 2013). The in vitro stability in human serum of the labeled platform was assessed, and appeared to be moderately stable with 50 ± 1% (at 6 days) and 54 ± 1% (at 5 days) intact for the ^111^In- and ^225^Ac-labeled platform, respectively. These results may be sufficient for further in vivo pre-targeting studies since Tz-based radioligands can accumulate at the tumor within 4 h (Keinänen et al. 2019; Poty et al. 2019). Future improvement to stability of the radiometal-chelate complexes can be envisioned by exchanging the DO3A chelator to a tailored ligand for either ^111^In and ^225^Ac. The modularity of the synthetic approach would allow facile chelator exchange starting from advanced intermediate **12**.

To study the efficiency of the “click” reaction between the Tz-containing platform and TCO-conjugated antibody, the in vitro IEDDA “click” reaction of [^225^Ac]Ac-DO3A-BODIPY-Tz with TCO-trastuzumab was carried out at 37 °C for 1 h at varying ligand to antibody ratios (2:1, 4:1, and 10:1), resulting in mAb radiolabeling yields of 68, 49, and 30% (*n* = 1), respectively. Taking into account the TCO/trastuzumab ratio (4.3 as determined by MALDI-TOF MS/MS), the efficiency of the “click” ligation with [^225^Ac]Ac-DO3A-BODIPY-Tz was approximately 31, 45, and 68% for ligand to mAb ratios of 2:1, 4:1 and 10:1, respectively. While the ligation was not quantitative as seen in previous studies conjugating [^225^Ac]Ac-DOTA-tetrazine pegylated (PEG) derivatives with TCO-modified antibodies (Poty et al. 2018), it is reasonable to believe the resulting radiolabeled bioconjugates would be sufficient for targeted preclinical in vivo studies. Specifically, targeted in vivo small animal biodistribution and imaging was possible for the ^111^In-labeled platform. Integration of a linear PEG linker between the platform and tetrazine could aid in reducing any steric strain between the Tz and the TCO on the antibody, and may be a viable option for future derivations of our multi faceted platform in case that the kinetics of the IEDDA reaction are not sufficient to enable in vivo “pre-targeting”.

To evaluate the biodistribution and the pharmacokinetics of the novel immunoconjugate, the pre-“clicked” [^111^In]In-DO3A-BODIPY-Tz-TCO-trastuzumab conjugate was evaluated in vivo employing the traditional targeted approach in SKOV-3 (human HER2-positive) tumor bearing mice. High tumor uptake of the [^111^In]In-DO3A-BODIPY-Tz-TCO-trastuzumab was visualized through SPECT images and confirmed by biodistribution studies 6 days post-injection (21.2 ± 5.6%ID/g) (Fig. 3b and Additional file1: Table S1). Since it has been shown that the tumor uptake of classically labeled [^111^In]In-trastuzumab (i.e., radiometal-chelate-antibody constructs) depends on HER2 density expression, our values are slightly different but still in the expected range (McLarty et al. 2009). 4.2 ± 0.9%ID/g remained in the blood, confirming the conserved long biological half-life of the radioimmunoconjugate (Liu 2015) without degradation (Price et al. 2014). A low bone uptake of 1.3 ± 0.3%ID/g suggested low to negligible ^111^In leakage from the bioconjugate, indicating the radiometal-complex is kinetically inert in vivo over the course of the study–alleviating concerns due to the apparent moderate stability in human serum that was found in vitro. For all these non-specific organs the uptake values and general pharmacokinetics are both in accordance with previously reported data in nude mice injected with ^111^In-labeled trastuzumab with the classical labeling method (Lub-de Hooge et al. 2004; Milenic et al. 2010). Autoradiography and histology of excised tumor slices confirmed and explained the heterogeneous distribution of the radioimmunoconjugate. Equipment availability precluded ex vivo fluorescence imaging of the excised tumors; however, based on the excellent tumor uptake of the radiolabeled probe at day 6, it is reasonable to believe ex vivo fluorescence would be possible. Previous reports of radiolabeled BODIPY probes were successfully able to obtain ex vivo fluorescence images (Meimetis et al. 2016), further suggesting that these studies are feasible with our platform since both were injected at similar probe concentrations. Based on the efficiency of the Tz-TCO in vivo* “*click” reaction that has been reported in the literature (Membreno et al. 2019; Poty et al. 2019; Keinänen et al. 2020), these results suggest that the developed scaffold can be used to modify monoclonal antibodies for further development of targeting or pre-targeting strategies for both imaging and therapy. Despite their prevalence in optical imaging probes, BODIPY dyes exhibit excitation and emission bands lying in the < 600 nm range, which is suboptimal for intraoperative imaging; therefore, future scaffold designs may consider integrating dyes that excite/emit in the near infrared (NIR) I (650–900 nm) or NIR II (1000–1500 nm) windows (Ariztia et al. 2022; He et al. 2018). For example, fluorophores such as IRDye800 (Rosenthal et al. 2015; Zhou et al. 2021) or cyanine dyes (e.g., Cy5.5 (Tsai et al. 2018; Kang et al. 2016)) have been used (pre-)clinically, and will be considered when designing our next generation of multi-modal scaffolds.

## Conclusions

A novel scaffold with potential for therapeutic and diagnostic applications was synthesised from a convenient dipeptide platform. The initial scaffold design contained a 4,4′-difluoro-BODIPY, but was observed to rapidly decompose to the corresponding dipyrrin under acidic conditions. The synthesis was revised to contain the recently reported 4,4′-dicyano-BODIPY, which did not decompose when removing the Boc and *tert*-butyl ester on the dipeptide. The successful route gives access to an advanced tetrazine-BODIPY-amine intermediate where chelators can be conveniently introduced in one step. No further modifications of the scaffold are necessary and can be taken directly to radiolabeling. Additionally, the fluorescent portion of the scaffolds could be easily redesigned to include any BODIPY analogue, only if the dicyano core is present. Fluorescent analysis indicated that the tetrazine diminishes the quantum yield of the fluorophore, but its full efficiency is reinstated once the IEDDA reaction is carried out. Radiolabeling of the scaffold was successful with diagnostic ^111^In and therapeutic ^225^Ac in high radiochemical yield and represents the first mixed modality scaffold to be radiolabeled with this therapeutic radiometal. Both complexes were successfully conjugated to TCO modified trastuzumab, thereby confirming an in vitro proof of principle for these radiolabeled scaffolds. Targeted in vivo immuno-SPECT imaging and biodistribution studies of [^111^In]In-DO3A-BODIPY-Tz-TCO-trastuzumab showed high tumor uptake after 6 days, with organ uptake values comparable to conventionally ^111^In-labeled trastuzumab indicating the addition of fluorophore does not negatively affect the pharmacokinetics of the radiotracer. In vivo biodistribution and/or imaging of [^225^Ac]Ac- and [^111^In]In-labeled scaffolds as well as ex vivo fluorescence imaging employing the targeted or pre-targeted strategy are planned in the future. Radiolabeling of the scaffold with the chemically-matched theranostic pair copper-64/copper-67 (^64^Cu, t_1/2_ = 12.7 h, β^+^ (17.6%); ^67^Cu, t_1/2_ = 61.8 h, β^−^ (100%)) or widely available PET isotope gallium-68 (^68^ Ga, t_1/2_ = 67.7 m, β^+^ (88.9%)), may also be of interest for future pre-targeting studies to take advantage of the better resolution accessible by PET compared to SPECT imaging.

## Methods

### General experimental methods and instruments

All solvents and reagents, unless otherwise noted, were purchased from commercial sources and used as received without further purification. Solvents noted as ‘’dry’’ were obtained following storage over 3 Å molecular sieves. The bifunctional ligand 1,4,7,10-tetraazacyclododecane-1,4,7,10-tetraacetic acid mono-N-hydroxysuccinimide ester (DOTA-NHS-ester) was purchased from Macrocyclics (Plano, TX). Trans-cyclooctene-N-hydroxysuccinimide (TCO-NHS) was purchased from Click Chemistry Tools (Scottsdale, Az). Nuclear Magnetic Resonance (NMR) spectra were recorded using a 400, 500 & 600 MHz spectrometer. Chemical shifts are reported relative to internal Me_4_Si in CDCl_3_ (δ 0.0) or CDCl_3_ (δ 7.26), (CD_3_)_2_SO (δ 2.50), CD_3_CN (δ 1.94) HOD for D_2_O (δ 4.65) and CD_2_HOD (δ 3.31) for ^1^H and CDCl_3_ (δ 77.16), (CD_3_)_2_SO (δ 39.5), CD_3_CN (δ 118.26) and CD_3_OD (δ 49.0) for ^13^C. ^1^H-NMR signals were assigned with the aid of COSY. ^13^C signals were assigned with the aid of DEPT-135, HSQC and HMBC. Coupling constants (*J*) are reported in Hertz and are reported uncorrected. High resolution mass spectra (HRMS) were measured in positive and/or negative mode as indicated using CH_3_CN, H_2_O and/or MeOH as solvent using an Agilent LC Mass Spectrometry instrument. Infrared (IR) spectra were recorded neat on a Perkin Elmer Spectrum Two FTIR spectrometer. Only selected, characteristic absorption data are provided for each compound. Reactions were monitored by thin layer chromatography (TLC), performed on aluminium sheets pre-coated with Silica Gel 60 (HF_254_, E. Merck) and spots visualized by UV and charring with cerium (IV) molybdate solution, vanillin, permanganate, anisaldehyde or ninhydrin solutions. During reaction work-ups a TLC of each extractant was taken to ensure complete retention of the product in the organic layer. Flash column chromatography was generally employed and was carried out using silica gel 60 (0.040–0.630 mm) using a stepwise solvent polarity gradient correlated with TLC mobility. Chromatography solvents used were diethyl ether, toluene, hexane, acetonitrile, EtOAc, CH_2_Cl_2_, MeOH (Sigma Aldrich). The analysis and purification of polar compounds were carried out using an Agilent 1100 HPLC and PDA detector at 254 nm with conditions: Kinetex (10 × 150 mm, 5 µm) with a gradient of acetonitrile: 0.1% TFA from 80:20 to 50:50 over 15 min with a flow rate of 2 mL/min.

Luminescence experiments were conducted on an Edinburgh Instruments FS5 spectrometer using the SC-05 cassette. All spectra were corrected for instrument response. Excitation and emission monochromator bandwidths for **7** and **17** were set to 1 nm, and 1.5 nm for **13**. The emission spectrum for **13** was collected with the aid of a Knight Optical 395 nm long-pass filter to reduce the effects of scattering. Solution-based absolute photoluminescent quantum yields (PLQY) were obtained for **7**, **13**, and **17** via the SC-30 Integrating Sphere cassette with excitation wavelengths of 380, 490, and 378 nm respectively.

The average number of TCO moieties per antibody, or number of DO3A-BODIPY-Tz scaffolds conjugated to trastuzumab-TCO was determined by MALDI-ToF MS/MS on a Bruker autoflex speed at the Alberta Proteomics and Mass Spectrometry Facility (University of Alberta, Canada) using previously described procedures (Thiele et al. 2017; Poty et al. 2018). The [M + 2H]^2+^ mass signals from the chromatograms of purified trastuzumab and each conjugate was used to determine the average mass, and the TCO-to-protein or ligand-to-proton ratio for each was determined by subtracting the molecular weight of trastuzumab from the molecular weight of the conjugate, and then dividing by the mass of the scaffold.

### Synthesis and characterization of compounds and complexes

Compounds **2**, **20**, **21** were prepared using a modified procedure than reported (Thiele et al. 2017).

#### Fmoc-Lys(Boc)-OSu (20)

*N*-hydroxysuccinimide (245 mg, 2.13 mmol) and EDC (408 mg, 2.13 mmol) were added to a solution of Fmoc-Lys(Boc)-OH (1 g, 2.13 mmol) in dry CH_2_Cl_2_ (42 mL). After stirring the reaction mixture for 16 h under nitrogen, the solvent was removed under reduced pressure and the resulting residue was dissolved in CH_2_Cl_2_ (20 mL). The solution was washed with water (20 mL), dried over Na_2_SO_4_, filtered and the solvent was removed under reduced pressure to give Fmoc-Lys(Boc)-OSu (1.21 g, 100%) as a white solid. The ^1^H NMR data for the product was in good agreement with those previously reported in the literature (Thiele et al. 2017); R_*f*_ 0.24 (hexane–EtOAc 1:1); IR (film) cm^−1^: 3338, 2932, 2864, 1739, 1673, 1691, 1523, 757, 736, 646, 587; ^1^H NMR (CDCl_3_/MeOD (1 drop), 400 MHz) δ 7.76 (2H, d, *J* 7.7, Ar–H), 7.60 (2H, dt, *J* 7.5, 1.8, Ar–H), 7.39 (2H, td, *J* 7.5, 1.1, Ar–H), 7.31 (2H, td, *J* 7.5, 1.2, Ar–H), 4.77–.66 (1H, m, FmocNHC*H*), 4.49–4.37 (2H, m, CHC*H*_2_), 4.23 (1H, t, *J* 7.0, C*H*CH_2_), 3.13 (2H, t, *J* 6.0, C*H*_2_NHBoc), 2.83 (4H, s, O=CC*H*_2_C*H*_2_C=O), 2.06–1.83 (2H, m, C*H*_a_*H*_b_), 1.60–1.47 (4H, m, CH_2_ × 2), 1.43 (9H, s, *t*-Bu); ^13^C NMR (CDCl_3_, 125 MHz) δ 168.6, 168.2, 156.2, 155.7 (each C=O), 143.6, 141.3, 127.7, 127.1, 125.1, 120.0 (each Ar–C), 79.2 (*C*(CH_3_)_3_), 67.3 (CH*C*H_2_), 52.2 (FmocNH*C*H), 47.1 (*C*HCH_2_), 39.8 (*C*H_2_NHBoc), 31.9 (*C*H_a_H_b_), 29.4 (CH_2_), 28.4 (C(*C*H_3_)_3_), 25.6 (O=C*C*H_2_CH_2_*C*=O), 21.9 (CH_2_); ESI-HRMS calcd. C_30_H_39_N_4_O_8_, 583.2762 found m/z 583.2743 [M + NH_4_]^+^.

#### Fmoc-Lys(Boc)-Lys(***Z***)-O^t^Bu (21)(Thiele et al. 2017)

A suspension of *L*-Lys(Z)-O^t^Bu·HCl (738 mg, 1.98 mmol) in dry CH_2_Cl_2_ (8 mL) was treated with DIPEA (0.35 mL, 1.98 mmol). The resulting mixture was added to a solution of Fmoc-Lys(Boc)-OSu **20** (792 mg, 1.4 mmol) in dry CH_2_Cl_2_ (8 mL) at 0 °C. The mixture was warmed to room temperature and stirred for 16 h under argon, then washed with brine (10 mL, dried over Na_2_SO_4_, filtered and the solvent was removed under reduced pressure. Flash chromatography of the residue (hexane–EtOAc, 2:1–1:2) gave the title compound (1.04 g, 95%) as a white solid; R_*f*_ 0.5 (hexane–EtOAc, 1:2); IR (film) cm^−1^: 3311, 2932, 2864, 1683, 1651, 1531, 1249, 1160, 734, 645; ^1^H NMR (MeOD, 400 MHz) δ 7.80 (2H, d, *J* 7.5, Ar–H), 7.66 (2H, t, *J* 7.6, Ar–H), 7.40 (2H, t, *J* 7.5, Ar–H), 7.36–7.24 (7H, m, each Ar–H), 5.12–4.95 (2H, m, OC*H*_2_C_6_H_5_), 4.37 (2H, d, *J* 6.9, CHC*H*_2_), 4.28 (1H, dd, *J* 8.9, 5.0, NHC*H*), 4.21 (1H, t, *J* 6.9, C*H*CH_2_), 4.12 (1H, dd, *J* 8.8, 5.4, NHC*H*), 3.17–2.99 (4H, m, C*H*_2_NHBoc & C*H*_2_NHCbz), 1.90–1.58 (4H, m, C*H*_a_*H*_b_ & C*H*_a’_*H*_b’_), 1.57–1.32 (26H, m, each CH_2_ × 4 & *t*-Bu × 2); ^13^C NMR (MeOD, 100 MHz) δ 174.8, 172.7, 158.9, 158.5, 158.4 (each C=O), 145.3 (2 s), 142.6, 138.4, 129.4, 128.9, 128.8, 128.7, 128.2 (2 s), 126.2, 120.9 (each Ar–C), 82.8 (*C*(CH_3_)_3_), 79.9 (*C*(CH_3_)_3_), 67.9 (CH*C*H_2_), 67.3 (O*C*H_2_C_6_H_5_), 56.3 (NH*C*H), 54.3 (NH*C*H), 48.5 (*C*HCH_2_), 41.5, 41.1 (*C*H_2_NHBoc & *C*H_2_NHCbz), 32.9, 32.3, 30.5, 30.3 (each CH_2_), 28.8 (C(CH_3_)_3_), 28.3 (C(CH_3_)_3_), 24.1, 23.9 (each CH_2_); ESI-HRMS calcd. C_44_H_62_N_5_O_9_, 804.4542 found m/z 804.4504 [M + NH_4_]^+^._._

#### H_2_N-Lys(Boc)-Lys(*Z*)-OtBu (2)

To a stirred solution of Fmoc-Lys(Boc)-Lys(*Z*)-OtBu **21** (640 mg, 0.81 mmol) in dry CH_2_Cl_2_ (8 mL) was added NHEt_2_ (1.7 mL, 16.3 mmol). The mixture was stirred at room temperature for 5 h under argon. The solvent was removed under reduced pressure and flash chromatography of the residue (CH_2_Cl_2_-MeOH, 100:0–98:2–95:5–90:10) gave **2** (456 mg, 99%) as a colourless oil which spontaneously solidified over time; R_*f*_ 0.4 (CH_2_Cl_2_-MeOH 90:10); IR (film) cm^−1^: 3355, 2933, 2864, 1721, 1686, 1519, 1165, 1245, 723, 697, 631; ^1^H NMR (MeOD, 400 MHz) δ 7.42–7.24 (5H, m, Ar–H), 5.06 (2H, m, OCH_2_C_6_*H*_5_), 4.26 (1H, dd, *J* 8.5, 5.2, NHC*H*), 3.34 (1H, m, NH_2_C*H*), 3.12 (2H, t, *J* 6.8, C*H*_2_NHCbz), 3.04 (2H, t, *J* 6.8, C*H*_2_NHBoc), 1.88–1.34 (30H, m, each CH_2_ × 6 & *t*-Bu × 2); ^13^C NMR (MeOD, 100 MHz) δ 177.5, 172.8, 158.9, 158.5 (each C=O), 138.4, 129.4, 128.9, 128.7 (each Ar–C), 82.9 (*C*(CH_3_)_3_), 79.8 (*C*(CH_3_)_3_), 67.3 (O*C*H_2_C_6_H_5_), 55.8 (NH_2_*C*H), 54.3 (NH*C*H), 41.5 (*C*H_2_NHCbz), 41.1 (*C*H_2_NHBoc), 36.1 (CH_2_), 32.4 (CH_2_), 30.7 (CH_2_), 30.4 (CH_2_), 28.8 (C(CH_3_)_3_), 28.3 (C(CH_3_)_3_), 24.0 (CH_2_), 23.8 (CH_2_); ESI-HRMS calcd. C_29_H_49_N_4_O_7_, 565.3601 found m/z 565.3618 [M + H]^+^.

#### 4,4-difluoro-8-(4-(succinimidocarboxy)phenyl)-1,3,5,7-tetramethyl-4-bora-3a,4a-diaza-s-indacene (1)(Brellier et al. 2010)

*N*-hydroxysuccinimide (248 mg, 2.16 mmol) and EDC (414 mg, 2.16 mmol) were added to a solution of 4,4-difluoro-8-(4-carboxyphenyl)-1,3,5,7-tetramethyl-4-bora-3a,4a-diaza-s-indacene acid (Brellier et al. 2010) (918 mg, 2.16 mmol) in dry CH_2_Cl_2_ (45 mL). After stirring the reaction mixture for 16 h under argon, the solvent was removed under reduced pressure and the resulting residue was dissolved in CH_2_Cl_2_ (20 mL). The solution was washed with water, dried over Na_2_SO_4_, filtered and the solvent was removed under reduced pressure. Flash chromatography of the residue (CH_2_Cl_2_, 100%) gave **1** (662 mg, 59%) as a red–orange solid. The ^1^H and ^13^C NMR data for **1** was in good agreement with those previously reported in the literature (Brellier et al. 2010); R_*f*_ 0.24 (CH_2_Cl_2_ 100%); IR (film) cm^−1^: 2967, 2929, 2868, 1763, 1740, 1538, 1187, 976, 727, 533; ^1^H NMR (CDCl_3_, 400 MHz) δ 8.27 (1H, d, *J* 8.6, Ar–H), 7.50 (1H, d, *J* 8.6, Ar–H), 2.96 (4H, s, O=CC*H*_2_C*H*_2_C=O), 2.54 (6H, s, CH_3_ × 2), 2.31 (4H, q, *J* 7.5, CH_2_C*H*_3_), 1.28 (6H, s, CH_3_ × 2), 0.99 (6H, t, *J* 7.5, CH_2_C*H*_3_); ^13^C NMR (CDCl_3_, 125 MHz) δ 169.2, 161.4 (each C=O), 154.6, 143.0, 138.0, 137.7, 133.3, 131.2, 130.1, 129.3, 125.6 (each Ar–C), 25.7 (O=C*C*H_2_*C*H_2_C=O), 17.0 (*C*H_2_CH_3_), 14.6 (CH_2_*C*H_3_), 12.6, 12.1 (each CH_3_); ESI-HRMS calcd. C_28_H_34_BF_2_N_4_O_4_, 539.2641 found m/z 539.2627 [M + H]^+^.

#### 4,4-dicyano-8-(4-(succinimidocarboxy)phenyl)-2,6-diethyl-1,3,5,7-tetramethyl-4-bora-3a,4a-diaza-s-indacene (7)

To a stirred solution of **1** (100 mg, 0.192 mmol) and BF_3_^.^OEt (0.24 mL, 1.92 mmol) in dry CH_2_Cl_2_ (20 mL) was added TMS-CN (0.48 mL, 3.84 mmol). The reaction mixture was stirred in the dark at room temperature for 1 h. EtOAc (40 mL) was added and the organic later was washed with water (20 mL), brine (20 mL), dried over Na_2_SO_4_ and the solvent was removed under reduced pressure. Flash chromatography of the residue (hexane–EtOAc, 6:4–1:1) gave **7** (79 mg, 77%) as a red–orange solid; R_*f*_ 0.4 (hexane–EtOAc, 1:1); IR (film) cm^−1^: 2956, 2923, 2854, 1768. 1740, 1540, 1474, 1185, 977, 726, 544; ^1^H NMR (CDCl_3_, 400 MHz) δ 8.31 (2H, d, *J* 8.3, Ar–H), 7.51 (2H, d, *J* 8.3, Ar–H), 2.96 (4H, s, O=CC*H*_2_C*H*_2_C=O), 2.71 (6H, s, CH_3_ × 2), 2.37 (4H, q, *J* 7.6, C*H*_2_CH_3_), 1.32 (6H, s, CH_3_ × 2), 1.02 (6H, t, *J* 7.6 CH_2_C*H*_3_); ^13^C NMR (CDCl_3_, 100 MHz, CN signals were not observed) δ 169.1, 161.2 (each C=O), 155.0, 141.8, 139.5, 138.6, 134.8, 131.4, 129.1, 128.6, 126.2 (each Ar–C), 25.7 (O=C*C*H_2_*C*H_2_C=O), 17.2 (*C*H_2_CH_3_), 14.4 (CH_2_*C*H_3_), 13.5, 12.3 (each CH_3_); ESI-HRMS calcd. C_30_H_31_BN_5_O_4_, 536.2469 found m/z 536.2467 [M + H]^+^.

#### *tert*-butyl *N*^6^-((benzyloxy)carbonyl)-*N*^2^-(*N*^6^-(tert-butoxycarbonyl)-*N*^2^-(4-(4,4-dicyano-2,6-diethyl-1,3,5,7-tetramethyl-4-bora-3a,4a-diaza-*s*-indacen-8-yl)benzoyl)-*L*-lysyl)-*L*-lysinate (8)

A solution of the amine **2** (168 mg, 0.3 mmol) and triethylamine (41 µL, 0.3 mmol) in dry CH_2_Cl_2_ (1.5 mL) was added under argon to a solution of the succinimide **7** (79 mg, 0.15 mmol) in dry CH_2_Cl_2_ (1.5 mL). The reaction mixture was stirred in the dark at room temperature for 4 days. The solvent was removed under reduced pressure and flash chromatography of the residue (CH_2_Cl_2_-CH_3_CN 4:1–2:1) gave **8** (116 mg, 80%) as a red–orange solid; R_*f*_ 0.3 (EtOAc-hexane 1:1); IR (film) cm^−1^: 2969, 2978, 2863, 1699, 1643, 1541, 1188, 1153, 734, 544; ^1^H NMR (MeOD, 500 MHz) δ 8.08 (2H, d, *J* 8.3, Ar–H), 7.41 (2H, d, *J* 8.1, Ar–H), 7.35–7.19 (5H, m, OCH_2_C_6_*H*_5_), 5.07 (1H, d, *J* 12.6, OC*H*HC_6_H_5_), 5.03 (1H, d, *J* 12.6, OCH*H*C_6_H_5_), 4.59 (1H, dd, *J* 8.8, 5.9, NHC*H*), 4.32 (1H, dd, *J* 9.1, 4.9, NHC*H*), 3.17–3.02 (4H, m, C*H*_2_NHBoc & C*H*_2_NHCbz), 2.68 (6H, s, CH_3_ × 2), 2.44 (4H, q, *J* 7.5, C*H*_2_CH_3_), 1.99–1.81 (3H, m, C*H*_a_*H*_b_ & C*H*_a’_H_b’_), 1.77–1.66 (1H, m, CH_a’_*H*_b’_), 1.621.40 (26H, m, each CH_2_ × 4 & *t*-Bu × 2), 1.37 (6H, s, CH_3_ × 2), 1.03 (6H, t, *J* 7.5, CH_2_C*H*_3_); ^13^C NMR (MeOD, 125 MHz, CN signals were not observed) δ 174.5, 172.8, 169.3, 158.9, 158.6 (each C=O), 155.5, 142.1, 141.6, 139.2, 138.5, 136.4, 136.1, 130.2, 129.9, 129.8, 129.4, 128.9, 128.8 (each Ar–C), 82.8 (*C*(CH_3_)_3_), 79.9 (*C*(CH_3_)_3_), 67.3 (O*C*H_2_C_6_H_5_), 55.5 (NH*C*H), 54.4 (NH*C*H), 41.6, 41.1 (*C*H_2_NHBoc & *C*H_2_NHCbz), 32.7, 32.2, 30.7, 30.3 (each CH_2_), 28.8 (C(CH_3_)_3_), 28.3 (C(CH_3_)_3_), 24.5, 24.0 (each CH_2_), 17.9 (*C*H_2_CH_3_), 14.8 (CH_2_*C*H_3_), 13.5 (CH_3_), 12.5 (CH_3_); ESI-HRMS calcd. C_55_H_74_BN_8_O_8_, 985.5723 found m/z 985.5747[M + H]^+^.

#### *N*^6^-((benzyloxy)carbonyl)-*N*^2^-(4-(4,4-dicyano-2,6-diethyl-1,3,5,7-tetramethyl-4-bora-3a,4a-diaza-s-indacen-8-yl)benzoyl)-*L*-lysyl)-*L*-lysine (9)

To a solution of **8** (50 mg, 51 µmol) in dry CH_2_Cl_2_ (1.6 mL) was added TFA (0.4 mL) dropwise. The reaction mixture was stirred in the dark at room temperature for 6 h. The solvent was removed under a stream of air and the resulting residue was washed with three 10 mL portions of Et_2_O to give the amine **9** (48 mg, 100%) as a red orange solid; ^1^H NMR (MeOD, 500 MHz) δ 8.09 (2H, d, *J* 8.1, Ar–H), 7.43 (2H, d, *J* 8.1, Ar–H), 7.36–7.21 (5H, m, OCH_2_C_6_*H*_5_), 5.09 (1H, d, *J* 12.5, OC*H*HC_6_H_5_), 5.05 (1H, d, *J* 12.5, OCH*H*C_6_H_5_), 4.65 (1H, t, *J* 7.2, NHC*H*), 4.48 (1H, dd, *J* 9.5, 4.4, NHC*H*), 3.19–3.07 (2H, m, CH_2_), 2.99 (2H, t, *J* 7.6, CH_2_), 2.70 (6H, s, CH_3_ × 2), 2.46 (4H, q, *J* 7.5, C*H*_2_CH_3_), 2.05–1.87 (3H, m, CH_2_ & CH), 1.82–1.73 (3H, m, CH_2_ & CH), 1.65–1.47 (6H, m, each CH_2_), 1.39 (6H, s, CH_3_ × 2), 1.05 (6H, d, *J* 7.5, CH_2_C*H*_3_); ^13^C NMR (MeOD, 125 MHz, CN signals were not observed) δ 175.3, 174.2, 169.3, 158.9 (each C=O), 155.5, 141.8, 141.6, 139.4, 138.6, 136.3, 136.1, 130.1, 129.9, 129.8, 129.4, 128.9, 128.7 (each Ar–C), 67.3 (O*C*H_2_C_6_H_5_), 55.2 (NH*C*H), 53.5 (NH*C*H), 41.6, 40.6 (*C*H_2_NH_2_ & *C*H_2_NHCbz), 32.3, 32.2, 30.3, 28.2, 24.1, 23.8 (each CH_2_), 17.9 (*C*H_2_CH_3_), 14.8 (CH_2_*C*H_3_), 13.5 (CH_3_), 12.5 (CH_3_); ESI-HRMS calcd. C_46_H_58_BN_8_O_6_, 829.4572 found m/z 829.4554 [M + H]^+^.

#### *N*^6^-((benzyloxy)carbonyl)-*N*^2^-(*N*^6^-(2-(4-(1,2,4,5-tetrazin-3-yl)phenyl)acetyl)-*N*^2^-(4-(4,4-dicyano-2,6-diethyl-1,3,5,7-tetramethyl-4-bora-3a,4a-diaza-s-indacen-8-yl)benzoyl)-*L*-lysyl)-*L*-lysine (11)

After drying under high vacuum for 2 h the amine **9** (48 mg, 51 µmol) was dissolved in dry CH_2_Cl_2_ (4 mL) and DIPEA (88 µL, 510 µmol) was added. Tetrazine **10** (19 mg, 61.2 µmol) was added in one portion and the reaction mixture was stirred in the dark at room temperature for 2 h. The mixture was diluted with CH_2_Cl_2_ (30 mL)_,_ washed with 0.5 M HCl (10 mL), water (10 mL), brine (10 mL), dried over Na_2_SO_4_, filtered and the solvent was removed under reduced pressure. Flash chromatography of the residue (100% CH_2_Cl_2_–CH_2_Cl_2_-MeOH 95:5–80:20) gave **11** (47 mg, 90% for two steps) as a red solid; R_*f*_ 0.54 (CH_2_Cl_2_-MeOH, 9:1); IR (film) cm^−1^: 3313. 2931, 1547, 1190, 1155, 981, 736; ^1^H NMR (MeOD, 500 MHz) δ 10.29 (1H, s, Tetrazine-H), 8.53 (2H, d, *J* 8.3, Ar–H), 8.07 (2H, d, *J* 8.1, Ar–H), 7.57 (2H, d, *J* 8.3, Ar–H), 7.39 (2H, d, *J* 8.1, Ar–H), 7.33–7.17 (5H, m, each Ar–H), 5.06 (1H, d, *J* 12.6, OC*H*HC_6_H_5_), 5.02 (1H, d, *J* 12.6, OCH*H*C_6_H_5_), 4.59–4.55 (1H, m, NHC*H*), 4.40–4.34 (1H, m, NHC*H*), 3.65 (2H, s, CH_2_), 3.28–3.24 (2H, m), 3.14–3.06 (2H, m), 2.68 (6H, s, CH_3_ × 2), 2.42 (4H, d, *J* 7.6, C*H*_2_CH_3_), 2.00–1.82 (3H, m, C*H*_a_*H*_b_ & C*H*_a’_H_b’_), 1.79–1.70 (1H, m, CH_a’_*H*_b’_), 1.66–1.41 (6H, m, each CH_2_), 1.34 (6H, s, CH_3_ × 2), 1.02 (6H, t, *J* 7.6, CH_2_C*H*_3_); ^13^C NMR (125 MHz, MeOD, CN signals were not observed) δ 174.0 (2 s), 173.1, 168.8 (each C=O), 167.5 (Tetrazine-C), 161.9 (Tetrazine-C), 158.8 (C=O), 155.4, 143.4, 142.0, 141.4, 139.8, 138.4, 137.2, 136.1, 131.9, 131.2, 130.1, 129.9, 129.8, 129.7, 129.4, 129.3, 128.9, 128.7 (each Ar–C), 67.3 (O*C*H_2_C_6_H_5_), 55.5 (NH*C*H), 52.6 (NH*C*H), 43.8 (*C*H_2_C_8_H_5_N_4_), 41.7 (*C*H_2_NHCbz), 39.9 (*C*H_2_NHCO), 32.4 (2 s), 30.3, 29.7, 24.4, 24.1 (each CH_2_), 17.9 (*C*H_2_CH_3_), 14.8 (CH_2_*C*H_3_), 13.6 (CH_3_), 12.6 (CH_3_); ESI-HRMS calcd. C_56_H_62_BN_12_O_7_, 1025.4957 found m/z 1025.4982 [M-H]^−^.

#### *N*^2^-(*N*^6^-(2-(4-(1,2,4,5-tetrazin-3-yl)phenyl)acetyl)-*N*^2^-(4-(4,4-dicyano-2,6-diethyl-1,3,5,7-tetramethyl-4-bora-3a,4a-diaza-s-indacen-8-yl)benzoyl)-*L*-lysyl)-*L*-lysine (12)

To a stirred solution of **11** (80 mg, 77.9 µmol) in EtOH (44 mL) and formic acid (88 µL) was added 10% palladium on carbon (144 mg). The reaction flask was stoppered and flushed with argon for 1 min followed by hydrogen for a further 1 min. The mixture was stirred at room temperature in the dark under hydrogen (balloon) for 6 h, filtered through Celite and the solvent was removed under reduced pressure. The residue was washed with three 10 mL portions of each Et_2_O, EtOAc and hexane to give the formate salt of **12** (52.5 mg, 72%) as a red solid which was taken to the next step without further purification. A portion of the crude residue was purified via C-18 column (H_2_O (0.1% TFA)–ACN (0.1% TFA) 35:75–45:55) to give the TFA salt of **12** for characterisation. *Important note: prior to obtaining NMR data of the crude formate salt, the solution of the compound in MeOD is heated to 40 °C for 15 min to ensure complete conversion of exchangeable hydrogens with deuterium. The exchange was found to be slow at room temperature and, if not given the appropriate time to exchange, can result in an artifact where multiple compounds are present in the NMR simultaneously;* ^1^H NMR (MeOD, formate salt, 500 MHz) δ 10.31 (1H, s, Tetrazine-H), 8.56 (2H, d, *J* 8.3, Ar–H), 8.52 (1H, s, *H*COOH), 8.11 (2H, d, *J* 8.3, Ar–H), 7.60 (2H, d, *J* 8.3, Ar–H), 7.53 (2H, d, *J* 8.3, Ar–H), 4.53 (1H, dd, *J* 8.9, 5.6, NHC*H*), 4.33 (1H, dd, *J* 7.7, 4.9, NHC*H*), 3.68 (2H, s, CH_2_), 3.29 (2H, t, *J* 6.6, CH_2_), 2.95 (2H, t, *J* 7.4, CH_2_), 2.70 (6H, s, CH_3_ × 2), 2.45 (4H, q, *J* 7.6, C*H*_2_CH_3_), 2.00–1.50 (12H, m, each CH_2_), 1.39 (6H, s, CH_3_ × 2), 1.04 (6H, t, *J* 7.6, CH_2_C*H*_3_); ^1^H NMR (MeOD, TFA salt, 400 MHz) δ 10.29 (1H, s, Tetrazine-H), 8.53 (2H, d, *J* 8.0, Ar–H), 8.09 (2H, d, *J* 8.0, Ar–H), 7.57 (2H, d, *J* 8.0, Ar–H), 7.51 (2H, d, *J* 8.0, Ar–H), 4.50 (2H, m, NHC*H* × 2), 3.65 (2H, s, CH_2_), 3.28 (2H, d, *J* 7.0, CH_2_), 2.97 (2H, t, *J* 7.5, CH_2_), 2.68 (6H, s, CH_3_ × 2), 2.43 (4H, q, *J* 7.6, C*H*_2_CH_3_), 2.04–1.51 (12H, m, each CH_2_), 1.37 (6H, s, CH_3_ × 2), 1.02 (6H, t, *J* 7.5, CH_2_C*H*_3_); ^13^C NMR (MeOD, TFA Salt, 100 MHz, CN signals were not observed) δ 175.0, 174.7, 173.2, 169.5 (each C=O), 167.6 (Tetrazine-C), 159.2 (Tetrazine-C), 155.6, 142.6, 141.8, 139.4, 136.8, 136.3, 132.0, 131.2, 130.2, 129.9 (2 s), 129.3 (each Ar–C), 55.9, 53.0 (each NH*C*H), 43.8 (*C*H_2_C_8_H_5_N_4_), 40.6, 40.3 (*C*H_2_NHCO & *C*H_2_NH_2_), 32.4, 32.0, 30.1, 28.6, 24.4, 23.7 (each CH_2_), 17.9 (*C*H_2_CH_3_), 14.8 (CH_2_*C*H_3_), 13.5 (CH_3_), 12.5 (CH_3_); ESI-HRMS calcd. C_48_H_58_BN_12_O_5_, 893.4746 found m/z 893.4748 [M-H]^−^.

#### 2,2′,2″-(10-(2-(((*S*)-5-((*S*)-6-(2-(4-(1,2,4,5-tetrazin-3-yl)phenyl)acetamido)-2-(4-(4,4-dicyano-2,6-diethyl-1,3,5,7-tetramethyl-4-bora-3a,4a-diaza-s-indacen-8-yl)benzamido)hexanamido)-5-carboxypentyl)amino)-2-oxoethyl)-1,4,7,10-tetraazacyclododecane-1,4,7-triyl)triacetic acid (13)

To a stirred solution of **12** (14 mg, 14.9 µmol) in dry CH_2_Cl_2_ (2 mL) were added NEt_3_ (22 µL, 157 µmol) and DOTA-NHS (13 mg, 17.3 µmol). The reaction mixture was stirred at room temperature for two hours and the solvent was removed under a stream of air. The resulting residue was purified via a Biotage C-18 column (H_2_O (0.1% TFA)–ACN (0.1% TFA) 80:20–50:50) to give the tile compound **13** (13.5 mg, 52%) as a pink-red solid; R_*f*_ 11.2 min (C-18 column, flow rate = 2 mL/min, H_2_O (0.1% TFA)–ACN (0.1% TFA) 70:30–50:50 over 15 min); ^1^H NMR (CD_3_CN-D_2_O, 600 MHz) δ 10.23 (1H, s, Tetrazine-H), 8.40 (2H, d, *J* 8.4, Ar–H), 7.97 (2H, d, *J* 8.4 Ar–H), 7.49 (2H, d, *J* 8.4 Ar–H), 7.39 (2H, d, *J* 8.5Ar-H), 4.52 (1H, dd, *J* 9.1, 5.0, NHC*H*), 4.25 (1H, dd, *J* 8.0, 5.5 NHC*H*), 3.70–2.95 (30H, m, each CH_2_), 2.57 (6H, s, CH_3_ × 2), 2.30 (4H, q, *J* 7.6, C*H*_2_CH_3_), 1.79–1.66 (3H, m, C*H*_a_*H*_b_ & C*H*_a’_H_b’_), 1.51–1.30 (9H, m, CH_a’_*H*_b’_ & CH_2_), 1.24 (6H, s, CH_3_ × 2) 0.90 (6H, t, *J* 7.6, CH_2_C*H*_3_); ^1^H NMR (MeOD, 600 MHz) δ 10.30 (1H, s, Tetrazine-H), 8.54 (2H, d, *J* 8.3 Ar–H), 8.09 (2H, d, *J* 7.8 Ar–H), 7.57 (2H, d, *J* 8.3 Ar–H), 7.52 (2H, d, *J* 7.9 Ar–H), 4.56 (1H, dd, *J* 8.9, 5.5 NHC*H*), 4.43 (1H, dd, *J* 9.3, 4.8 NHC*H*), 4.07–2.92 (30H, m, each CH_2_), 2.68 (6H, s, CH_3_ × 2), 2.43 (4H, q, *J* 7.6, C*H*_2_CH_3_), 1.99–1.87 (3H, m, C*H*_a_*H*_b_ & C*H*_a’_H_b’_), 1.79–1.73 (1H, m, CH_a’_*H*_b’_), 1.66–1.45 (8H, m, each CH_2_), 1.37 (6H, s, CH_3_ × 2), 1.02 (6H, t, *J* 7.6, CH_2_C*H*_3_); ^13^C NMR (MeOD, 150 MHz, DOTA signals were not observed) δ 175.4, 174.7, 173.3, 169.4 (each C=O), 167.6 (Tetrazine-C), 161.8 (q, *J* 36.8, CF_3_*C*O_2_H) 158.7 (Tetrazine-C), 155.5, 142.7, 142.0, 141.5, 139.3, 136.4, 136.1, 132.0, 131.2, 130.1, 129.9, 129.9, 129.3 (each Ar–C), 127.5 (q, *J* 75, N*C*-B) 117.6 (q, *J* 290.2, *C*F_3_CO_2_H), 55.6 (NH*C*H), 53.5 (NH*C*H), 44.3 (*C*H_2_C_8_H_5_N_4_), 40.3 (2 s) (*C*H_2_NHCO & *C*H_2_NHCO), 32.5, 32.2, 30.1, 29.7, 25.7, 24.3 (each CH_2_), 17.9 (*C*H_2_CH_3_), 14.9 (CH_2_*C*H_3_), 13.5 (CH_3_), 12.6 (CH_3_); ESI-HRMS calcd. C_64_H_84_BN_16_O_12_, 1279.6542 found m/z 1279.6548 [M + H]^+^.

#### 2,2′,2′′-(10-(2-(((*S*)-5-carboxy-5-((*S*)-2-(4-(5-cyano-2,8-diethyl-5-isocyano-1,3,7,9-tetramethyl-5*H*-4*λ*^4^,5*λ*^4^-dipyrrolo[1,2-*c*:2′,1′-f][1,3,2]diazaborinin-10-yl)benzamido)-6-(2-(4-((7S,10a*R*)-7-((((2,5-dioxopyrrolidin-1-yl)oxy)carbonyl)oxy)-3,5,6,7,8,9,10,10a-octahydrocycloocta[d]pyridazin-1-yl)phenyl)acetamido)hexanamido)pentyl)amino)-2-oxoethyl)-1,4,7,10-tetraazacyclododecane-1,4,7-triyl)triacetic acid (17)

The stock solution for fluorescent measurements was prepared as follows; **13** (0.3 mg, 0.172 µmol) was dissolved in DMSO (200 µL) then 20 µL of TCO-NHS (1.5 mg, 5.61 µmol in 200 µL DMSO) was added. The reaction mixture was left to stand at room temperature for 1 h. The solution colour changed from pink to orange indicting tetrazine consumption. The product and reaction completion was confirmed via HRMS. ESI-HRMS calcd. C_77_H_101_BN_15_O_17_, 1518.7593 found m/z 1518.7587 [M + H]^+^.

#### Indium complex (14)

In a 2 mL centrifuge tube **13** (1.8 mg, 1.04 µmol) was dissolved in MeOH (1 mL). NEt_3_ (10 µL) and anhydrous Indium chloride (2.3 mg, 10.8 µmol) was added. The reaction mixture was heated at 50 °C for 5 min during which time a precipitate formed. The suspension was centrifuged at 15,000 rpm for 2 min, the supernatant was removed and the precipitate was washed with three 2 mL portions of MeOH to give **14** (1.45 mg, quant.) as a red solid; ESI-HRMS calcd. C_64_H_81_BInN_16_O_12_, 1391.5352 found m/z 1391.5300 [M + H]^+^.

#### Lanthanum complex (15)

**13** (0.5 mg, 0.28 µmol) was dissolved in sodium acetate buffer (1 mL, 100 mM pH = 6) and Lanthanum chloride hydrate (1 mg, 4.08 µmol) was added. The reaction mixture was heated at 45 °C for 60 min during which time a precipitate formed. The suspension was transferred to a 2 mL centrifuge tube and centrifuged at 15,000 rpm for 2 min. The supernatant was removed and the precipitate was washed with three 2 mL portions of water. Lyophilisation gave **15** (0.4 mg, quant.) as a red solid; ESI-HRMS calcd. C_64_H_81_BLaN_16_O_12_, 1415.5376 found m/z 1415.5389 [M + H]^+^.

### Preparation of trastuzumab-TCO and conjugation with DO3A-BODIPY-Tz

#### TCO conjugation to trastuzumab

Procedures followed closely those previously published (Membreno et al. 2019). Purified trastuzumab (Herceptin) (5.0 mg, 47.9 mg/mL) in PBS buffer (pH 7.4) was adjusted to pH 8.5 using small aliquots (1–5 µL) of Na_2_CO_3_ solution (0.1 M) in a 1.5 mL Eppendorf tube. To the antibody solution, TCO-NHS (40 mg/mL solution in DMF, 40 mol equiv.) was added slowly with agitation. The reaction mixture was incubated at 25 °C on a thermomixer for 1 h with mild agitation (500 rpm), and subsequently purified using PD-10 desalting columns (GE Healthcare) and collected in PBS (pH 7.4, 2 × 1 mL). The concentration of trastuzumab-TCO was measured using a Nanodrop UV–Vis spectrophotometer monitoring the 280 nm wavelength (ɛ_280_ = 210,000 M^−1^ cm^−1^), and the number of TCO moieties per antibody was determined by MALDI-ToF MS/MS to be 4.3.

#### DOTA-BODIPY-Tz and trastuzumab-TCO in vitro “click”

To a solution of trastuzumab-TCO (100 μg in 500 μL PBS), DOTA-BODIPY-Tz (**13**) (35 μL of 1.3 × 10^–3^ M solution in DMSO, ~ 70 equiv.) was added slowly with agitation. The reaction mixture was rotated at ambient temperature for 1 h, and subsequently purified using PD-10 desalting columns (GE Healthcare) and collected in PBS (pH 7.4, 2 mL). The number of DOTA-BODIPY-Tz moieties ‘clicked’ per antibody was determined by MALDI-ToF MS/MS to be 2.9.

### ^111^In & ^225^Ac radiolabeling and radiometal complex stability studies

#### General Methods and Instrumentation

[^111^In]InCl_3_ was purchased from BWXT (Vancouver, BC, Canada) and received as ~ 0.05 N HCl solution. [^225^Ac]Ac(NO_3_)_3_ was produced via the spallation of thorium targets on TRIUMF’s 500 MeV cyclotron (Vancouver, BC, Canada), and isolated as previously described(Robertson et al. 2020) in dilute acid (~ 0.05 M HNO_3_). Aluminum-backed TLC plates (silica gel 60, F_254_, EMD Millipore) or paper-backed instant TLC plates (silica gel, iTLC-SG, Agilent) were used to analyze ^225^Ac or ^111^In radiolabeling reaction progress, respectively. TLC plates were developed and then were measured on a BioScan System 200 imaging scanner equipped with a BioScan Autochanger 1000 and WinScan software and radiolabeling yields were calculated by integrating the peaks in the radio-chromatogram. For ^225^Ac radiolabeling, developed plates were counted at least 8 h later to allow for daughter isotopes to decay completely, to ensure the radioactive signal was generated solely by parent ^225^Ac.

The radioactive RP-HPLC system used to analyse ^111^In radiolabeling yields consisted of a Phenomenex Luna C18(2) 100 Å RP analytical column (5 μm, 100 × 4.6 mm) using an Agilent HPLC equipped with a model 1200 quaternary pump, a model 1200 UV absorbance detector (set at 250 nm) and a Raytest Gabi Star NaI(Tl) radiation detector.

#### ^111^In radiolabeling studies

Radiolabeling procedures followed closely those outline previously (Ramogida et al. 2015; Spreckelmeyer et al. 2017; Comba et al. 2017). Briefly, DO3A-BODIPY-Tz (**13·4TFA**) was made up as a stock solution (1 mg mL^−1^, 5.76 × 10^–3^ M) in DMSO. Using serial dilution, stock ligand solutions with concentrations of 5.76 × 10^–4^–10^–6^ M were also prepared in deionized water. A 10 µL aliquot of each ligand stock solution (or 10 µL of deionized water as a blank) was added to a Eppendorf tube and diluted with ammonium acetate buffer (0.15 M, pH 5) such that the final reaction volume was 100 µL after the addition of [^111^In]InCl_3_, to give final ligand concentrations of 5.76 × 10^–4^–10^–7^ M. An aliquot of [^111^In]InCl_3_ (3.7–37 MBq) was added to the Eppendorf tubes containing ligand and buffer and allowed to react for 30–60 min at 45 °C. Reaction progress was analyzed at 30 and 60 min by spotting a small aliquot (1–3 µL) onto the bottom of an instant thin layer chromatography (iTLC-SG) plate and developed using mobile phase (MP) A (EDTA, 50 mM, pH 5). Under these conditions, uncomplexed [^111^In]In^3+^ travels with the solvent front (*R*_*f*_ ~ 1) while [^111^In]In-complexed species stick to the baseline (*R*_*f*_ = 0). Alternatively, radiolabeling yields were determined by analytical RP-HPLC. Elution conditions used for RP-HPLC analysis were gradient: A: 0.1% TFA in water; B: 0.1% TFA in acetonitrile; 0 to 100% B linear gradient 20 min, 1 mL min^−1^. [^111^In][In-DO3A-BODIPY-Tz] (*t*_*R*_ = 11.6 min), “free” ^111^In^3+^ (*t*_*R*_ = 1.56 min). According to the radiolabeling yield observed, the ^111^In-complexes were either purified using a C_18_-light cartridge (Waters Sep-pak, pre-conditioned with 5 mL ethanol, then 10 mL of labeling buffer) eluted with 300 μL of ethanol or used without further purification.

#### Conjugation of [^111^In]In-DO3A-BOPIDY-Tz to trastuzumab-TCO

The [^111^In]In-DO3A-BOPIDY-Tz chelation reaction was mixed with a solution of trastuzumab-TCO (200 µg, in 200 µL PBS pH 7.4). The reaction mixture was agitated at ambient temperature, the progress of the radioconjugation was determined by iTLC-SG developed using MP B: ethanol/water (50:50). Under these conditions, [^111^In]In-DO3A-BOPIDY-Tz travels up the plate (*R*_*f*_ ~ 0.5) while the conjugated antibody [^111^In]In-DO3A-BOPIDY-trastuzumab remains at the baseline (*R*_*f*_ = 0). The reaction mixture was purified by passage over a PD-10 desalting column (GE Healthcare) using PBS (pH 7.4) as mobile phase. Radiochemical purity was analyzed by iTLC-SG using MP B.

#### ^225^Ac radiolabeling studies

Radiolabeling procedures followed closely those outline previously (Thiele et al. 2017; Ramogida et al. 2019). A 1 × 10^–3^ M stock solution of DO3A-BODIPY-Tz (**13·4TFA**) was prepared in DMSO, and serial dilutions in deionized water were prepared to give additional solutions of 10^–4^ and 10^–5^ M. A 10 µL aliquot of each ligand stock solution (or 10 µL of deionized water as a blank) was added to a Eppendorf tube in triplicate and diluted with ammonium acetate buffer (0.15 M, pH 7) such that the final reaction volume was 100 µL after the addition of [^225^Ac]Ac(NO_3_)_3_, to give final ligand concentrations of 1 × 10^–4^–10^–6^ M. An aliquot of [^225^Ac]Ac(NO_3_)_3_ (37–100 kBq) was added to the Eppendorf tubes containing ligand and buffer and allowed to react for 30–60 min at 80 °C. Reaction progress was analyzed at 30 and 60 min by spotting a small aliquot (1–3 µL) onto the bottom of an aluminum-backed TLC silica gel plate and developed using MP C (citric acid, 0.4 M, pH 4). Under these conditions, uncomplexed [^225^Ac]Ac^3+^ travels with the solvent front (*R*_*f*_ ~ 1) while [^225^Ac]Ac-complexed species stick to the baseline (*R*_*f*_ = 0). According to the radiolabeling yield observed, the ^225^Ac-complexes were either purified using a C_18_-light cartridge (Waters Sep-pak; pre-conditioned with 5 mL ethanol, then 10 mL of labeling buffer) eluted with 300 μL of ethanol or used without further purification.

#### Conjugation of [^225^Ac]Ac-DO3A-BOPIDY-Tz to trastuzumab-TCO

The [^225^Ac]Ac-DO3A-BOPIDY-Tz chelation reaction (containing either 17 or 1.7 μg of ligand), taken directly after radiolabeling or post-C_18_ purificaion, was mixed with varying amounts of trastuzumab-TCO (748–14.7 µg, in PBS pH 7.4) to give ligand-to-antibody ratios of 2:1, 4:1, and 10:1; the reaction volume was set to 1 mL by the addition of PBS (pH 7.4). The reaction mixture was agitated at 37 °C, the progress of the radioconjugation was determined by iTLC-SG developed using MP B: ethanol/water (50:50). Under these conditions, [^225^Ac]Ac-DO3A-BOPIDY-Tz travels up the plate (*R*_*f*_ ~ 1) while the conjugated antibody [^225^Ac]Ac-DO3A-BOPIDY-trastuzumab remains at the baseline (*R*_*f*_ = 0). The reaction mixture was purified by passage over a PD-10 desalting column (GE Healthcare) using PBS (pH 7.4) as mobile phase. Radiochemical purity (%RCP) was analyzed by iTLC-SG using MP B.

#### Radiometal-complex stability studies in human serum

Pre-formed [^111^In]In- and [^225^Ac]Ac-DO3A-BODIPY-Tz species (RCP > 99%) or radiolabeling controls (water was substituted for ligand) were incubated in human serum (1:1 volume based on labeling reaction volume), and the solutions were agitated (450 rpm) at 37 °C. The solutions were monitored over the course of 5–6 days by TLC. For competition studies with ^111^In, iTLC-SG plates using MP B was employed. Under these conditions, [^111^In]In-DO3A-BODIPY-Tz travels up the plate (R_f_ ~ 1), while ^111^In^3+^ that has transchelated to serum proteins remains at the baseline (R_f_ = 0). For competition studies with ^225^Ac, aluminum-backed TLC silica gel plates using MP C was employed. Under these conditions, [^225^Ac]Ac-DO3A-BODIPY-Tz remains at the baseline (R_f_ ~ 0), while uncomplexed ^225^Ac^3+^ that has detached from the ligand travels with the solvent front (R_f_ = 1).

#### Preparation of [^111^In]In-DO3A-BODIPY-trastuzumab for in vivo studies

To a solution of DO3A-BODIPY-Tz (9.25 μg) in ammonium acetate (0.1 M, pH 5.5), was added [^111^In]InCl_3_ (272 MBq in 0.01 M HCl), such that the final reaction volume was 100 μL. The reaction mixture was reacted with agitation (400 rpm) at 45 °C for 45 min. Radiochemical conversion yield (%RCC) was determined to be > 99% by iTLC-SG (0.5 μL spot) using MP A. The radiolabeling reaction was added directly to a solution of trastuzumab-TCO (400 μg, 2:1 ligand-to-mAb ratio) in PBS (900 μL, pH 7.4), and reacted with agitation (400 rpm) at 37 °C for 60 min. The radioconjugation progress was assessed by iTLC-SG using MP B, and the reaction mixture was subsequently purified over a PD-10 desalting column (GE Healthcare) and collected in 2.0 mL of PBS (pH 7.4). The radiochemical purity (RCP) was determined to be > 99% by iTLC-SG using MP B. The specific activity of the final radiotracer (0.3 MBq/μg) was determined by measuring the activity in a dose calibrator and considering a recovery of 80% of the mAb after the PD-10 desalting column.

### In vivo and Ex vivo biodistribution and immuno-SPECT imaging studies

#### SKOV-3 xenograft mouse model

All experiments were conducted in accordance with the guidelines established by the Canadian Council on Animal Care and approved by the Animal Ethics Committee of the University of British Columbia (protocol no. A20-0113). Female nude mice (8 weeks old) obtained from Jackson laboratory (stock#002,019 from JAX) were subcutaneously injected with 8 × 10^6^ SKOV-3 cells in Matrigel (BD Bioscience, 1:1 PBS:matrigel) on the left shoulder.

#### In vivo biodistribution and immuno-SPECT imaging

Mice bearing SKOV-3 ovarian cancer xenografts were administered with 4.1 ± 0.1 MBq (26.3 ± 0.3 µg; *n* = 4) of [^111^In]In-DO3A-BODIPY-Tz-TCO-trastuzumab in ~ 100 µL of PBS (pH 7.4) via tail-vein injection. Mice were imaged 1, 3, or 5 days after injection. Image acquisition and reconstruction was performed using the U-SPECT-II-CT (MILabs, Utrecht, The Netherlands). Prior to image acquisition, mice were anesthetized via inhalation of 2% isoflurane-oxygen gas mixture and placed on the scanner bed with a heating pad to maintain body temperature. A 5 min CT scan was obtained for localization with voltage setting at 60 kV and current at 615 µA followed by a static emission scan using an ultrahigh-resolution multipinhole rate-mouse (1 mm pinhole size) collimator. Data were acquired in list mode, reconstructed using the U-SPECT II software, and co-registered for alignment. SPECT images were reconstructed using maximum likelihood expectation maximization (3 iterations), pixel-based ordered subset expectation (16 subsets), and a postprocessing filter (Gaussian blurring) of 0.5 mm centered at photopeaks 171 and 245 keV with a 20% window width. Imaging data sets were decay corrected to injection time, and converted to DICOM data for visualization in the Inveon Research Workplace (Siemens Medical Solutions USA, Inc.). For biodistribution studies, at 6 days post injection, mice were sacrificed by the inhalation of isoflurane followed by CO_2_, blood was withdrawn by cardiac puncture, and tissues of interest including fat, uterus, ovaries, intestine, spleen, liver, pancreas, stomach, adrenal glands, kidney, lungs, heart, SKOV-3 tumor, muscle, bone, and brain were harvested, washed in PBS, dried, and weighed. Activity of each sample was measured by a calibrated γ counter (PerkinElmer, Wizard 2 2480) with decay correction. The activity uptake was expressed as percentage of injected dose per gram of tissue (% ID/g).

#### Ex vivo autoradiography

Half of the SKOV-3 tumors were harvested and frozen in a cryoprotective gel (Tissue-Tek optimal cutting temperature compound, Sakura) using dry ice for autoradiography and histology. They were then cut using the NX70 cryostat (Thermo Fisher Scientific) set at a temperature of -15 ºC, mounted on Superfrost Plus Gold slides and fixed in methanol for 5 min at room temperature. For autoradiography, a phosphor screen was applied on 14 μm-thick sections and the resulting signals were acquired with the PhosphoImager (GE Typhoon FLA 9500). The consecutive sections of 8 µm were stained with Hematoxylin and Eosin (H&E) following supplier recommendations (Leica Biosystem).

## Supplementary Information

**Additional file 1.** Supplementary Methods for synthesis and characterization of failed routes. **Scheme S1.** Initial attempt to synthesize the scaffold from 4,4′-difluoro-BODIPY fluorophore 1. **Figs. S1–S32.** 1H and 13C NMR spectra. **Figs. S33–S34.** HPLC chromatograms. **Fig. S35–S36.** Excitation and emission spectra. **Figs. S37–S39.** MALDI-TOF MS/MS. **Figs. S40–S46.** Radio-TLC and radio-HPLC chromatograms. **Table S1.** Biodistribution data.

## Data Availability

The datasets used and/or analyzed during the current study are available from the corresponding author on reasonable request.

## List of symbols

α: Alpha
β^+^: Positron
β^−^: Beta/negatron
γ: Gamma
BODIPY: Boron-dipyrromethane; 4,4-difluoro-4-bora-3a,4a-diaza-*s*-indacene
CT: Computed tomography
DFO: Deferoxamine
DMSO: Dimethylsulfoxide
DOTA: 1,4,7,10-Tetraazacyclododecane-1,4,7,10-tetraacetic acid
EC: Electron capture
HER2: Human epidermal growth factor receptor-2
IEDDA: Inverse electron demand Diels–Alder
IR: Infrared
mAb: Monoclonal antibody
MAE: Meitner-Auger electron
MALDI-TOF: Matrix-assisted laser desorption/ionization time-of-flight
MP: Mobile phase
MS: Mass spectrometry
NHS: N-hydroxysuccinimide
NMR: Nuclear magnetic resonance
PBS: Phosphate buffered saline
PET: Positron emission tomography
PLQY: Photoluminescent quantum yield
PSMA: Prostate specific membrane antigen
RCY: Radiochemical yield
RP-HPLC: Reverse phase high performance liquid chromatography
SPECT: Single-photon emission computed tomography
TAT: Targeted alpha therapy
TCO: Trans-cyclooctene
TFA: Trifluoroacetic acid
TLC: Thin layer chromatography
TMS: Tetramethylsilane
Tz: Tetrazine
UV: Ultra violet

## References

[CR1] Alnajjar MA, Bartelmeß J, Hein R, Ashokkumar P, Nilam M, Nau WM, Rurack K, Hennig A (2018). Rational design of boron-dipyrromethene (bodipy) reporter dyes for cucurbit[7]uril. Beilstein J Org Chem.

[CR2] Altai M, Membreno R, Cook B, Tolmachev V, Zeglis BM (2017). Pretargeted imaging and therapy. J Nucl Med.

[CR3] Ariztia J, Solmont K, Moïse NP, Specklin S, Heck MP, Lamandé-Langle S, Kuhnast B (2022). PET/fluorescence imaging: an overview of the chemical strategies to build dual imaging tools. Biocon Chem.

[CR4] Bernhard C, Goze C, Rousselin Y, Denat F (2010). First bodipy–DOTA derivatives as probes for bimodal imaging. Chem Commun.

[CR5] Bernhard C, Moreau M, Lhenry D, Goze C, Boschetti F, Rousselin Y, Brunotte F, Denat F (2012). DOTAGA-anhydride: a valuable building block for the preparation of DOTA-like chelating agents. Chemistry.

[CR6] Blower PJ (2015). A Nuclear chocolate box: the periodic table of nuclear medicine. Dalt Trans.

[CR7] Brellier M, Duportail G, Baati R (2010). Convenient synthesis of water-soluble nitrilotriacetic acid (NTA) BODIPY Dyes. Tetrahedron Lett.

[CR8] Canovas C, Moreau M, Vrigneaud JM, Bellaye PS, Collin B, Denat F, Goncalves V (2019). Modular assembly of multimodal imaging agents through an inverse electron demand diels-alder reaction. Bioconjug Chem.

[CR9] Comba P, Jermilova U, Orvig C, Patrick BO, Ramogida CF, Rück K, Schneider C, Starke M (2017). Octadentate picolinic acid-based bispidine ligand for radiometal ions. Chem A Eur J.

[CR10] Compton BJ, Farrand KJ, Tang C, Osmond TL, Speir M, Authier-Hall A, Wang J, Ferguson PM, Chan STS, Anderson RJ, Cooney TR, Hayman CM, Williams GM, Brimble MA, Brooks CR, Yong L-K, Metelitsa LS, Zajonc DM, Godfrey DI, Gasser O, Weinkove R, Painter GF, Hermans IF (2019). Enhancing T Cell responses and tumour immunity by vaccination with peptides conjugated to a weak NKT cell agonist. Org Biomol Chem.

[CR11] Crawford SM, Thompson A (2010). Conversion of 4,4-Difluoro-4-Bora-3a,4a-Diaza- s -indacenes ( F -BODIPYs) to dipyrrins with a microwave-promoted deprotection strategy. Org Lett.

[CR12] Deken MM, Bos DL, Tummers WSFJ, March TL, van de Velde CJH, Rijpkema M, Vahrmeijer AL (2019). Multimodal image-guided surgery of HER2-positive breast cancer using [111In]In-DTPA-trastuzumab-IRDye800CW in an orthotopic breast tumor model. EJNMMI Res.

[CR13] He S, Song J, Qu J, Cheng Z (2018). Crucial Breakthrough of second near-infrared biological window fluorophores: design and synthesis toward multimodal imaging and theranostics. Chem Soc Rev.

[CR14] Hensbergen AW, van Willigen DM, van Beurden F, van Leeuwen PJ, Buckle T, Schottelius M, Maurer T, Wester H-J, van Leeuwen FWB (2020). Image-guided surgery: are we getting the most out of small-molecule prostate-specific-membrane-antigen-targeted tracers?. Bioconjug Chem.

[CR15] Ieda N, Hotta Y, Miyata N, Kimura K, Nakagawa H (2014). Photomanipulation of vasodilation with a blue-light-controllable nitric oxide releaser. J Am Chem Soc.

[CR16] Kang CM, Kim H, Koo HJ, Park JW, An GIl, Choi JY, Lee KH, Kim BT, Choe YS (2016). Catabolism of 64Cu and Cy5.5-labeled human serum albumin in a tumor xenograft model. Amino Acids.

[CR17] Keinänen O, Brennan JM, Membreno R, Fung K, Gangangari K, Dayts EJ, Williams CJ, Zeglis BM (2019). Dual radionuclide theranostic pretargeting. Mol Pharm.

[CR18] Keinänen O, Fung K, Brennan JM, Zia N, Harris M, van Dam E, Biggin C, Hedt A, Stoner J, Donnelly PS, Lewis JS, Zeglis BM (2020). Harnessing 64Cu/67Cu for a theranostic approach to pretargeted radioimmunotherapy. Proc Natl Acad Sci U S A.

[CR19] Kostelnik TI, Orvig C (2019). Radioactive main group and rare earth metals for imaging and therapy. Chem Rev.

[CR20] Lhenry D, Larrouy M, Bernhard C, Goncalves V, Raguin O, Provent P, Moreau M, Collin B, Oudot A, Vrigneaud J-M, Brunotte F, Goze C, Denat F (2015). BODIPY: a highly versatile platform for the design of bimodal imaging probes. Chem - A Eur J.

[CR21] Liras M, Pintado-Sierra M, Iglesias M, Sánchez F (2016). A Deprotection strategy of a bodipy conjugated porous polymer to obtain a heterogeneous (dipyrrin)(bipyridine)ruthenium( <scp>ii</scp> ) visible light photocatalyst. J Mater Chem A.

[CR22] Liu L (2015). Antibody glycosylation and its impact on the pharmacokinetics and pharmacodynamics of monoclonal antibodies and Fc-fusion proteins. J Pharm Sci.

[CR23] Lub-de Hooge MN, Kosterink JGW, Perik PJ, Nijnuis H, Tran L, Bart J, Suurmeijer AJH, de Jong S, Jager PL, de Vries EGE (2004). Preclinical characterisation of 111 In-DTPA-trastuzumab. Br J Pharmacol.

[CR24] Lundrigan T, Cameron TS, Thompson A (2014). Activation and deprotection of F-BODIPYs using boron trihalides. Chem Commun.

[CR25] Maindron N, Ipuy M, Bernhard C, Lhenry D, Moreau M, Carme S, Oudot A, Collin B, Vrigneaud J-M, Provent P, Brunotte F, Denat F, Goze C (2016). Near-infrared-emitting BODIPY-TrisDOTA 111 in as a monomolecular multifunctional imaging probe: from synthesis to in vivo investigations. Chem - A Eur J.

[CR26] McLarty K, Cornelissen B, Scollard DA, Done SJ, Chun K, Reilly RM (2009). Associations between the uptake of 111In-DTPA-trastuzumab, HER2 density and response to trastuzumab (herceptin) in athymic mice bearing subcutaneous human tumour xenografts. Eur J Nucl Med Mol Imaging.

[CR27] Meimetis LG, Boros E, Carlson JC, Ran C, Caravan P, Weissleder R (2016). Bioorthogonal fluorophore linked DFO—technology enabling facile chelator quantification and multimodal imaging of antibodies. Bioconjug Chem.

[CR28] Membreno R, Cook BE, Zeglis BM (2019). Pretargeted radioimmunotherapy based on the inverse electron demand diels-alder reaction. J vis Exp.

[CR29] Milenic DE, Wong KJ, Baidoo KE, Nayak TK, Regino CAS, Garmestani K, Brechbiel MW (2010). Targeting HER2. Mabs.

[CR30] Morgenstern A, Apostolidis C, Kratochwil C, Sathekge M, Krolicki L, Bruchertseifer F (2018). An overview of targeted alpha therapy with ^225^actinium and ^213^bismuth. Curr Radiopharm.

[CR31] Morgenstern A, Apostolidis C, Bruchertseifer F (2020). Supply and clinical application of actinium-225 and bismuth-213. Semin Nucl Med.

[CR32] Nguyen AL, Wang M, Bobadova-Parvanova P, Do Q, Zhou Z, Fronczek FR, Smith KM, Vicente MGH (2016). Synthesis and properties of B -cyano-BODIPYs. J Porphyr Phthalocyanines.

[CR33] Oliveira BL, Guo Z, Bernardes GJL (2017). Inverse electron demand diels-alder reactions in chemical biology. Chem Soc Rev.

[CR34] Patra M, Zarschler K, Pietzsch HJ, Stephan H, Gasser G (2016). New insights into the pretargeting approach to image and treat tumours. Chem Soc Rev.

[CR35] Poty S, Membreno R, Glaser JM, Ragupathi A, Scholz WW, Zeglis BM, Lewis JS (2018). The Inverse electron-demand diels-alder reaction as a new methodology for the synthesis of 225 Ac-labelled radioimmunoconjugates. Chem Commun.

[CR37] Poty S, Carter LM, Mandleywala K, Membreno R, Abdel-Atti D, Ragupathi A, Scholz WW, Zeglis BM, Lewis JS (2019). Leveraging bioorthogonal click chemistry to improve 225Ac-radioimmunotherapy of pancreatic ductal adenocarcinoma. Clin Cancer Res.

[CR38] Price E, Zeglis B, Cawthray JF, Ramogida CF, Ramos N, Lewis JS, Adam MJ, Orvig C (2013). H4octapa-trastuzumab: versatile acyclic chelate system for 111In and 177Lu imaging and therapy. J Am Chem Soc.

[CR39] Price EW, Zeglis BM, Lewis JS, Adam MJ, Orvig C (2014). H 6 Phospa-trastuzumab: bifunctional methylenephosphonate-based chelator with 89 Zr, 111 In and 177 Lu. Dalt Trans.

[CR40] Ramogida CF, Cawthray JF, Boros E, Ferreira CL, Patrick BO, Adam MJ, Orvig C (2015). H2CHX dedpa and H4CHX octapa-chiral acyclic chelating ligands for 67/68Ga and 111In radiopharmaceuticals. Inorg Chem.

[CR41] Ramogida CF, Robertson AKH, Jermilova U, Zhang C, Yang H, Kunz P, Lassen J, Bratanovic I, Brown V, Southcott L, Rodríguez-Rodríguez C, Radchenko V, Bénard F, Orvig C, Schaffer P (2019). Evaluation of polydentate picolinic acid chelating ligands and an α-melanocyte-stimulating hormone derivative for targeted alpha therapy using isol-produced 225Ac. EJNMMI Radiopharm Chem.

[CR42] Robertson AKH, McNeil BL, Yang H, Gendron D, Perron R, Radchenko V, Zeisler S, Causey P, Schaffer P (2020). 232 Th-spallation-produced 225 Ac with reduced 227 Ac content. Inorg Chem.

[CR43] Rosenthal EL, Warram JM, de Boer E, Chung TK, Korb ML, Brandwein-Gensler M, Strong TV, Schmalbach CE, Morlandt AB, Agarwal G, Hartman YE, Carroll WR, Richman JS, Clemons LK, Nabell LM, Zinn KR (2015). Safety and tumor specificity of cetuximab-irdye800 for surgical navigation in head and neck cancer. Clin Cancer Res.

[CR44] Rumyantsev EV, Alyoshin SN, Marfin YS (2013). Kinetic study of bodipy resistance to acids and alkalis: stability ranges in aqueous and non-aqueous solutions. Inorganica Chim Acta.

[CR45] Seibold U, Wängler B, Wängler C (2017). Rational design, development, and stability assessment of a macrocyclic four-hydroxamate-bearing bifunctional chelating agent for 89 Zr. ChemMedChem.

[CR46] Smithen DA, Baker AEG, Offman M, Crawford SM, Cameron TS, Thompson A (2012). Use of F -BODIPYs as a protection strategy for dipyrrins: optimization of BF 2 removal. J Org Chem.

[CR47] Spreckelmeyer S, Ramogida CF, Rousseau J, Arane K, Bratanovic I, Colpo N, Jermilova U, Dias GM, Dude I, de Jaraquemada-Peláez MG, Bénard F, Schaffer P, Orvig C (2017). P-NO2-Bn-H4neunpa and H4neunpa-trastuzumab: bifunctional chelator for radiometalpharmaceuticals and 111In immuno-SPECT imaging. Bioconjug Chem.

[CR48] Sui B, Liu X, Wang M, Belfield KD (2016). A highly selective fluorescence turn-on sensor for extracellular calcium ion detection. Chem - A Eur J.

[CR49] Summers GH, Lowe G, Lefebvre J-F, Ngwerume S, Bräutigam M, Dietzek B, Camp JE, Gibson EA (2017). Resonance raman study of new pyrrole-anchoring dyes for NiO-sensitized solar cells. ChemPhysChem.

[CR50] Thiele NA, Brown V, Kelly JM, Amor-Coarasa A, Jermilova U, MacMillan SN, Nikolopoulou A, Ponnala S, Ramogida CF, Robertson AKH, Rodríguez-Rodríguez C, Schaffer P, Williams C, Babich JW, Radchenko V, Wilson JJ (2017). An Eighteen-membered macrocyclic ligand for actinium-225 targeted alpha therapy. Angew Chemie Int Ed.

[CR51] Thierer LM, Tomson NC (2017). The actinium aqua ion: a century in the making. ACS Cent Sci.

[CR52] Tsai WK, Zettlitz KA, Tavaré R, Kobayashi N, Reiter RE, Wu AM (2018). Dual-modality immunopet/fluorescence imaging of prostate cancer with an anti-PSCA Cys-minibody. Theranostics.

[CR53] Tsuji G, Hattori T, Kato M, Hakamata W, Inoue H, Naito M, Kurihara M, Demizu Y, Shoda T (2018). Design and synthesis of cell-permeable fluorescent nitrilotriacetic acid derivatives. Bioorg Med Chem.

[CR54] Uriel C, Permingeat C, Ventura J, Avellanal-Zaballa E, Bañuelos J, García-Moreno I, Gómez AM, Lopez JC (2020). BODIPYs as chemically stable fluorescent tags for synthetic glycosylation strategies towards fluorescently labeled saccharides. Chem A Eur J.

[CR55] Urieta J, Maroto BL, Moreno F, Agarrabeitia AR, Ortiz MJ, de la Moya S (2015). Preparation of dipyrrins from F-BODIPYs by treatment with methanesulfonic acids. RSC Adv.

[CR56] van Leeuwen FWB, Schottelius M, Brouwer OR, Vidal-Sicart S, Achilefu S, Klode J, Wester H-J, Buckle T (2020). Trending: radioactive and fluorescent bimodal/hybrid tracers as multiplexing solutions for surgical guidance. J Nucl Med.

[CR57] Wang M, Vicente MGH, Mason D, Bobadova-Parvanova P (2018). Stability of a series of BODIPYs in acidic conditions: an experimental and computational study into the role of the substituents at boron. ACS Omega.

[CR58] Wu H, Devaraj NK (2018). Advances in tetrazine bioorthogonal chemistry driven by the synthesis of novel tetrazines and dienophiles. Acc Chem Res.

[CR59] Yu M, Wong JKH, Tang C, Turner P, Todd MH, Rutledge PJ (2015). Efficient deprotection of F -BODIPY derivatives: removal of BF 2 using brønsted acids. Beilstein J Org Chem.

[CR60] Zhao N, Xuan S, Byrd B, Fronczek FR, Smith KM, Vicente MGH (2016). Synthesis and regioselective functionalization of perhalogenated BODIPYs. Org Biomol Chem.

[CR61] Zhou Q, van den Berg NS, Rosenthal EL, Iv M, Zhang M, Vega Leonel JCM, Walters S, Nishio N, Granucci M, Raymundo R, Yi G, Vogel H, Cayrol R, Lee Y-J, Lu G, Hom M, Kang W, Hayden Gephart M, Recht L, Nagpal S, Thomas R, Patel C, Grant GA, Li G (2021). EGFR-targeted intraoperative fluorescence imaging detects high-grade glioma with panitumumab-IRDye800 in a Phase 1 Clinical Trial. Theranostics.

