# Supplementary material for: Development of a multi faceted platform containing a tetrazine, fluorophore and chelator: synthesis, characterization, radiolabeling, and immuno-SPECT imaging
Source: EJNMMI Radiopharm Chem. 2022 Jun 6;7:12. doi: 10.1186/s41181-022-00164-1 (PMC9170845; doi:10.1186/s41181-022-00164-1)
Supplement: Supplementary file 1 — Additional file 1. Supplementary Methods for synthesis and characterization of failed routes. Scheme S1. Initial attempt to synthesize the scaffold from 4,4′-difluoro-BODIPY fluorophore 1. Figs. S1–S32. 1H and 13C NMR spectra. Figs. S33–S34. HPLC chromatograms. Fig. S35–S36. Excitation and emission spectra. Figs. S37–S39. MALDI-TOF MS/MS. Figs. S40–S46. Radio-TLC and radio-HPLC chromatograms. Table S1. Biodistribution data. [file 41181_2022_164_MOESM1_ESM.pdf]

Supporting Information

Supplementary Material

Development of a multi faceted platform containing a tetrazine, fluorophore and chelator: synthesis, characterization, radiolabeling, and immuno-SPECT imaging

Anthony W. McDonagh[1], Brooke McNeil[1,2], Julie Rousseau[3], Ryan J. Roberts[1], Helen Merkens[3], Hua Yang[2], François Bénard[3], and Caterina F. Ramogida[1,2]*

[1]Department of Chemistry, Simon Fraser University, Burnaby, BC, V5A 1S6 Canada

[2]Life Sciences Division, TRIUMF, Vancouver, BC V6T 2A3 Canada

[3]Department of Molecular Oncology, BC Cancer Agency, Vancouver, BC V5Z 1L3 Canada

* Corresponding author: Caterina F. Ramogida, Department of Chemistry, Simon Fraser University, Life Sciences Division, TRIUMF, Canada, cfr@sfu.ca

Table of Contents

1. SUPPLEMENTARY METHODS 3

1.1 Synthesis and Characterization of Compounds from Failed Routes 3

1. SUPPLEMENTAL TABLES, SCHEMES, & FIGURES 8

1. SUPPLEMENTARY METHODS

1.1 Synthesis and Characterization of Compounds from Failed Routes

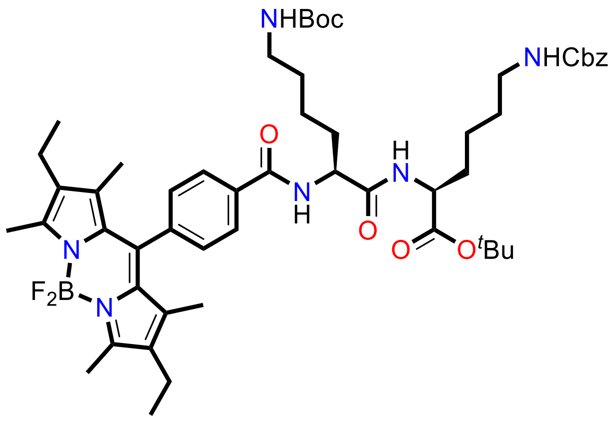

***tert*-butyl *N*[6]-((benzyloxy)carbonyl)-*N*[2]-(*N*[6]-(tert-butoxycarbonyl)-*N*[2]-(4-(4,4-difluoro-2,6-diethyl-1,3,5,7-tetramethyl-4-bora-3a,4a-diaza-*s*-indacen-8-yl)benzoyl)-*L*-lysyl)-*L*-lysinate (3).** A solution of the amine **2** (425 mg, 0.75 mmol) and triethylamine (0.21 mL, 1.51 mmol) in dry CH_2Cl_2 (7 mL) was added under argon to a solution of the succinimide **1** (510 mg, 0.98 mmol) in dry CH_2Cl_2 (10 mL). The reaction mixture was stirred in the dark at room temperature for 3 days. The solvent was removed under reduced pressure and flash chromatography of the residue (CH_2Cl_2-CH_3CN 4:1 – 2:1) gave **3** (522 mg, 72%) as a red-orange solid; R_f 0.24 (CH_2Cl_2-CH_3CN 2:1); IR (film) cm^{-1}: 2960, 2928, 2864, 2471, 2408, 1692, 1633, 1539, 1423, 1188, 1155, 977, 732, 697, 534; ^1H NMR (MeOD, 500 MHz) δ 8.04 (2H, d, *J* 8.3, Ar-H), 7.37 (5H, d, *J* 8.3, Ar-H), 7.33 – 7.21 (5H, m, $OCH_2C_6H_5$), 5.10 – 5.01 (2H, m, $OCH_2C_6H_5$), 4.59 (1H, dd, *J* 8.8, 5.8, NHC*H*), 4.31 (1H, dd, *J* 9.0, 4.9, NHC*H*), 3.18 – 3.03 (4H, m, CH_2NHBoc & CH_2NHCbz), 2.48 (6H, s, CH_3 x 2), 2.34 (4H, q, *J* 7.5, CH_2CH_3), 1.99 – 1.81 (3H, m, CH_aH_b & $CH_{a'}H_{b'}$), 1.76 – 1.67 (1H, m, $CH_{a'}H_{b'}$), 1.59 – 1.39 (26H, m, each CH_2 x 4 & *t*-Bu x 2), 1.30 (6H, s, CH_3 x 2),

0.98 (6H, t, *J* 7.5, CH_2CH_3); ^{13}C NMR (MeOD, 125 MHz) δ 174.5, 172.8, 169.4, 158.9, 158.6 (each C=O), 155.2, 140.8, 140.5, 139.5, 138.4, 135.9, 134.3, 131.6, 129.9, 129.6, 129.4, 128.9, 128.8 (each Ar-C), 82.8 (*C*$(CH_3)_3$), 79.9 (*C*$(CH_3)_3$), 67.3 (O*C*$H_2C_6H_5$), 55.5 (NH*C*H), 54.4 (NH*C*H), 41.6, 41.1 (*C*H_2NHBoc & *C*H_2NHCbz), 32.7, 32.2, 30.7, 30.3 (each CH_2), 28.8 (C$(CH_3)_3$), 28.3 (C(*C*$H_3)_3$), 24.5, 24.0 (each CH_2), 17.8 (*C*H_2CH_3), 15.0 (CH_2*C*H_3), 12.6 (CH_3), 12.2 (CH_3); ESI-HRMS calcd. $C_{53}H_{74}BF_2N_6O_8$, 971.5629 found m/z 971.5640 $[M+H]^+$.

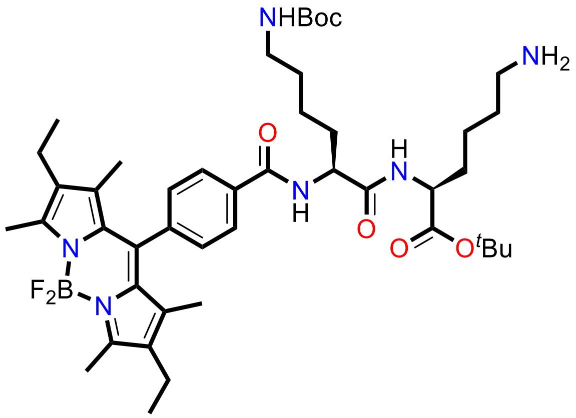

***tert*-butyl *N*^{2}-(*N*^{6}-(tert-butoxycarbonyl)-*N*^{2}-(4-(4,4-difluoro-2,6-diethyl-1,3,5,7-tetramethyl-4-bora-3a,4a-diaza-*s*-indacen-8-yl)benzoyl)-*L*-lysyl)-*L*-lysinate (4).** To a stirred solution of **3** (100 mg, 103 µmol) in MeOH (4 mL) was added 10% palladium on carbon (28 mg). The reaction flask was stoppered and flushed with argon for 1 min followed by hydrogen for a further 1 min. The mixture was stirred at room temperature in the dark under hydrogen (balloon) for 2 h, filtered through Celite and the solvent was removed under reduced pressure to give **4** (86 mg, 100%) as a red waxy solid; ^{1}H NMR (MeOD, 400 MHz) δ 8.06 (2H, d, *J* 8.3, Ar-H), 7.47 (2H, d, *J* 8.3, Ar-H), 4.59 (1H, dd, *J* 8.8, 5.6, NHC*H*), 4.33 (1H, dd, *J* 9.2, 4.9, NHC*H*), 3.11 – 3.05 (2H, m, CH_2), 2.69 (2H, t, *J* 6.7, CH_2), 2.48 (6H, s, CH_3 x 2), 2.35 (4H, q, *J* 7.4, C*H*$_2CH_3$), 1.98 – 1.82 (3H, m, each CH_2 & CH), 1.73 (1H, m, CH), 1.60 – 1.40 (26H, m, each CH_2 & *t*-Bu), 1.33 (6H, s, CH_3 x 2), 0.99 (6H, t, *J* 7.5, CH_2CH_3); ^{13}C NMR (MeOD, 100 MHz) δ 174.7, 172.7, 169.4, 158.3, 155.2 (each C=O), 140.8, 140.5, 139.4, 135.9, 134.3, 131.6, 129.9, 129.6 (each Ar-C), 82.8 (*C*$(CH_3)_3$), 79.8 (*C*$(CH_3)_3$), 55.6 (NH*C*H), 54.2 (NH*C*H), 41.4, 41.1 (*C*H_2NHBoc &

CH_2NH_2), 32.5, 32.2, 30.6 (each CH_2), 28.8 ($C(CH_3)_3$), 28.3 ($C(CH_3)_3$), 24.5, 24.0 (each CH_2), 17.8 (*C*H_2CH_3), 15.0 (CH_2*C*H_3), 12.6 (CH_3), 12.2 (CH_3); ESI-HRMS calcd. $C_{45}H_{68}BF_2N_6O_6$, 837.5261 found m/z 837.5243 $[M+H]^+$

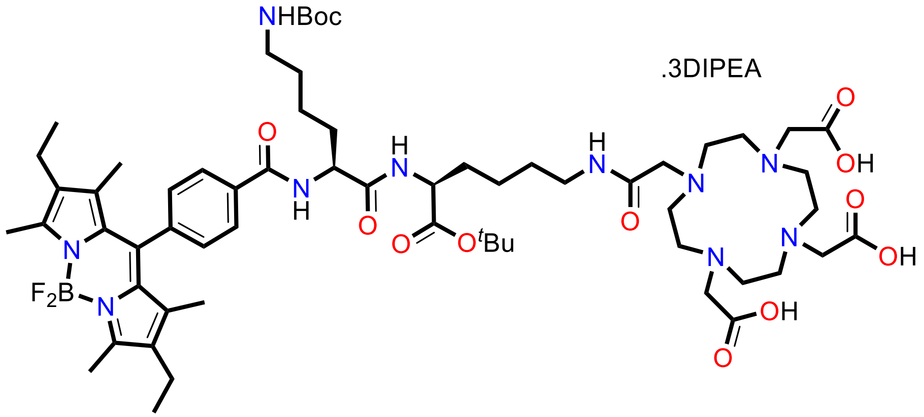

2,2',2''-(10-((10S,13S)-13-(tert-butoxycarbonyl)-10-(4-(2,8-diethyl-5,5-difluoro-1,3,7,9-tetramethyl-5H-4l4,5l4-dipyrrolo[1,2-c:2',1'-f][1,3,2]diazaborinin-10-yl)benzamido)-2,2-dimethyl-4,11,19-trioxo-3-oxa-5,12,18-triazaicosan-20-yl)-1,4,7,10-tetraazacyclododecane-1,4,7-triyl)triacetic acid (5) To a stirred solution of **4** (6.8 mg, 8.13 µmol) in dry CH_2Cl_2 (0.5 mL) were added DIPEA (14 µL, 81.3 µmol) and DOTA-NHS (6.9 mg, 9.11 µmol). The reaction mixture was stirred at room temperature for two hours and the solvent was removed under a stream of air. The resulting residue was washed with three (2 mL) portions of each Et_2O, hexane and H_2O to give the crude tile compound **13** (13 mg, 99%) at a red-orange solid which was used without further purification; ESI-HRMS calcd. $C_{61}H_{69}BF_2N_{10}O_{13}$, 1225.7219 found m/z 1225.7215 $[M+3H]^+$.

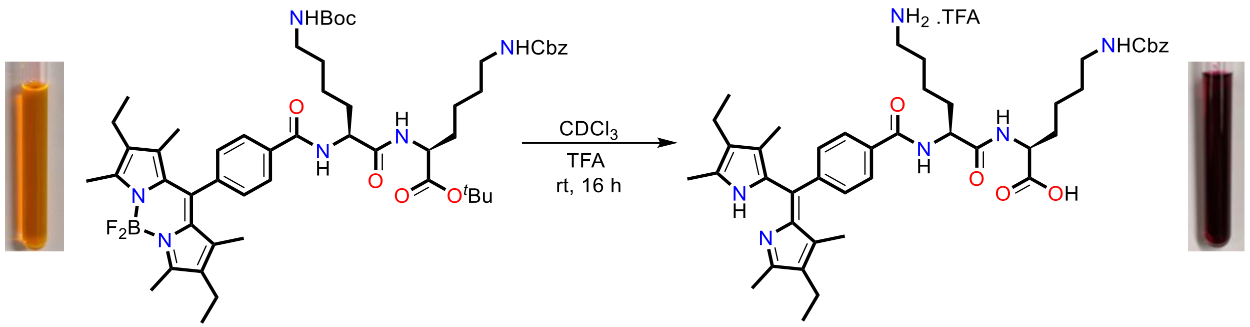

***N*[6]-((benzyloxy)carbonyl)-*N*[2]-((4-((*Z*)-(4-ethyl-3,5-dimethyl-1*H*-pyrrol-2-yl)(4-ethyl-3,5-dimethyl-2*H*-pyrrol-2-ylidene)methyl)benzoyl)-*L*-lysyl)-*L*-lysine (6).** **3** (1.7 mg, 1.75 µmol) was dissolved in $CDCl_3$ (0.4 mL). The resulting solution was transferred to an NMR tube and TFA (80 µL) was added. The reaction was monitored via ^1H NMR, where during which time, the solution colour was observed to change from orange to a deep purple. After leaving the reaction at room temperature overnight ^1H NMR indicated one compound to be present. The reaction was diluted with CH_2Cl_2 (5 mL), transferred to a vial and the solvent was removed under reduced pressure. The resulting residue was washed with Et_2O x 3 to give the dipyrrin **6** (1.4 mg, 93%) as a purple solid; ^1H NMR (MeOD, 500 MHz) δ 8.06 (2H, d, *J* 8.0, Ar-H), 7.47 (2H, d, *J* 8.0, Ar-H), 7.37 – 7.23 (5H, m, Ar-H), 5.09 (1H, d, *J* 12.7, OC*H*HC_6H_5), 5.05 (1H, d, *J* 12.7, OCH*H*C_6H_5), 4.64 (1H, t, *J* 7.2, NHC*H*), 4.47 (1H, dd, *J* 9.5, 4.5, NHC*H*), 3.21 – 3.07 (2H, m, CH_2NH_2), 2.98 (2H, t, *J* 7.6, CH_2NHCBz), 2.55 (6H, q, *J* 7.6, CH_2CH_3), 2.47 (6H, s, CH_3 x 2), 2.05 – 1.86 (4H, m, each CH_2), 1.79 – 1.48 (14H, m, each CH_2 & CH_3), 1.15 (6H, t, *J* 7.6, CH_2CH_3); ESI-HRMS calcd. $C_{44}H_{59}N_6O_6$, 767.4496 found m/z 767.4476 $[M+H]^+$.

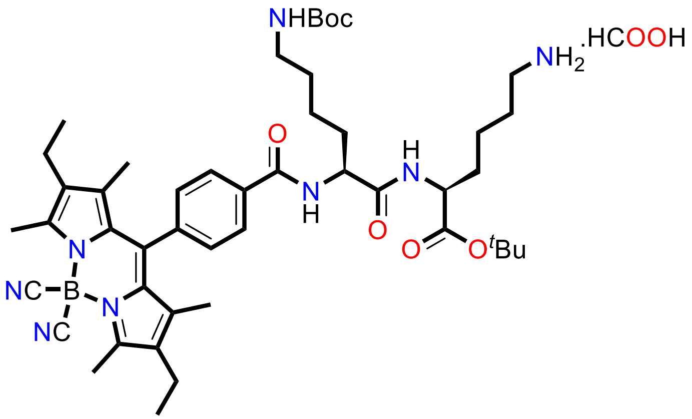

***tert*-butyl *N*[2]-(*N*[6]-(tert-butoxycarbonyl)-*N*[2]-(4-(4,4-dicyano-2,6-diethyl-1,3,5,7-tetramethyl-4-bora-3a,4a-diaza-*s*-indacen-8-yl)benzoyl)-*L*-lysyl)-*L*-lysinium formate (22).** To a stirred solution of **8** (156 mg, 159 µmol) in MeOH (3 mL) and formic acid (52 µL) was added 10% palladium on carbon (64 mg). The reaction flask was stoppered and flushed with argon for 1 min followed by hydrogen for a further 1 min. The mixture was stirred at room temperature in the dark under hydrogen (balloon) for 2 h, filtered through Celite and the solvent was removed under reduced pressure to give **22** (138 mg, 97%) as a red waxy solid. The compound was used without further purification; IR (film) cm^{-1}: 3298, 2967, 2932, 2866, 1541, 1476, 1188, 1152, 980, 734, 545; ^1H NMR (MeOD, 500 MHz) δ 8.55 (1H, s, *H*COO^-), 8.09 (2H, d, *J* 7.9, Ar-H), 7.53 (2H, d, *J* 7.9, Ar-H), 4.52 (1H, dd, *J* 8.9, 5.7, NHC*H*), 4.38 (1H, dd, *J* 9.8, 4.6, NHC*H*), 3.13 – 2.92 (m, 4H, CH_2NHBoc & CH_2NH_2), 2.68 (6H, s, CH_3 x 2), 2.45 (4H, q, *J* 7.5, CH_2CH_3), 1.99 – 1.85 (3H, m, CH_2 & C*H*), 1.79 – 1.64 (3H, m, CH_2 & C*H*), 1.62 – 1.51 (6H, m, each CH_2), 1.48 (9H, s, *t*-Bu), 1.43 (9H, s, *t*-Bu), 1.41 (6H, s, CH_3 x 2), 1.03 (6H, t, *J* 7.5, CH_2CH_3); ^{13}C NMR (MeOD, 125 MHz, CN signals were not observed) δ 174.8, 172.6, 169.5, 155.6 (each C=O), 142.0, 141.5, 139.4, 136.4, 136.2, 136.1, 130.2, 129.9, 129.9 (each Ar-C), 83.0 (*C*$(CH_3)_3$), 79.9 (*C*$(CH_3)_3$), 55.9 (NH*C*H), 53.9 (NH*C*H), 41.1, 40.6 (*C*H_2NHBoc & *C*H_2NH_2), 32.6, 32.0, 30.7 (each CH_2), 28.8($C(CH_3)_3$), 28.3($C(CH_3)_3$), 28.0, 24.6, 23.8 (each CH_2), 17.9 (*C*H_2CH_3), 14.8 (CH_2*C*H_3), 13.6 (CH_3), 12.5(CH_3); ESI-HRMS calcd. $C_{47}H_{68}BN_8O_6$, 851.5349 found m/z 851.5383 $[M+H]^+$.

1. SUPPLEMENTAL TABLES, SCHEMES, & FIGURES

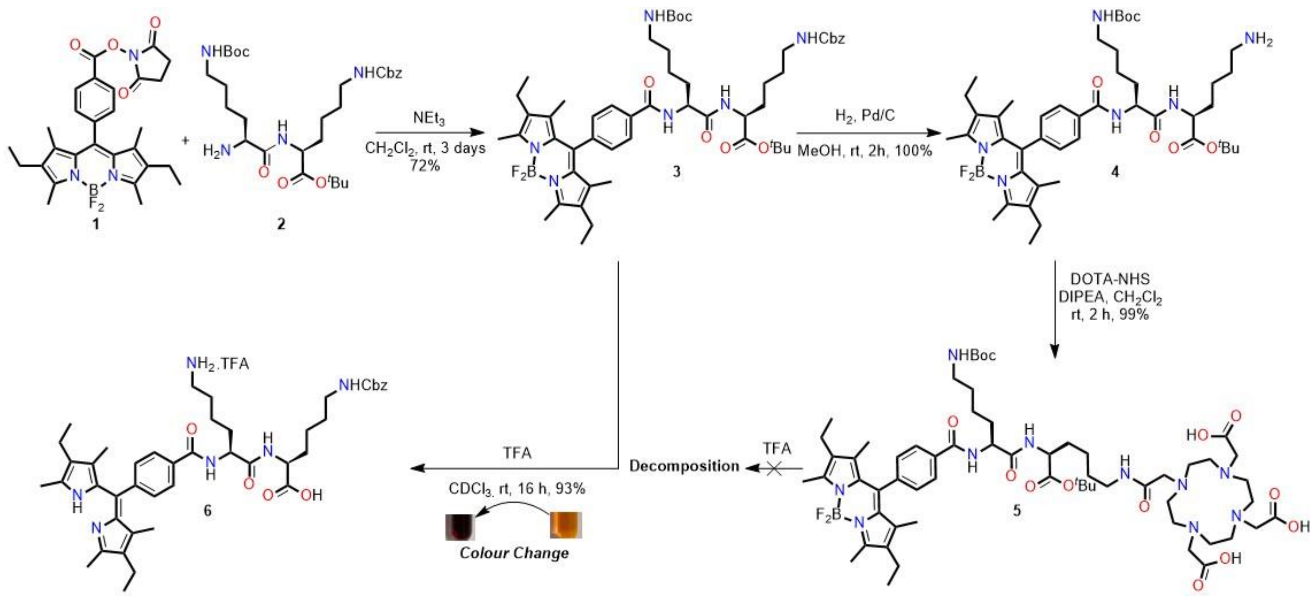

Scheme S1. Initial attempt to synthesize the scaffold from 4,4'-difluoro-BODIPY fluorophore **1**.

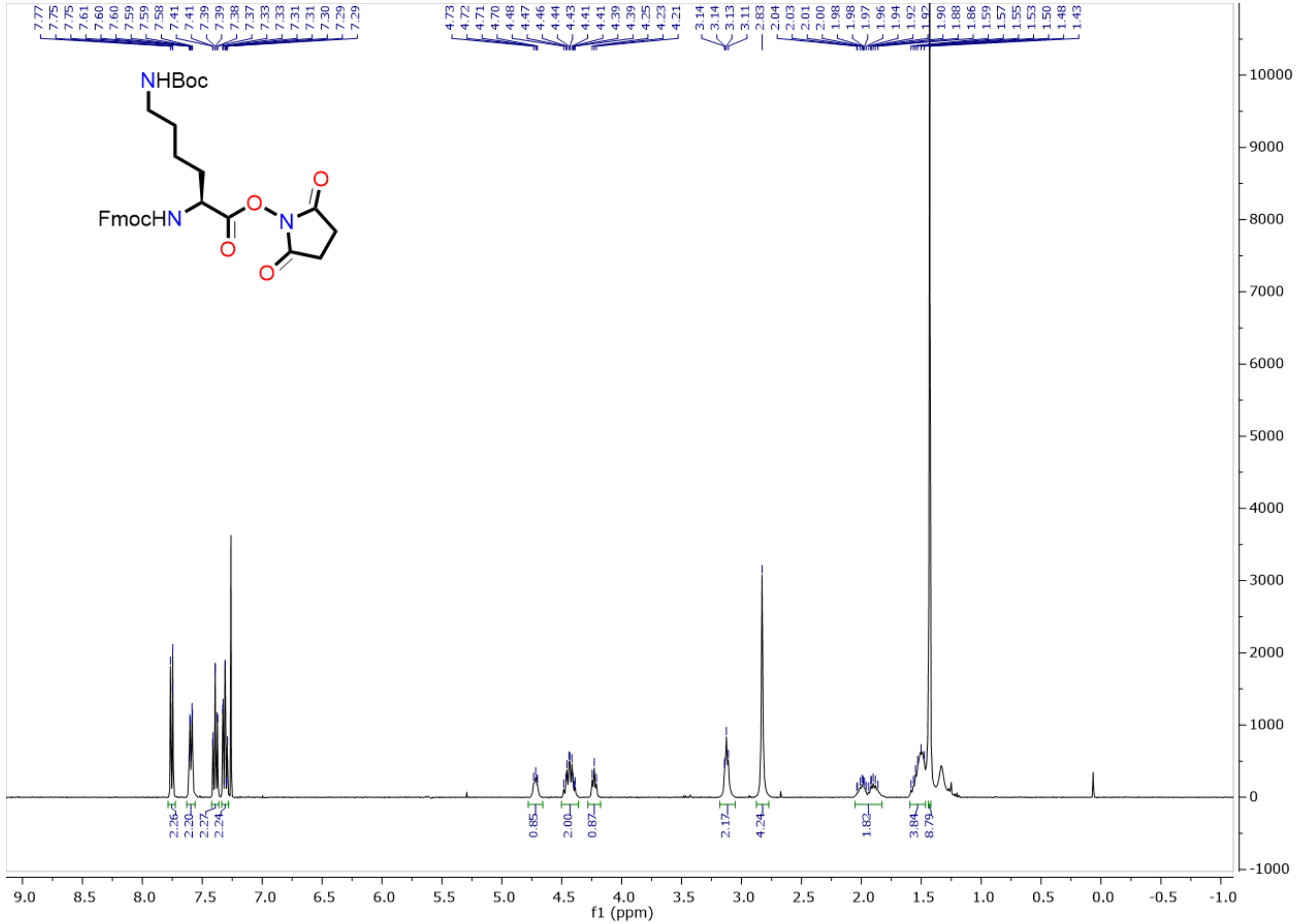

Figure S1. ^{1}H NMR spectrum of **20** (400 MHz, $CDCl_3$-MeOD (1drop), 25°C).

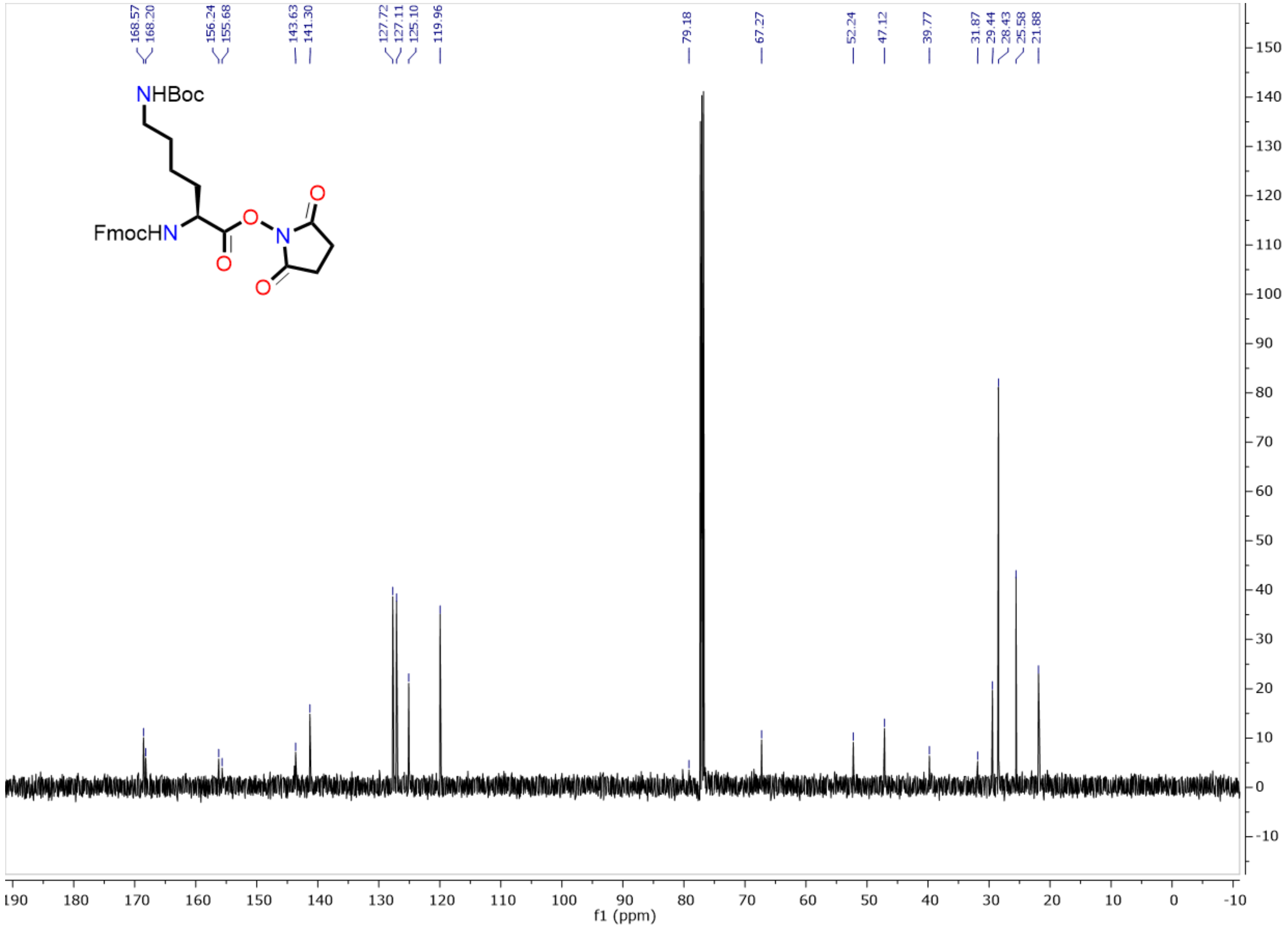

Figure S2. ^{13}C NMR spectrum of **20** (125 MHz, $CDCl_3$, 25°C).

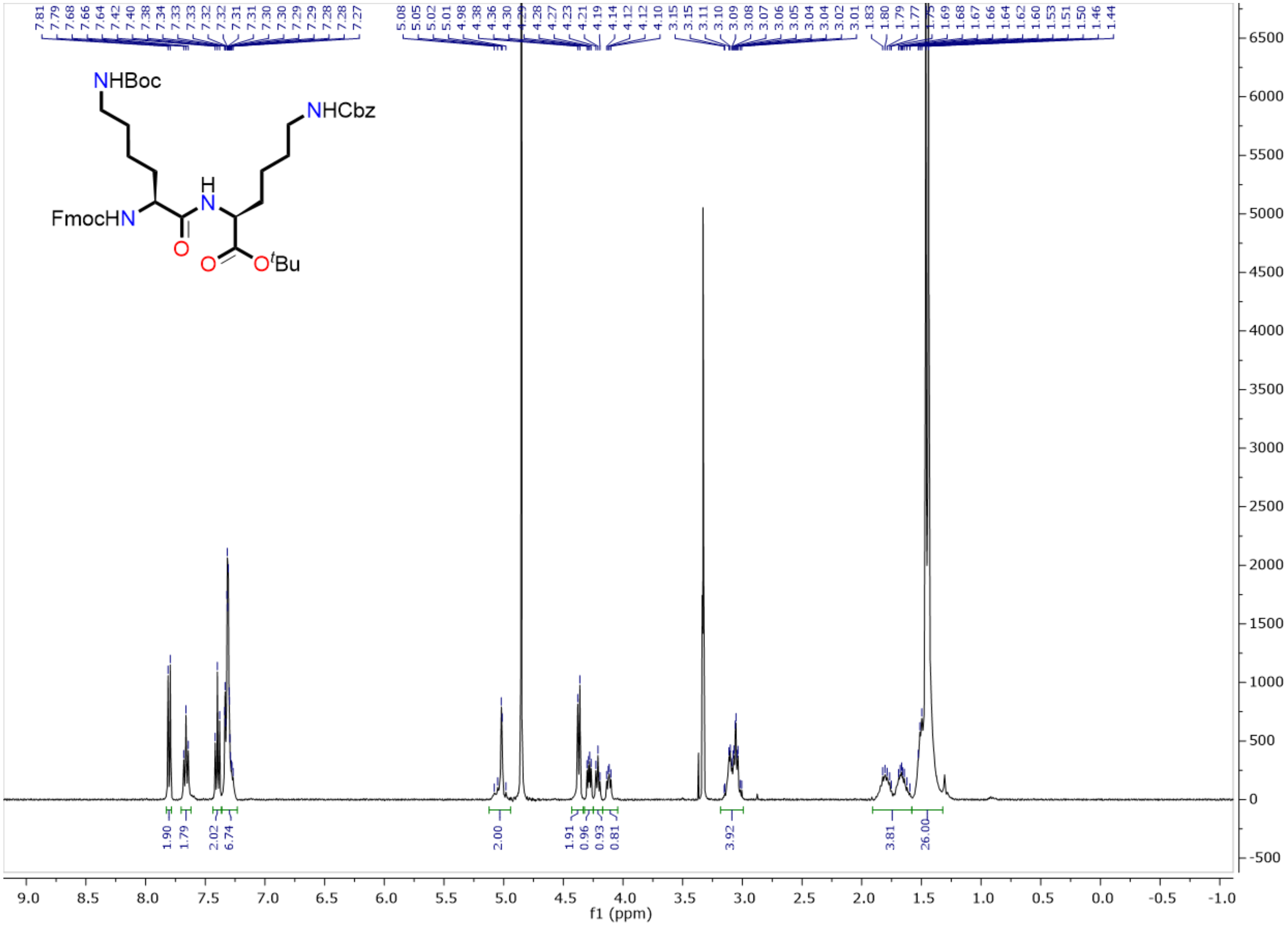

Figure S3. ^{1}H NMR spectrum of **21** (400 MHz, MeOD, 25°C).

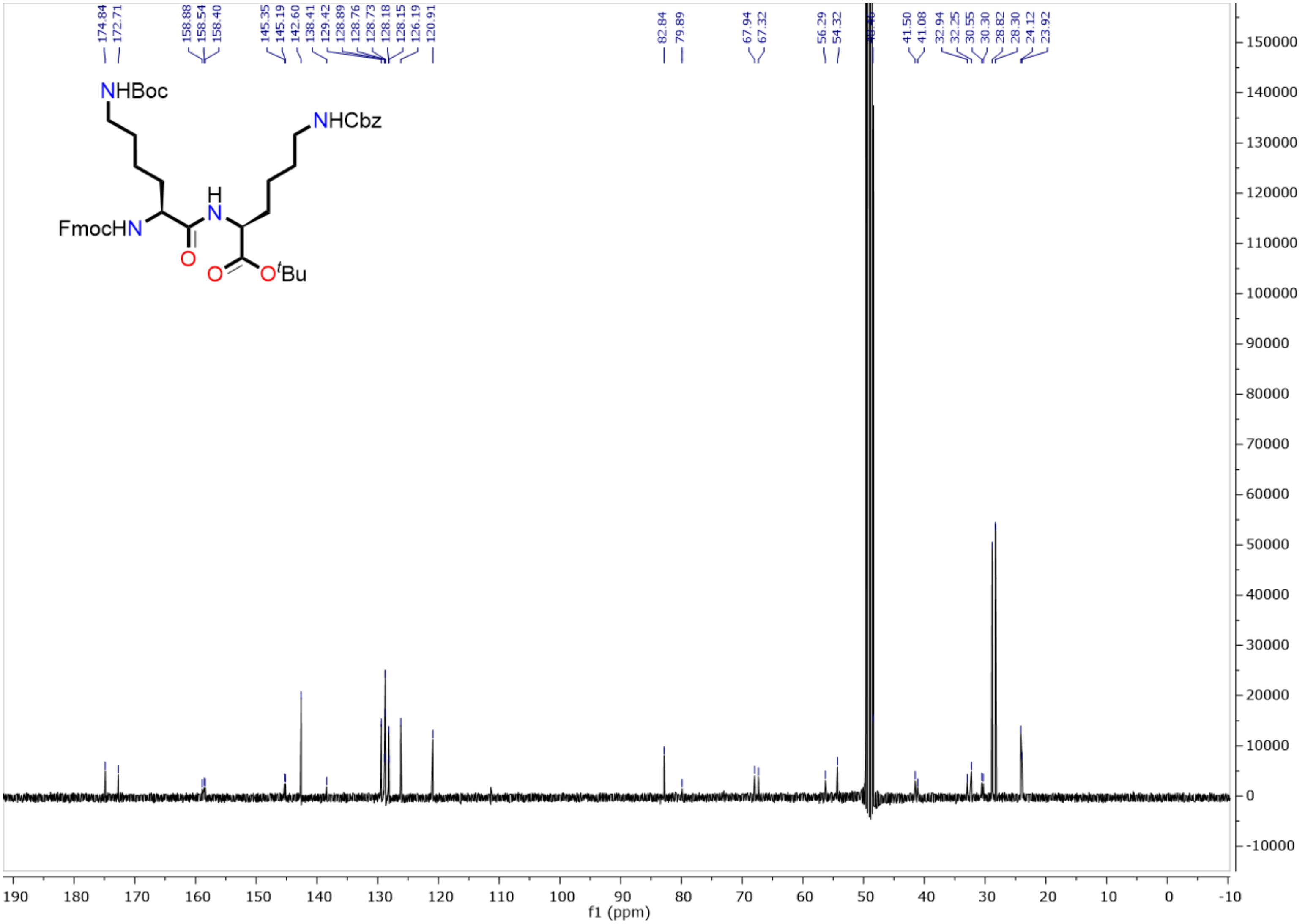

Figure S4. ^{13}C NMR spectrum of **21** (100 MHz, MeOD, 25°C).

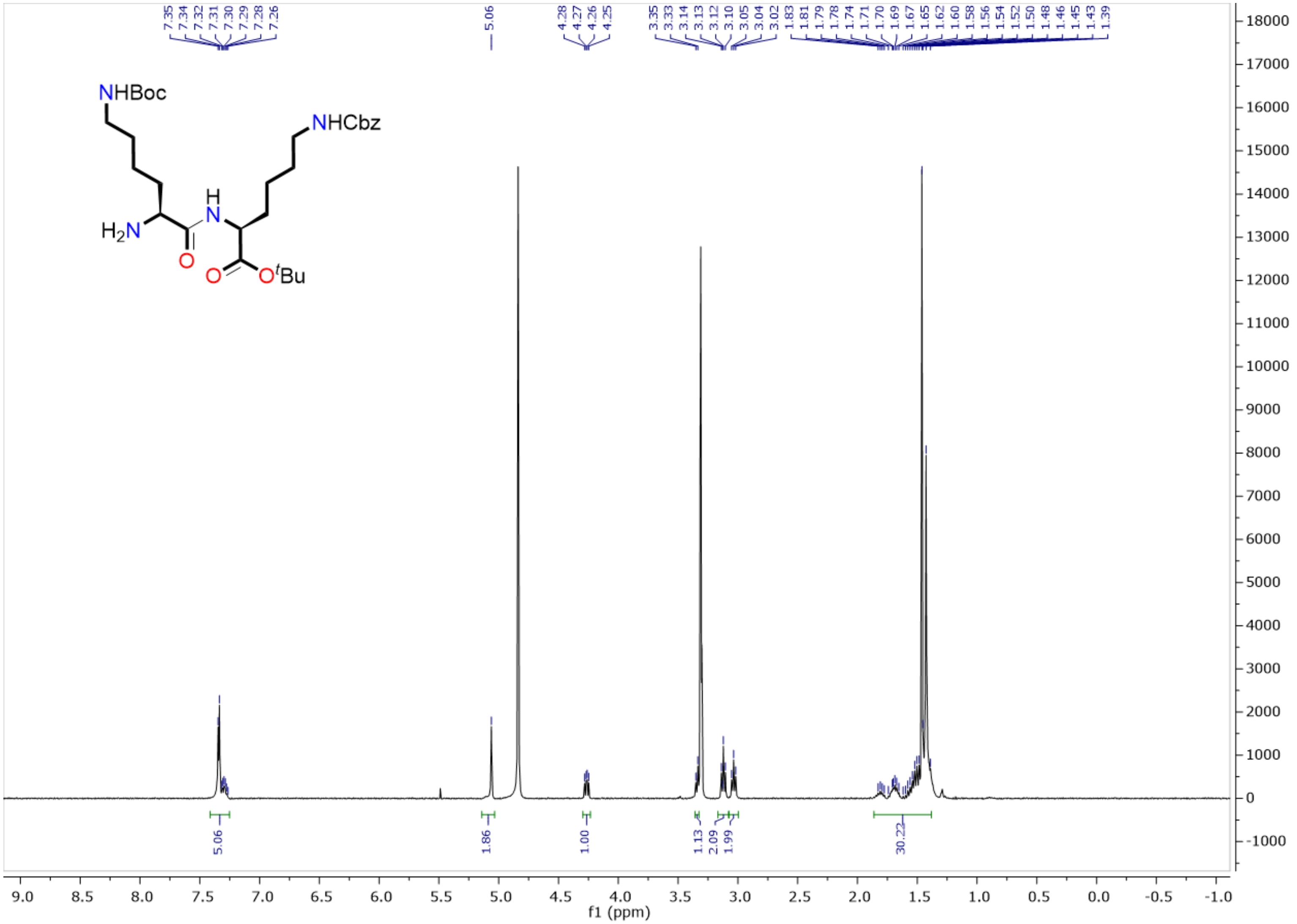

Figure S5. ^{1}H NMR spectrum of **2** (400 MHz, MeOD, 25°C).

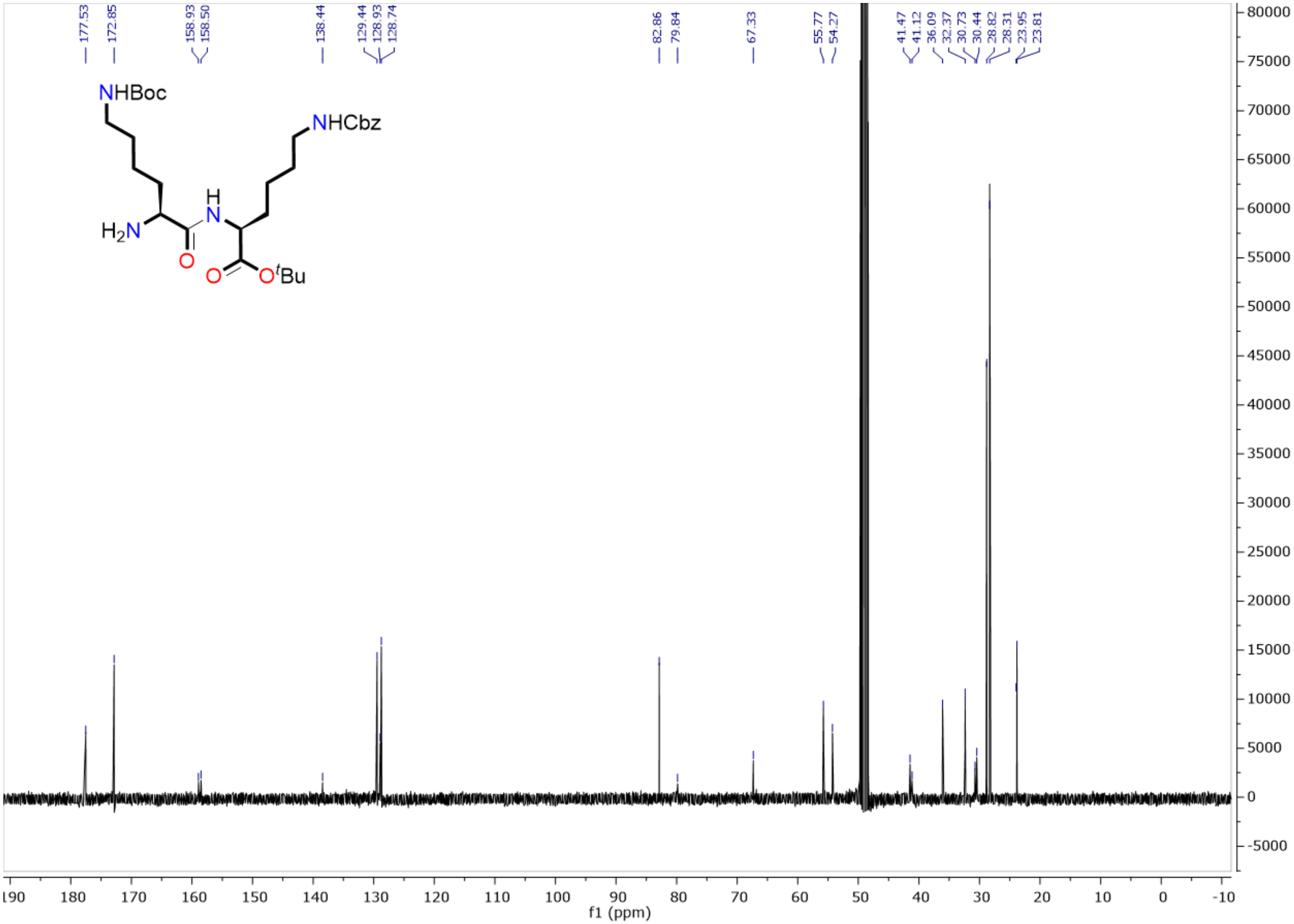

Figure S6. ^{13}C NMR spectrum of **2** (100 MHz, MeOD, 25°C).

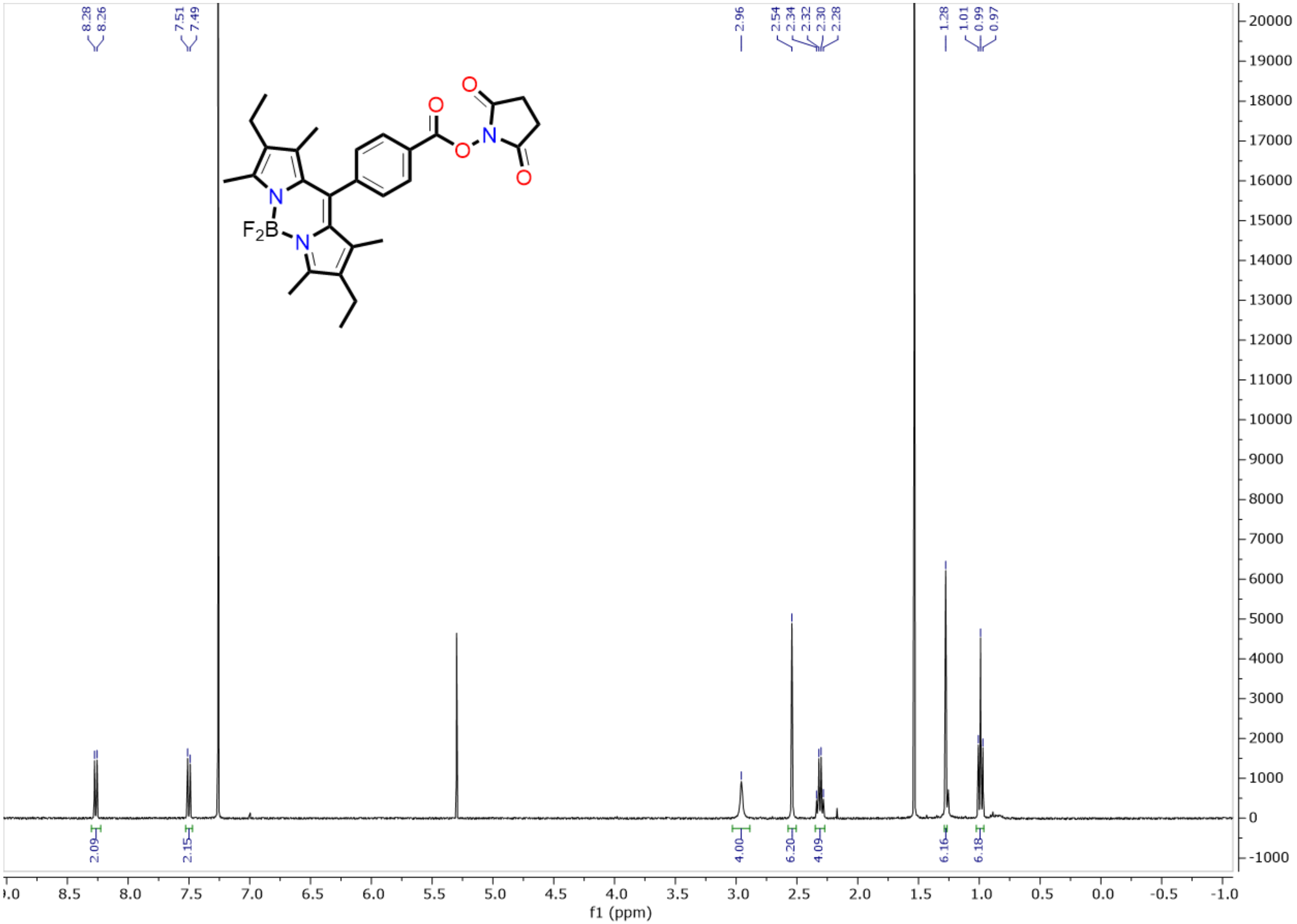

Figure S7. ^{1}H NMR spectrum of **1** (400 MHz, $CDCl_3$, 25°C).

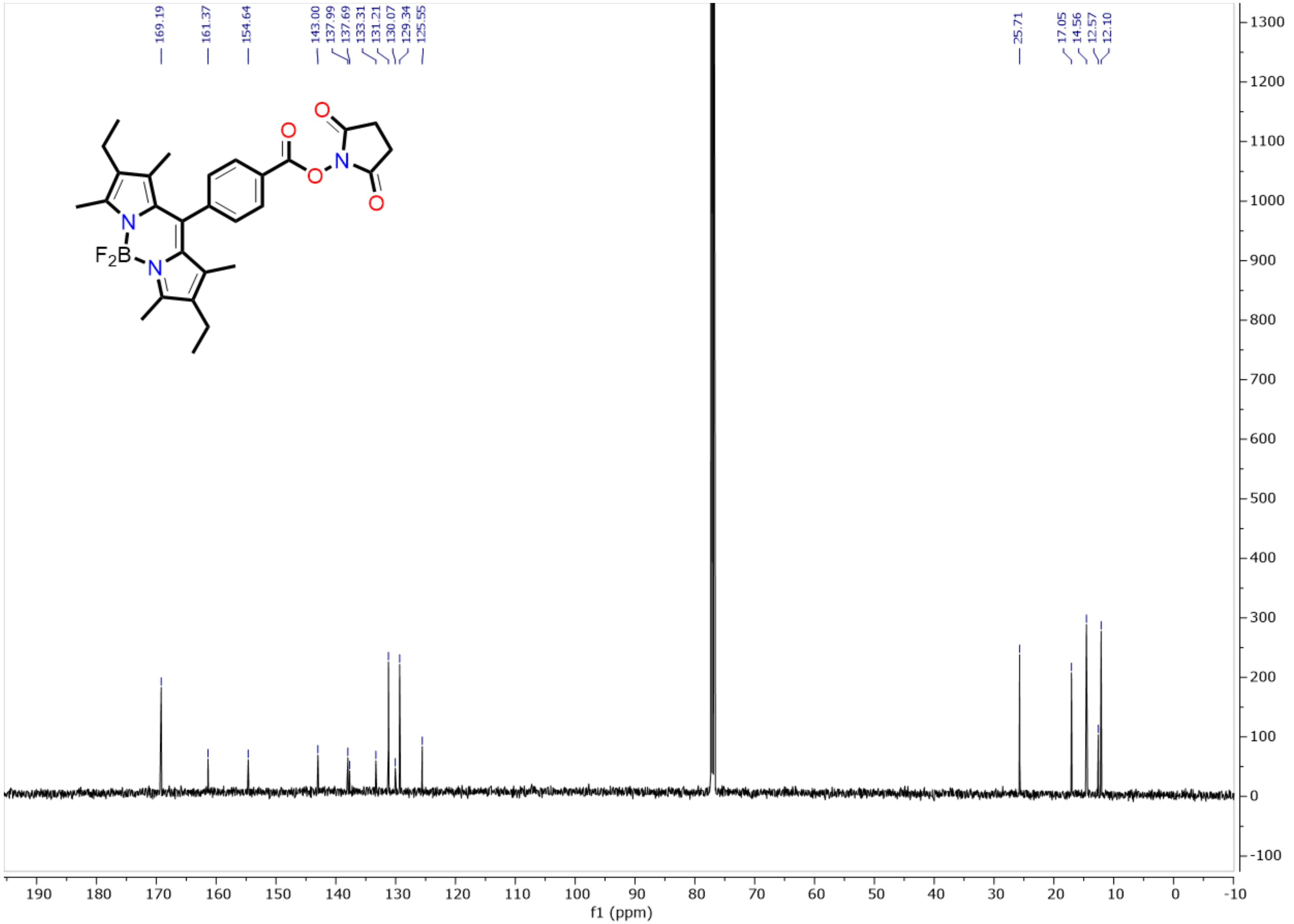

Figure S8. ^{13}C NMR spectrum of **1** (125 MHz, $CHCl_3$, 25°C).

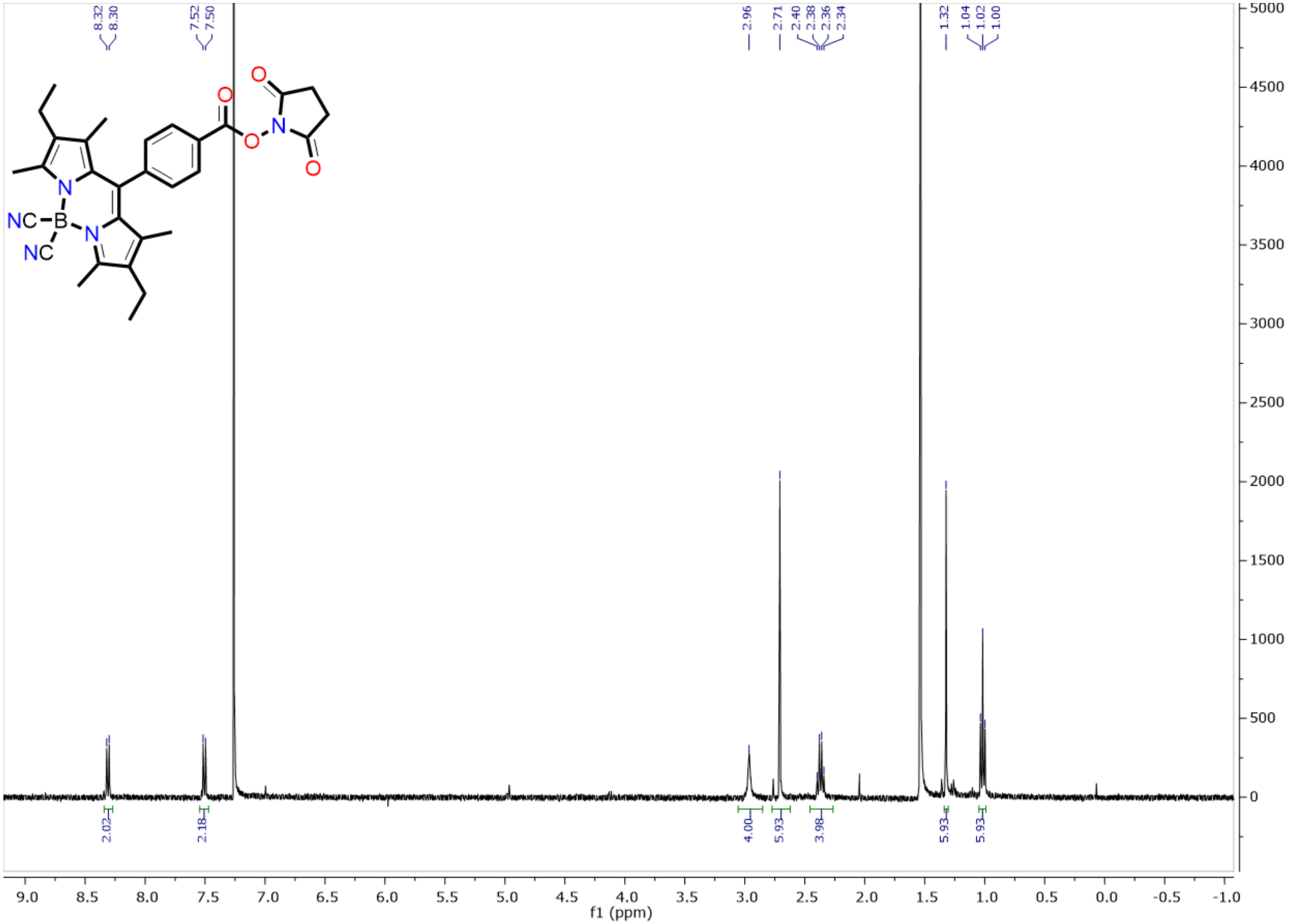

Figure S9. ^1H NMR spectrum of **7** (400 MHz, $CDCl_3$, 25°C).

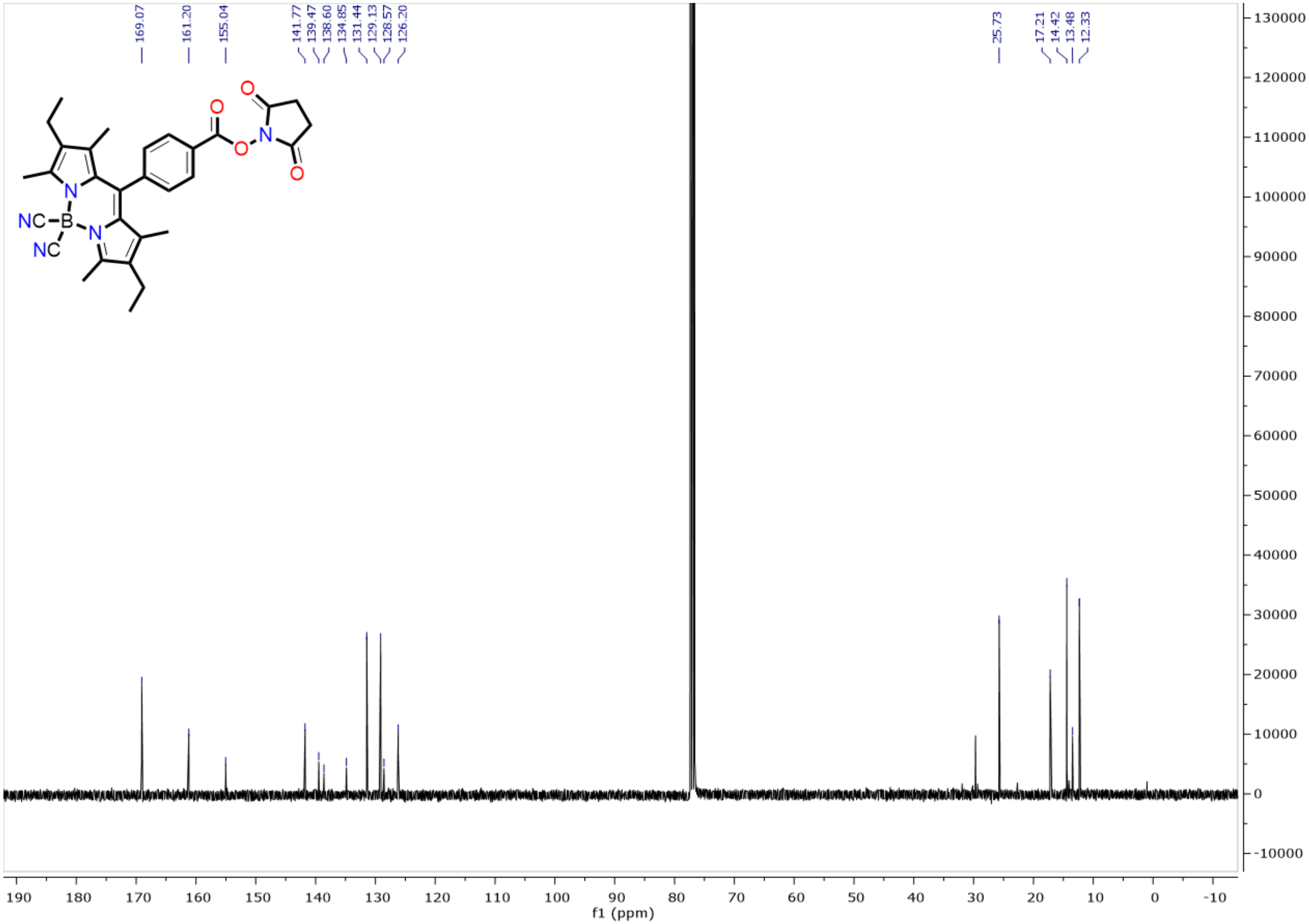

Figure S10. ^{13}C NMR spectrum of **7** (100 MHz, $CDCl_3$, 25°C).

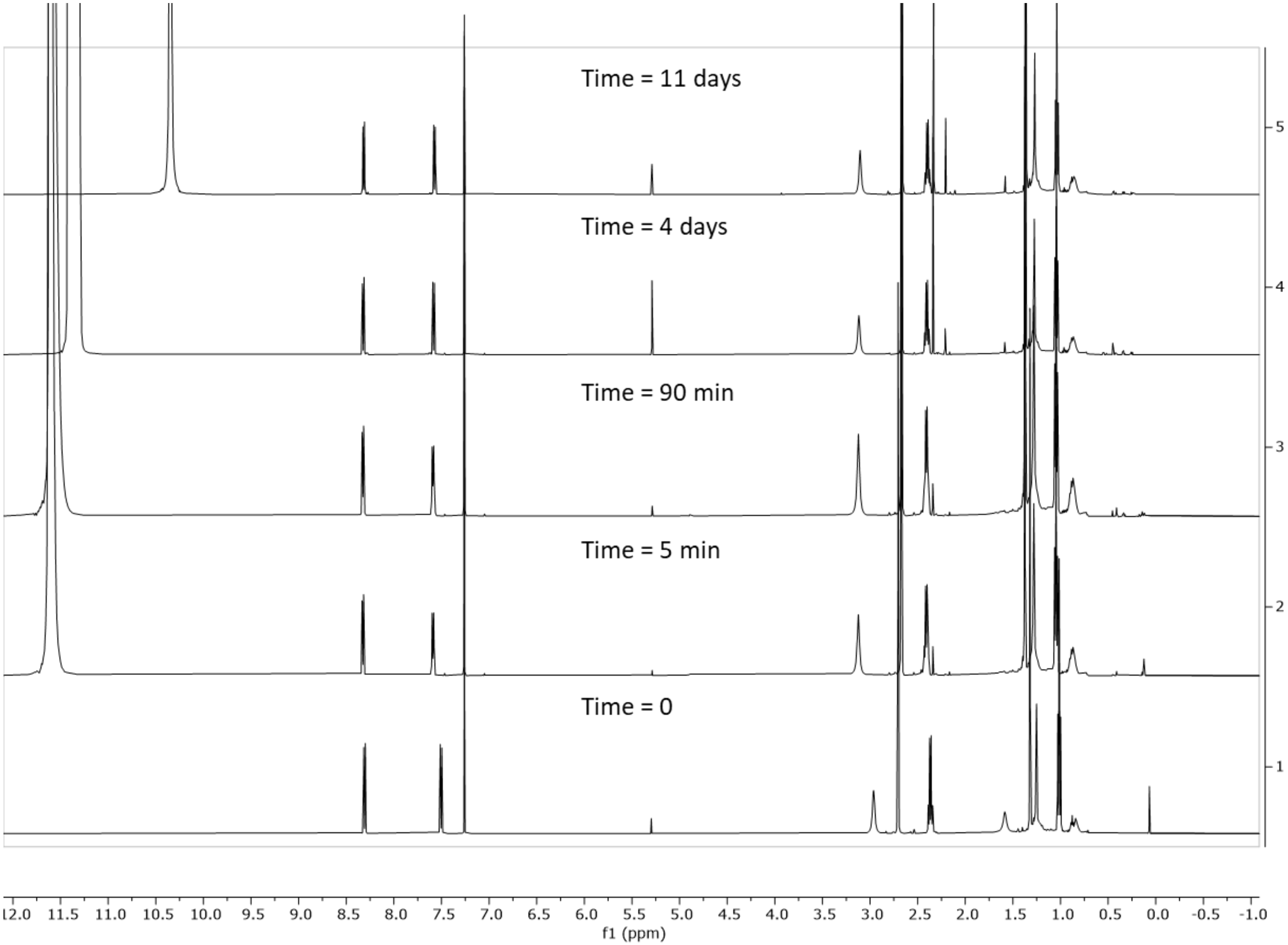

Figure S11. Investigating the stablitly of **7** in $CDCl_3$-TFA (25%) ^{1}H NMR (500 MHz, $CDCl_3$, 25°C).

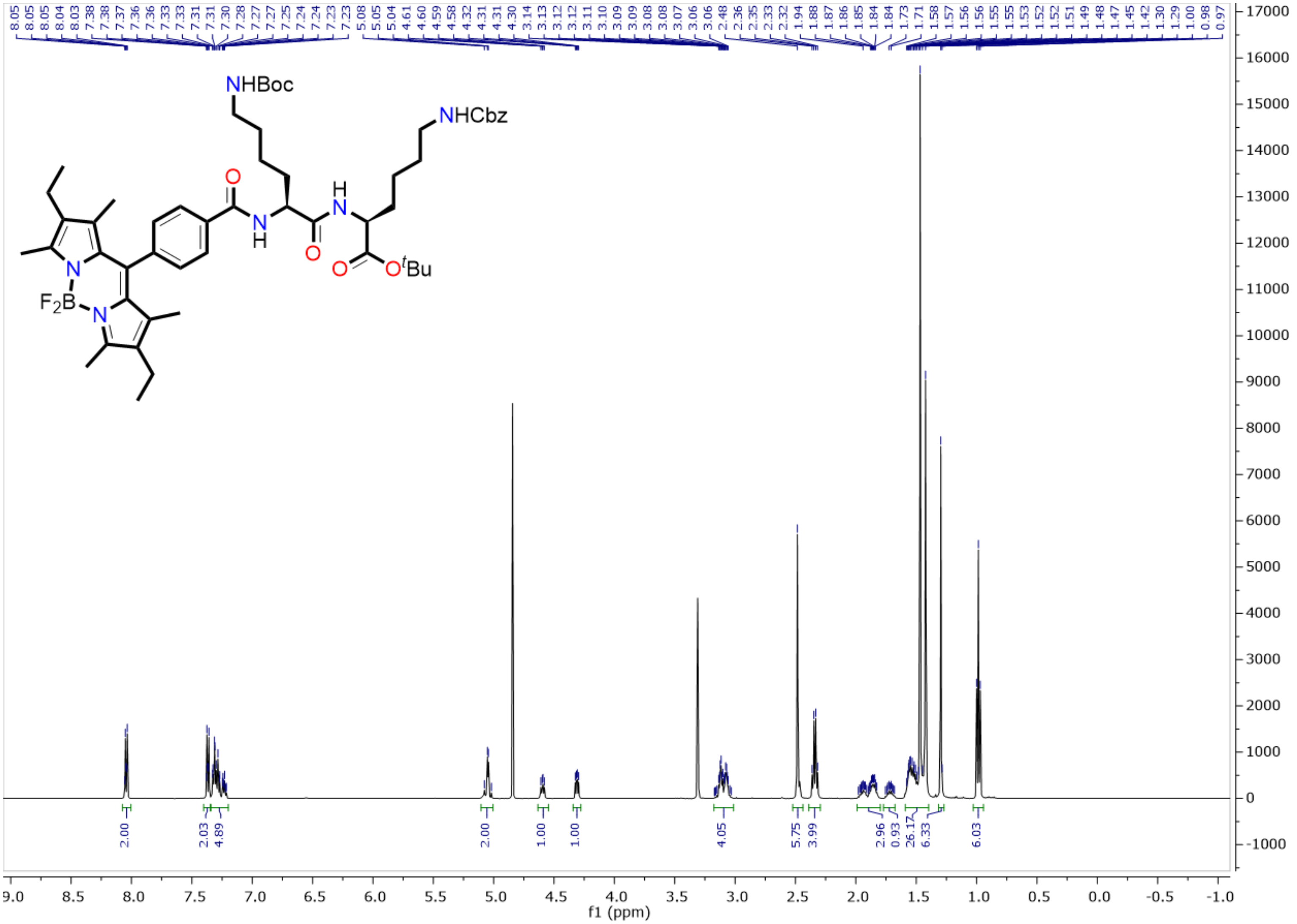

Figure S12. ^{1}H NMR spectrum of **3** (500 MHz, MeOD, 25°C).

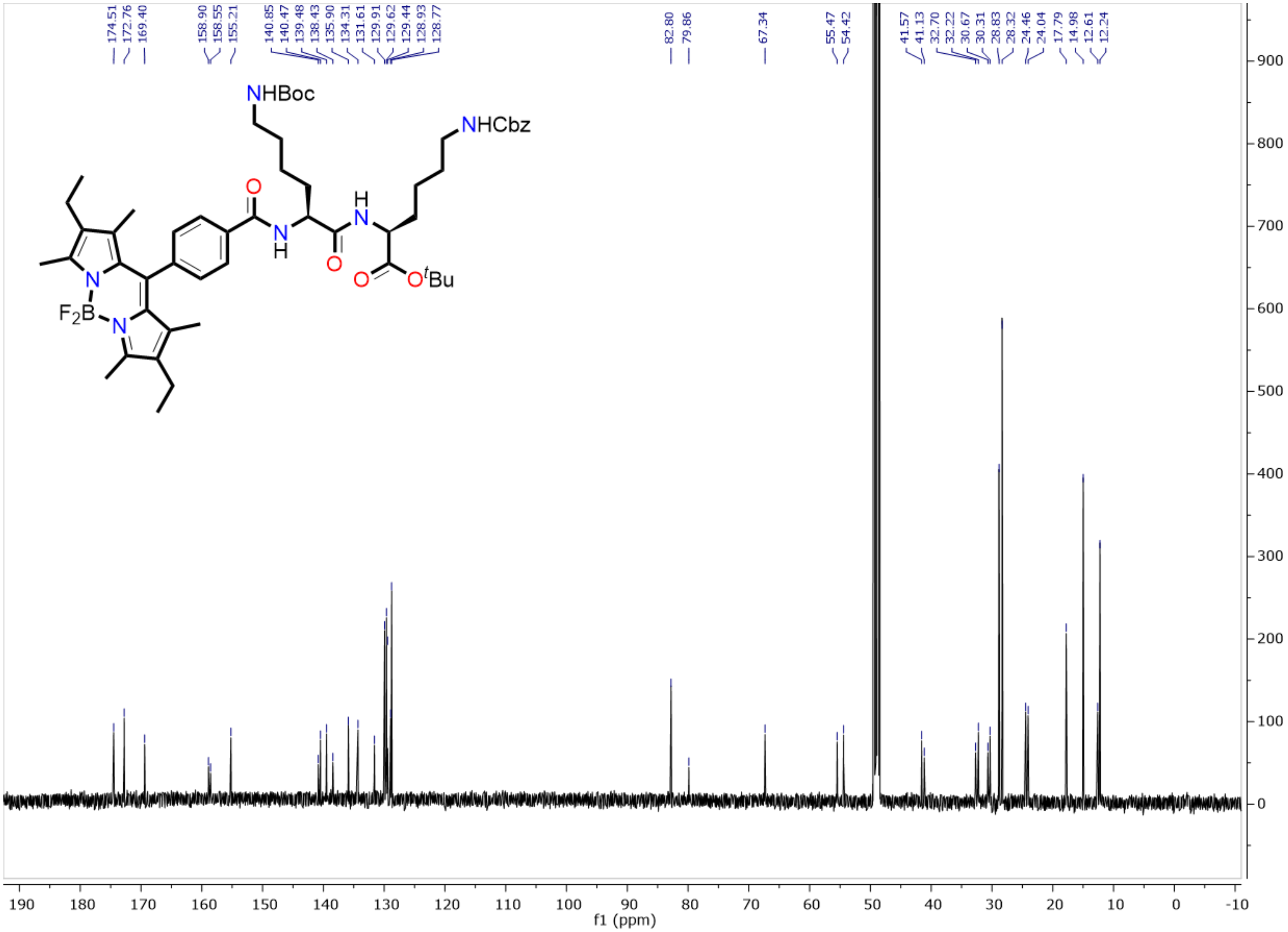

Figure S13. ^{13}C NMR spectrum of **3** (125 MHz, MeOD, 25°C).

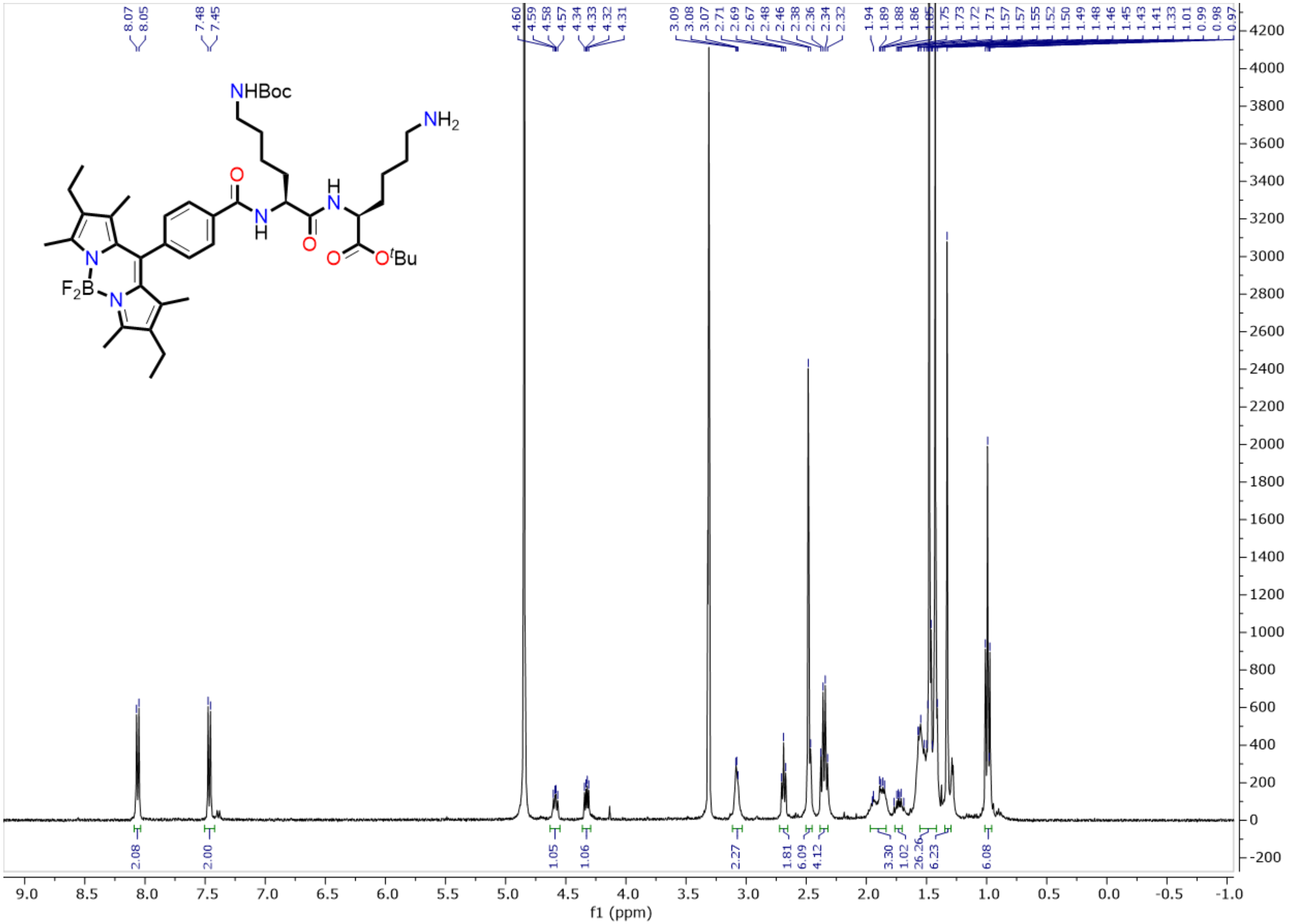

Figure S14. ^{1}H NMR spectrum of **4** (400 MHz, MeOD, 25°C).

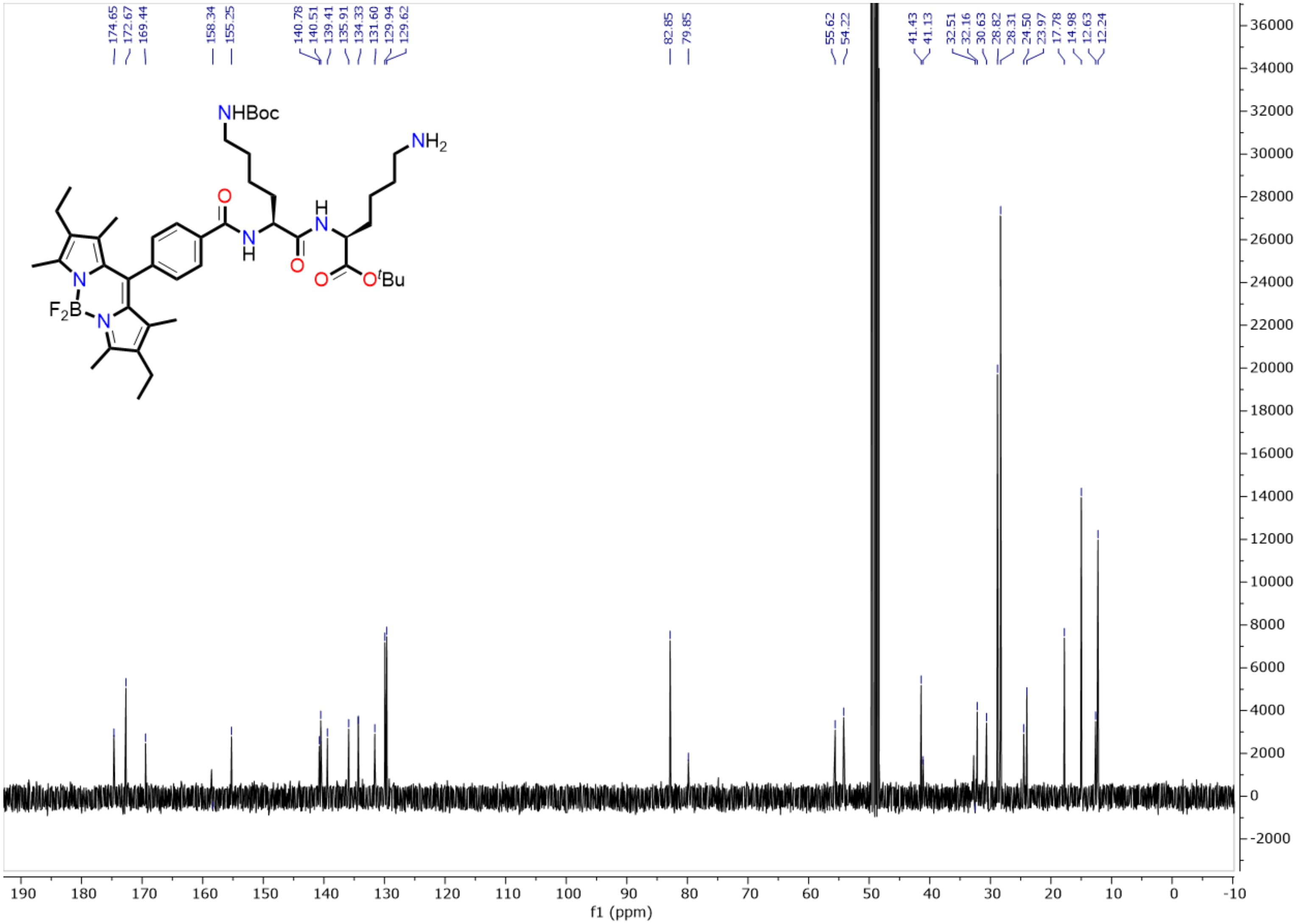

Figure S15. ^{13}C NMR spectrum of **4** (100 MHz, MeOD, 25°C).

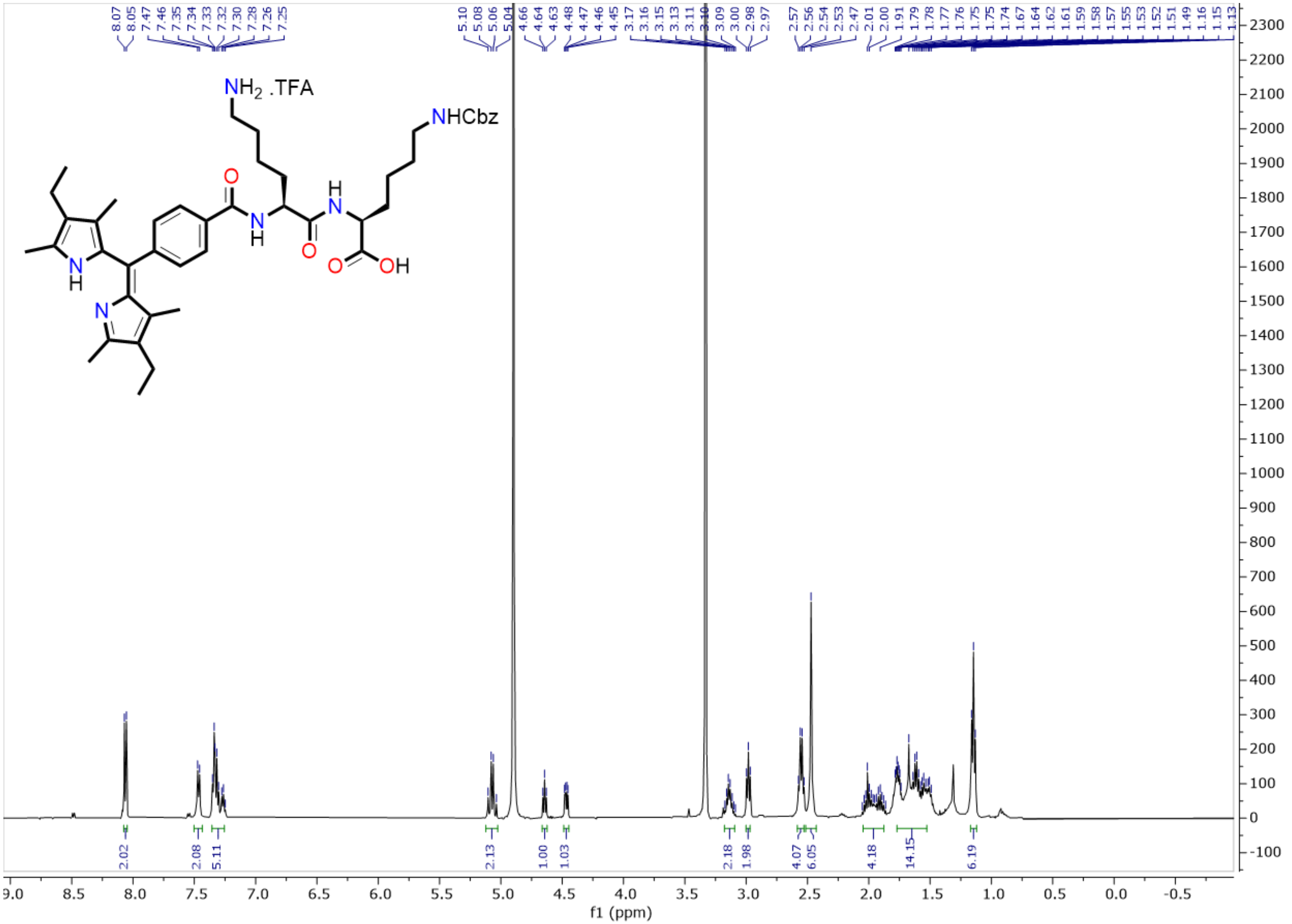

Figure S16. ^{1}H NMR spectrum of **6** (500 MHz, MeOD, 25°C).

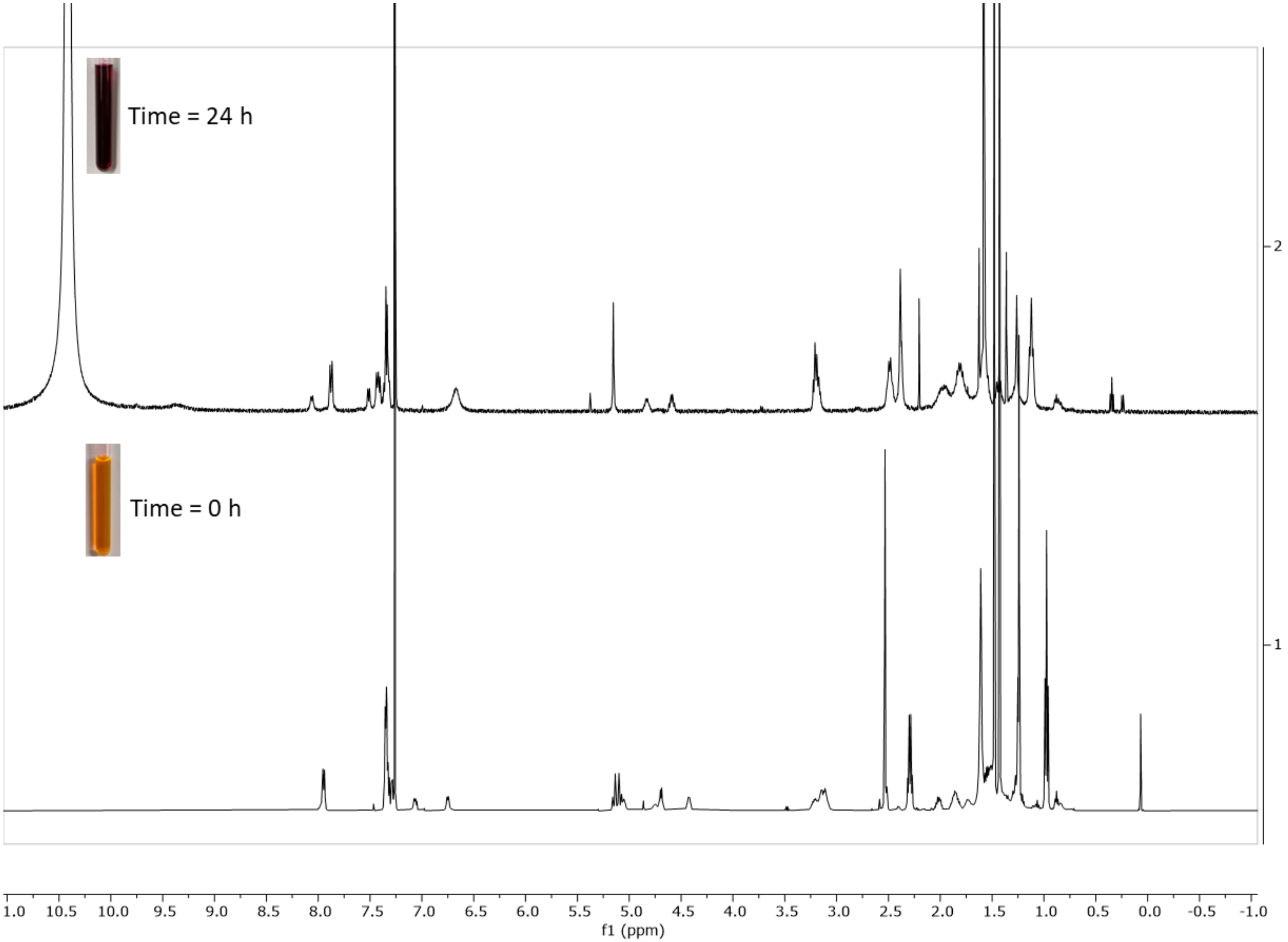

Figure S17. ^{1}H NMR of trasformation **3** to **6** at t = 0 h & t = 24 h (500 MHz, $CDCl_3$).

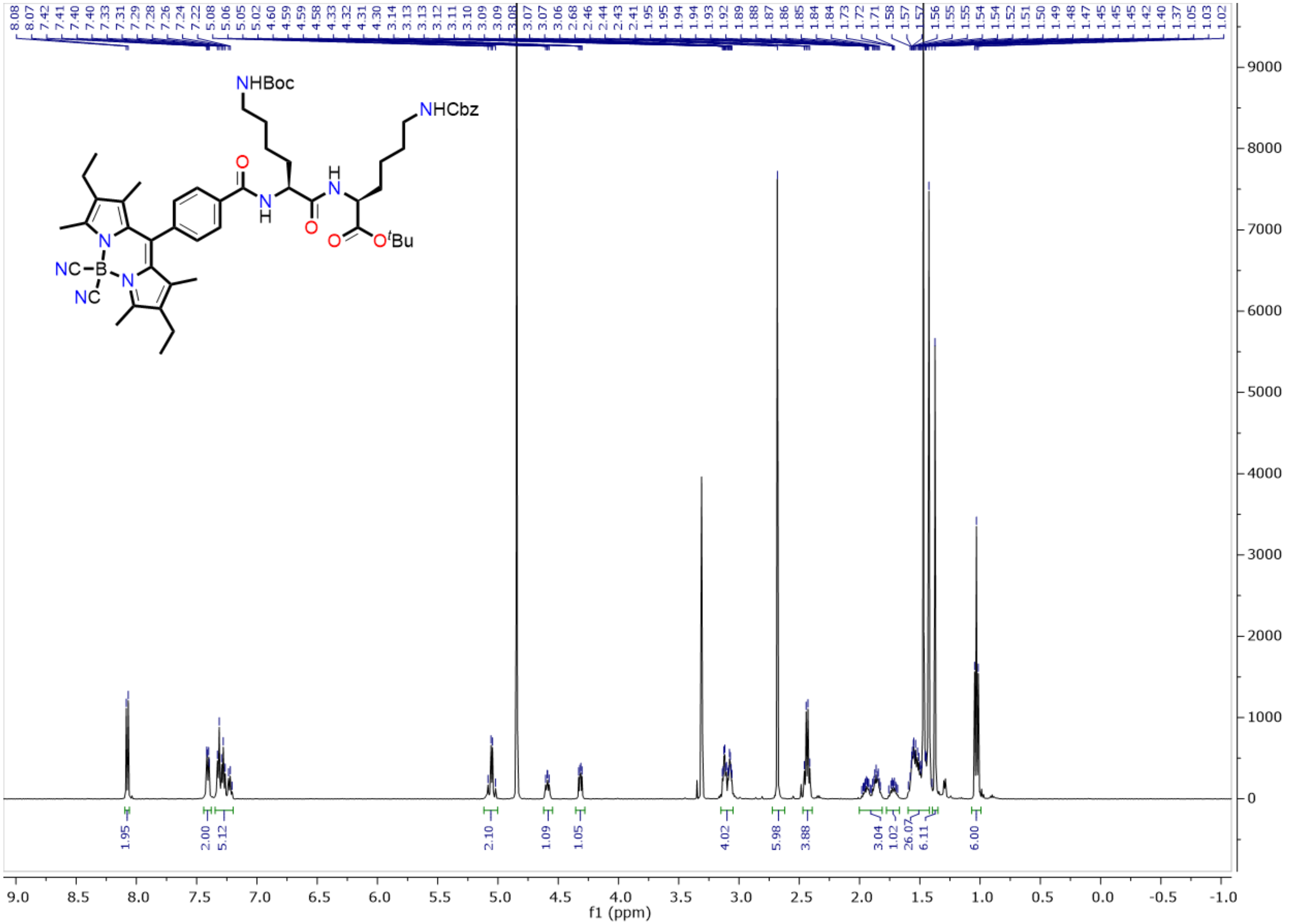

Figure S18. ^{1}H NMR spectrum of **8** (500 MHz, MeOD, 25°C).

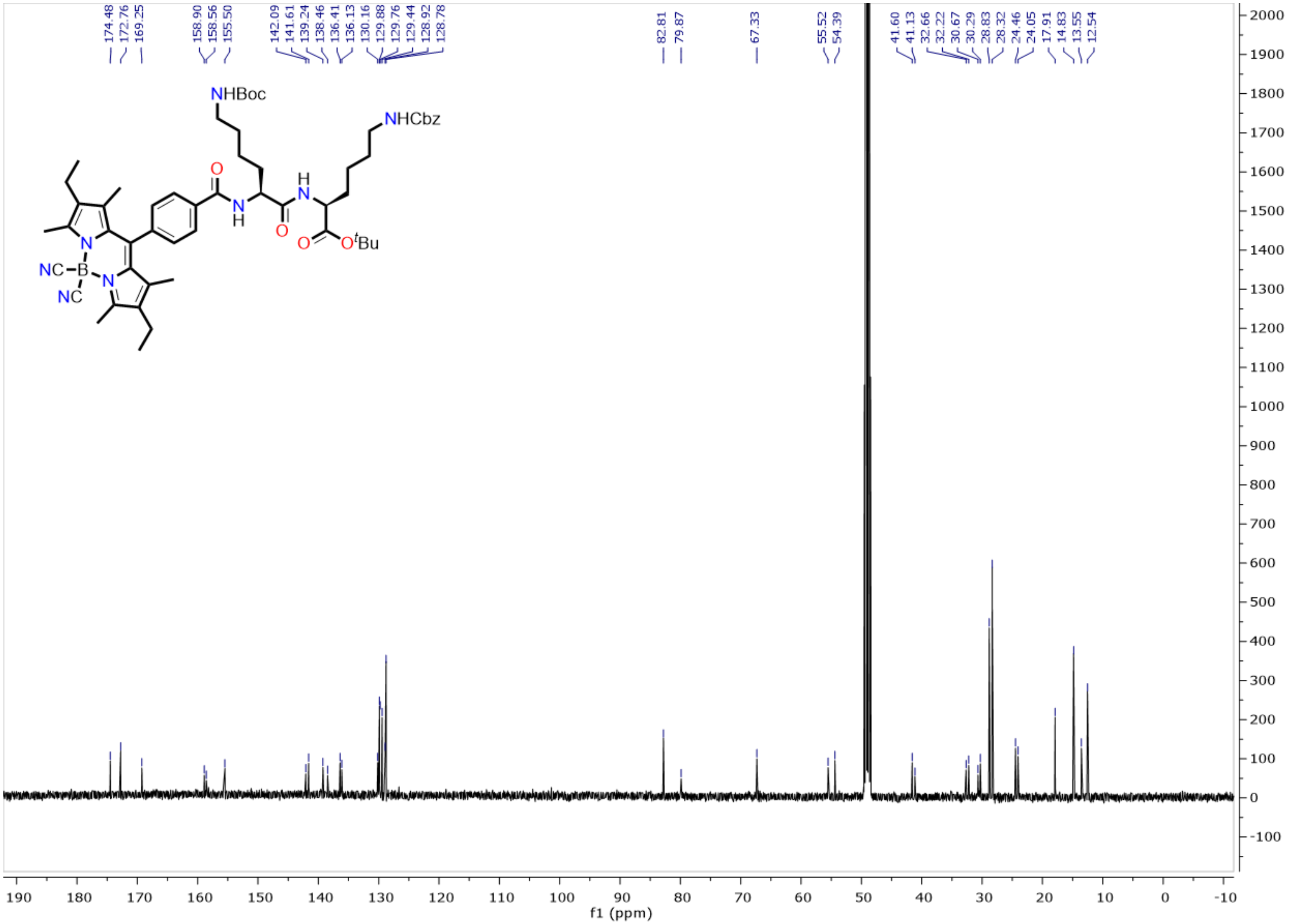

Figure S19. ^{13}C NMR spectrum of **8** (125 MHz, MeOD, 25°C).

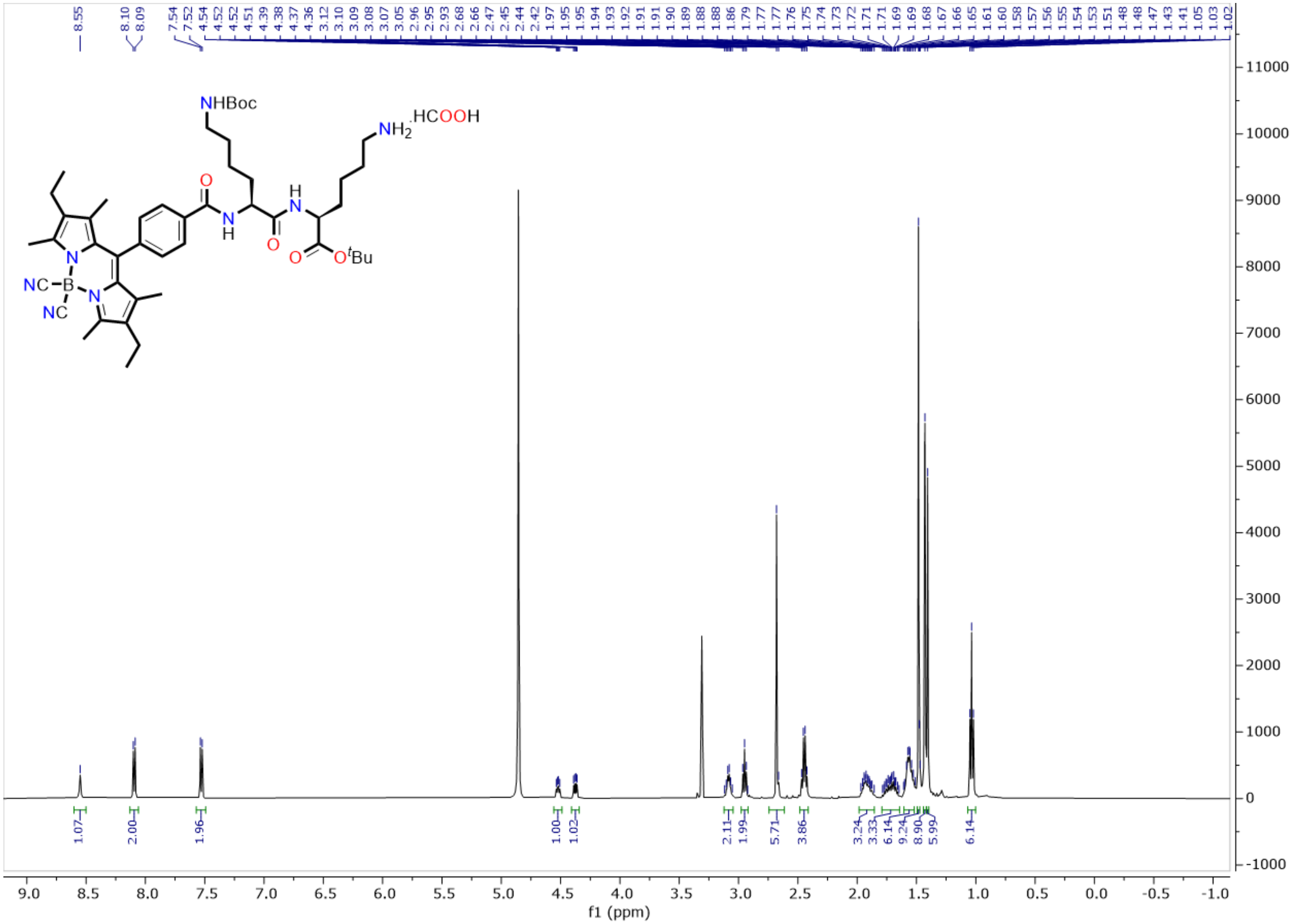

Figure S20. ^{1}H NMR spectrum of **22** (500 MHz, MeOD, 25°C).

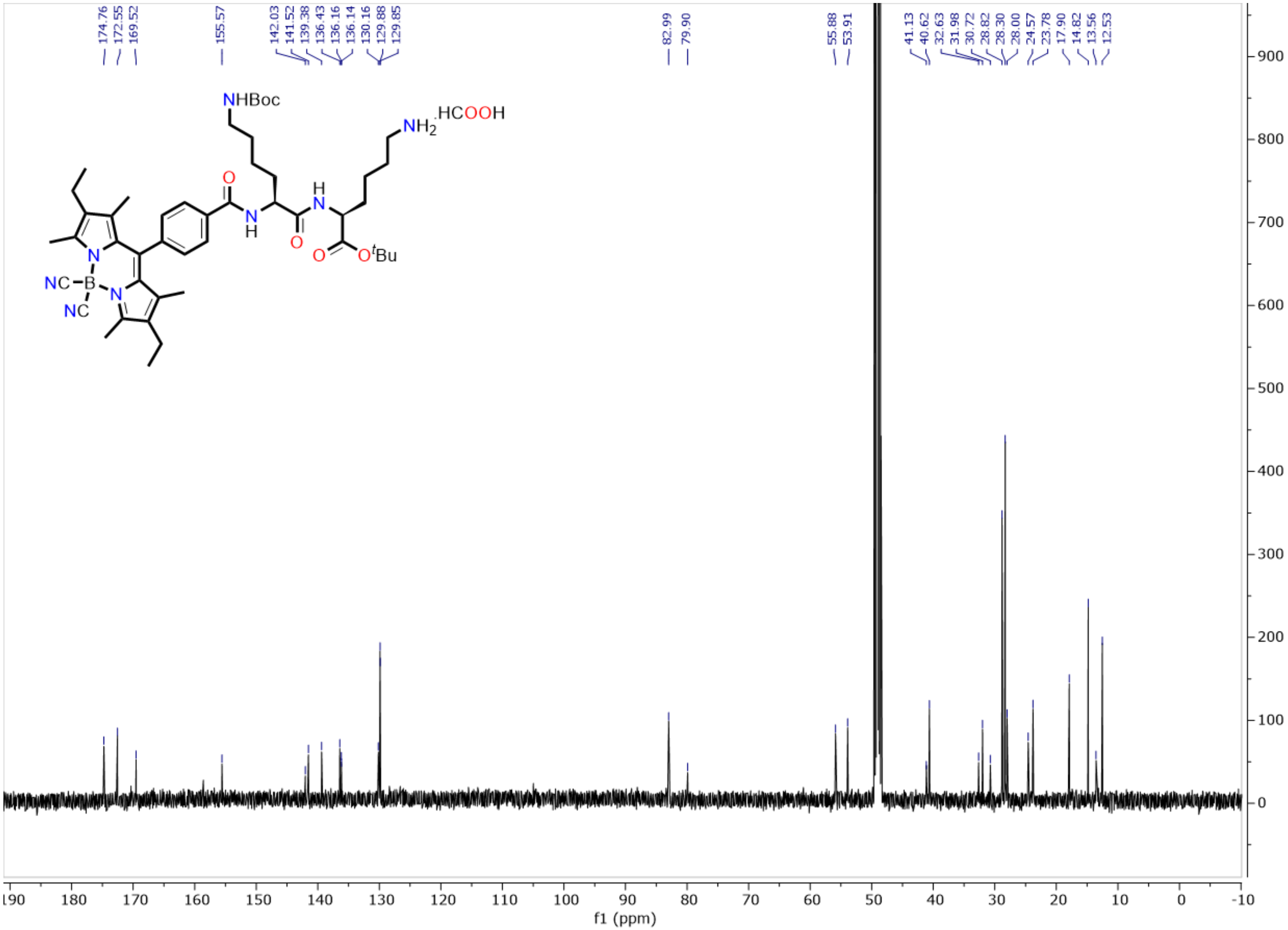

Figure S21. ^{13}C NMR spectrum of **22** (125 MHz, MeOD, 25°C).

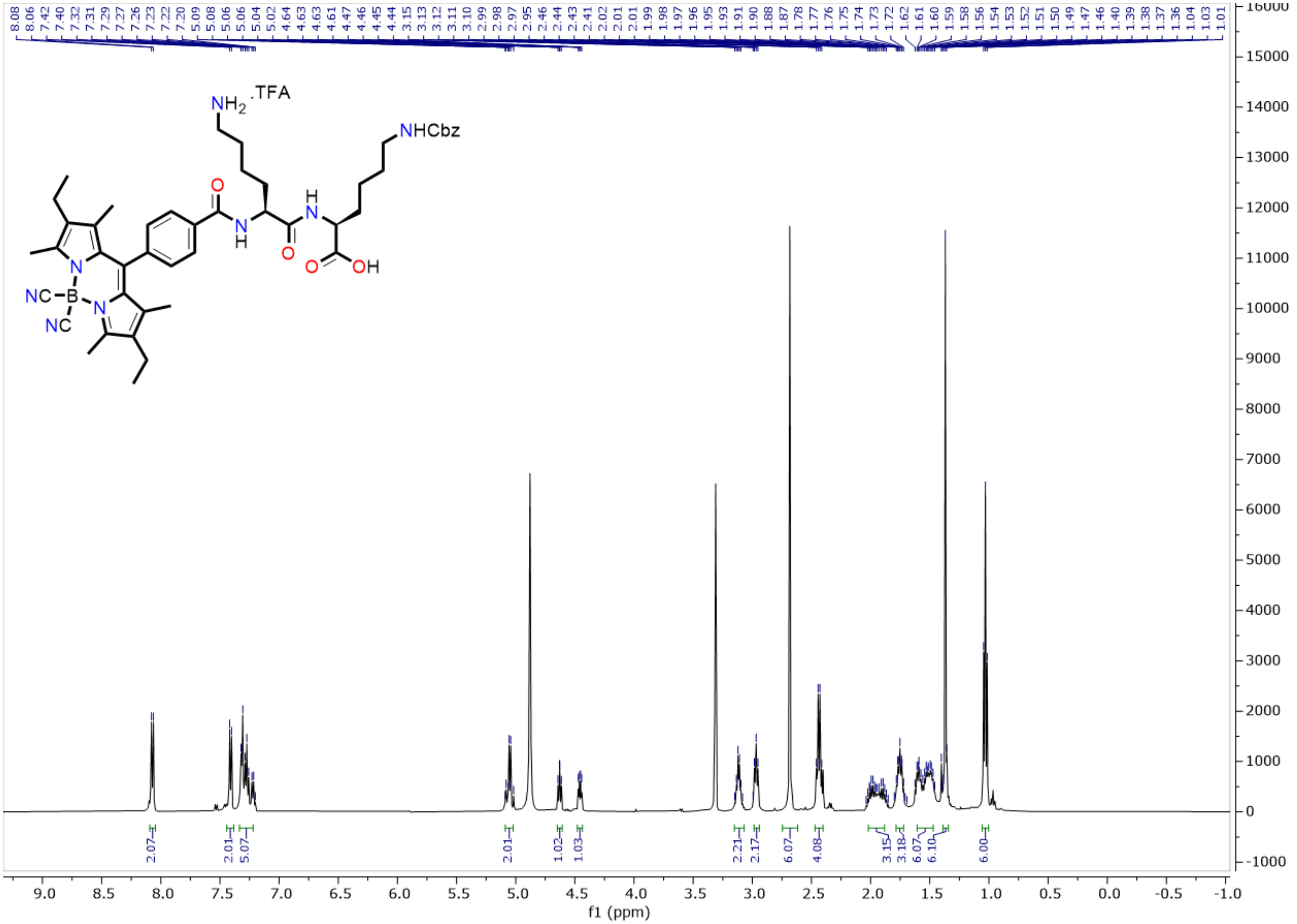

Figure S22. ^{1}H NMR spectrum of **9** (500 MHz, MeOD, 25°C).

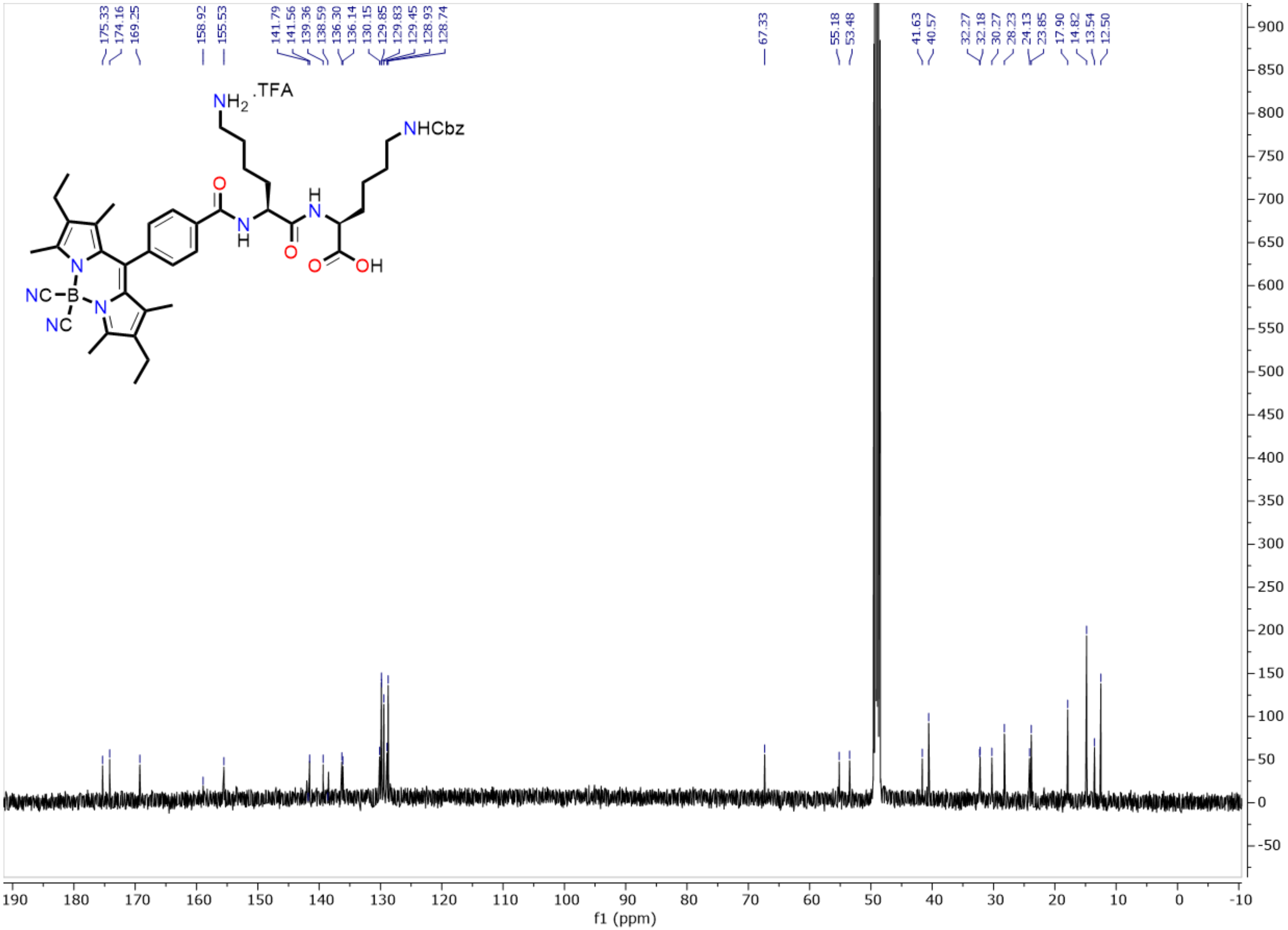

Figure S23. ^{13}C NMR spectrum of **9** (125 MHz, MeOD, 25°C).

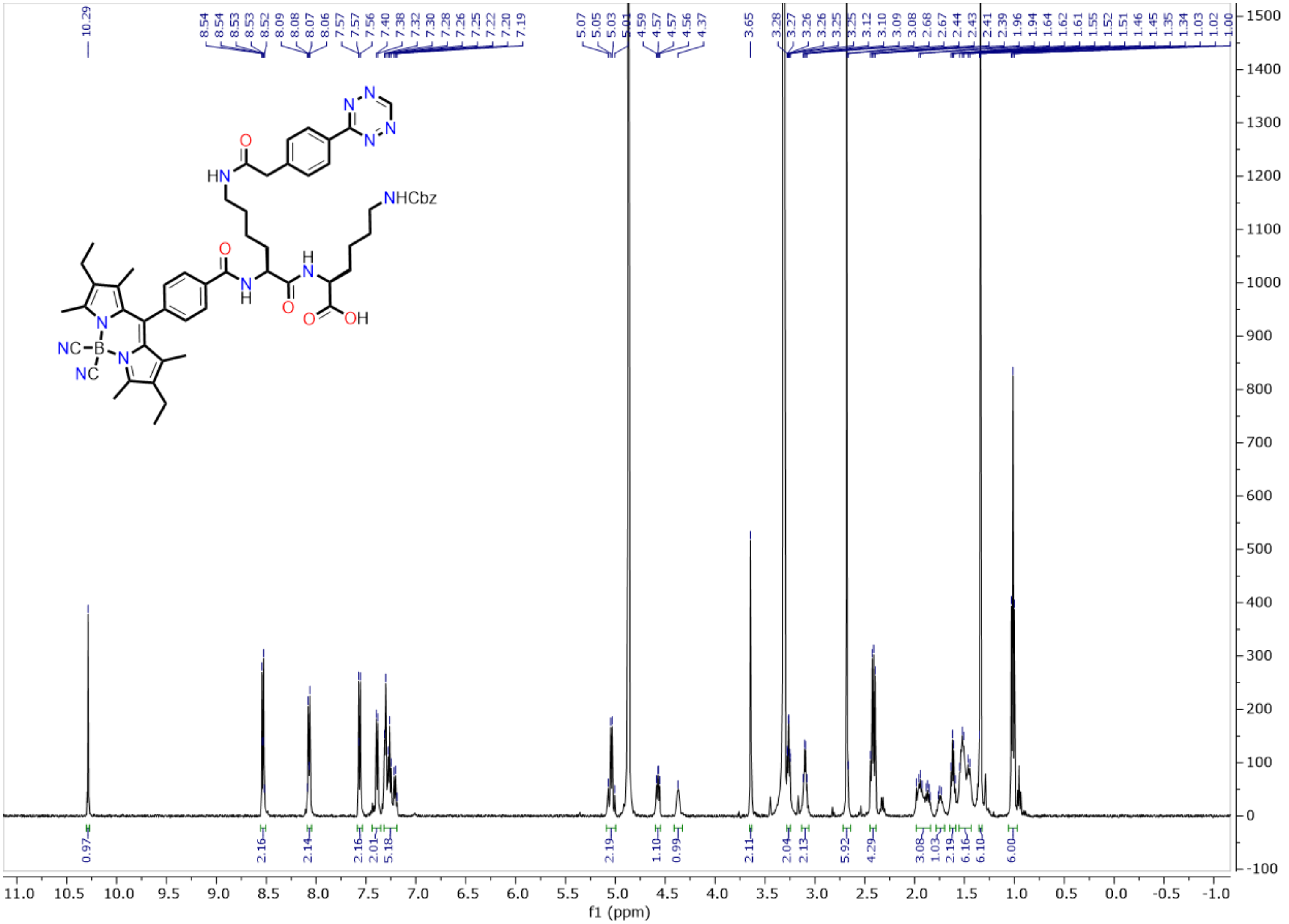

Figure S24. ^{1}H NMR spectrum of **11** (500 MHz, MeOD, 25°C).

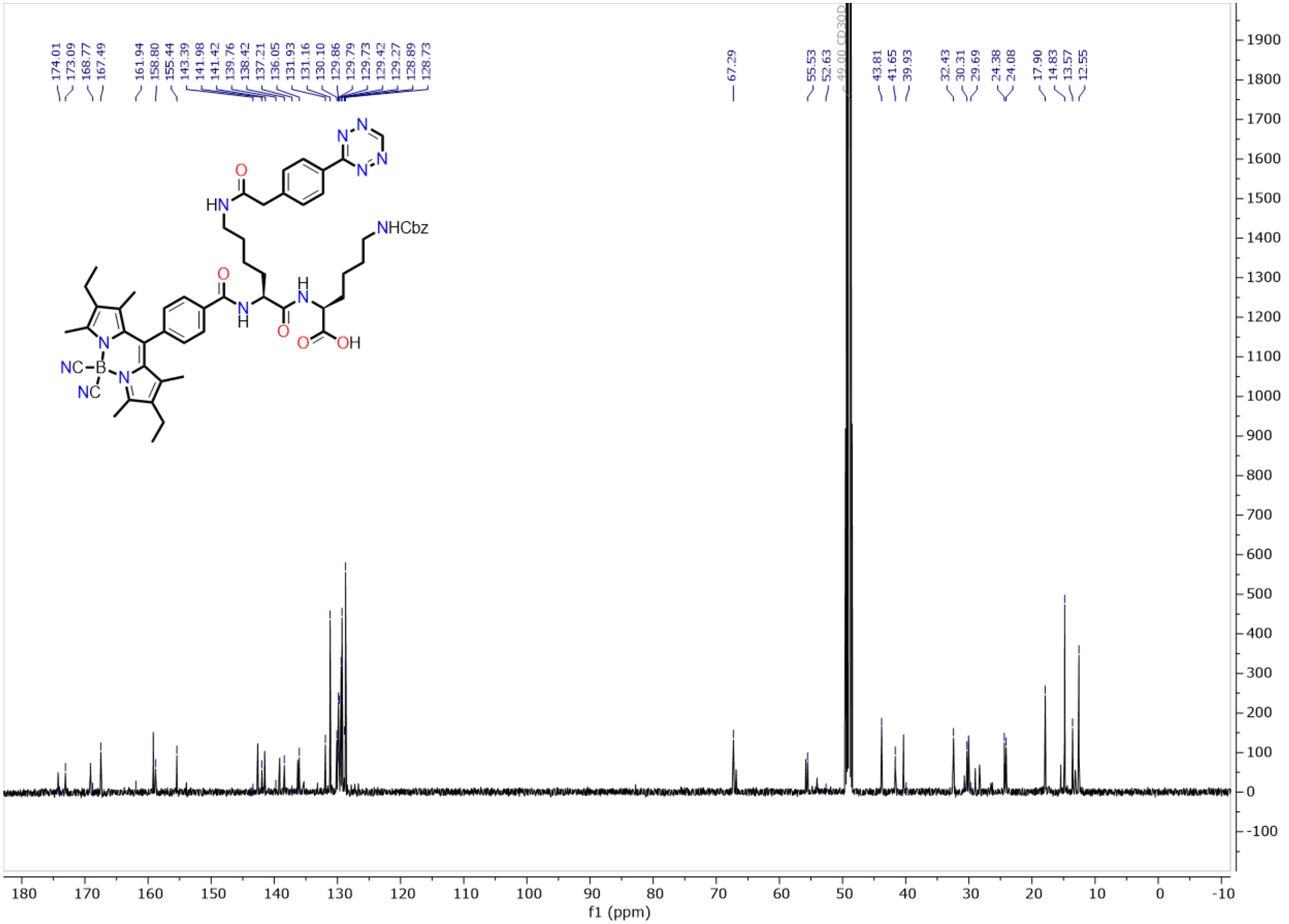

Figure S25. ^{13}C NMR spectrum of **11** (125 MHz, MeOD, 25°C).

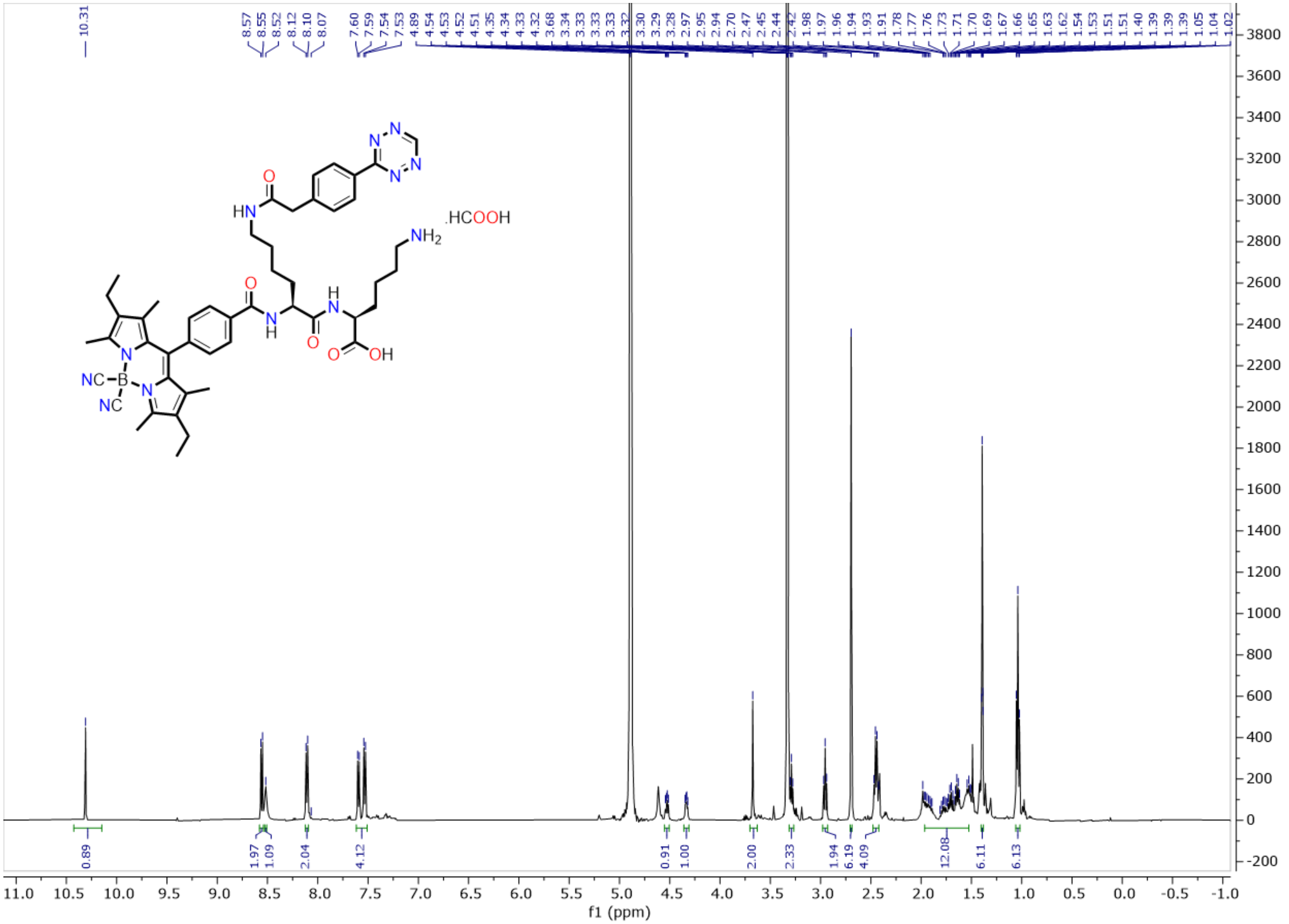

Figure S26. ^{1}H NMR spectrum for the formate salt of **12** (500 MHz, MeOD, 25°C).

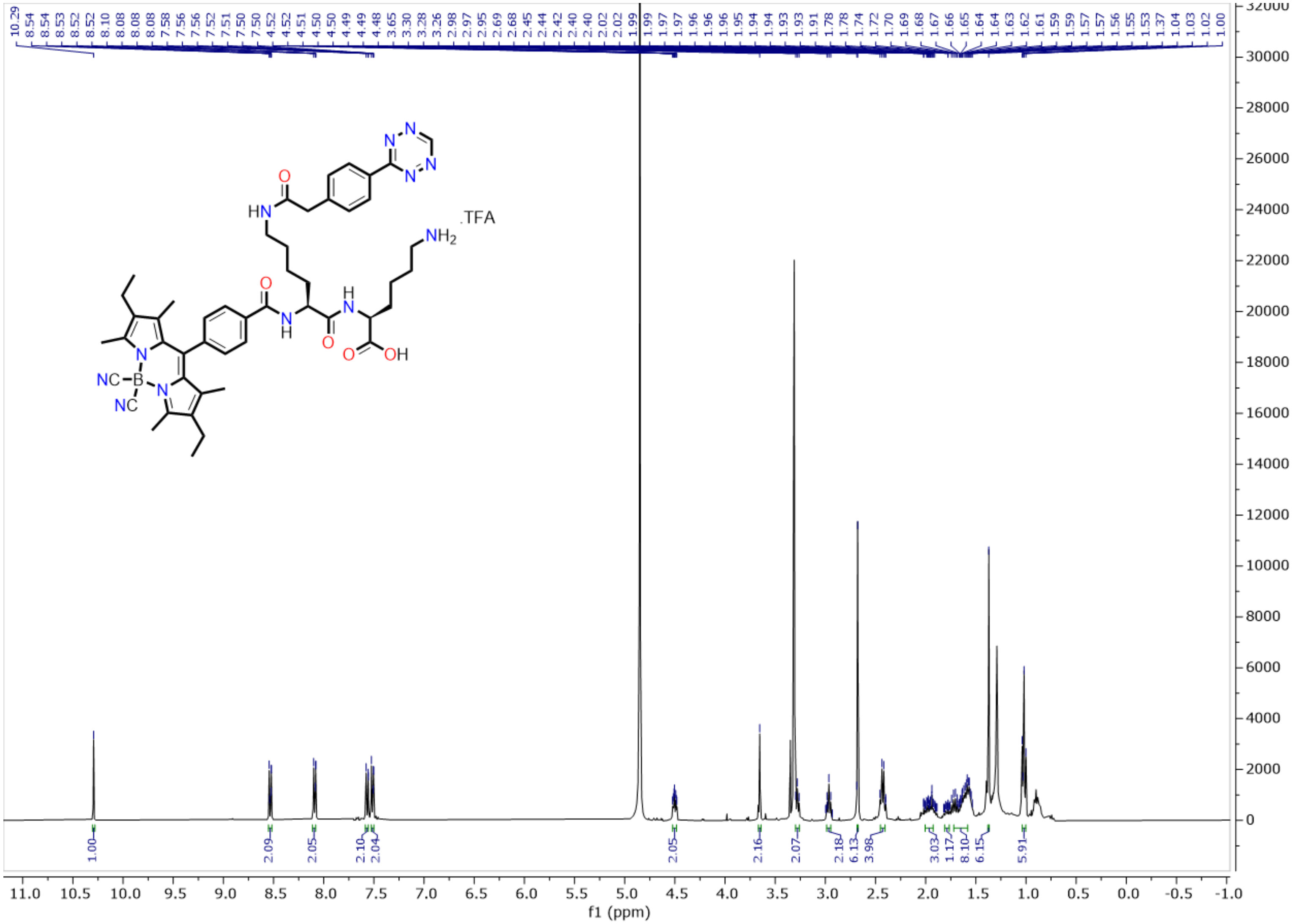

Figure S27. ^{1}H NMR spectrum for the TFA salt of **12** (500 MHz, MeOD, 25°C).

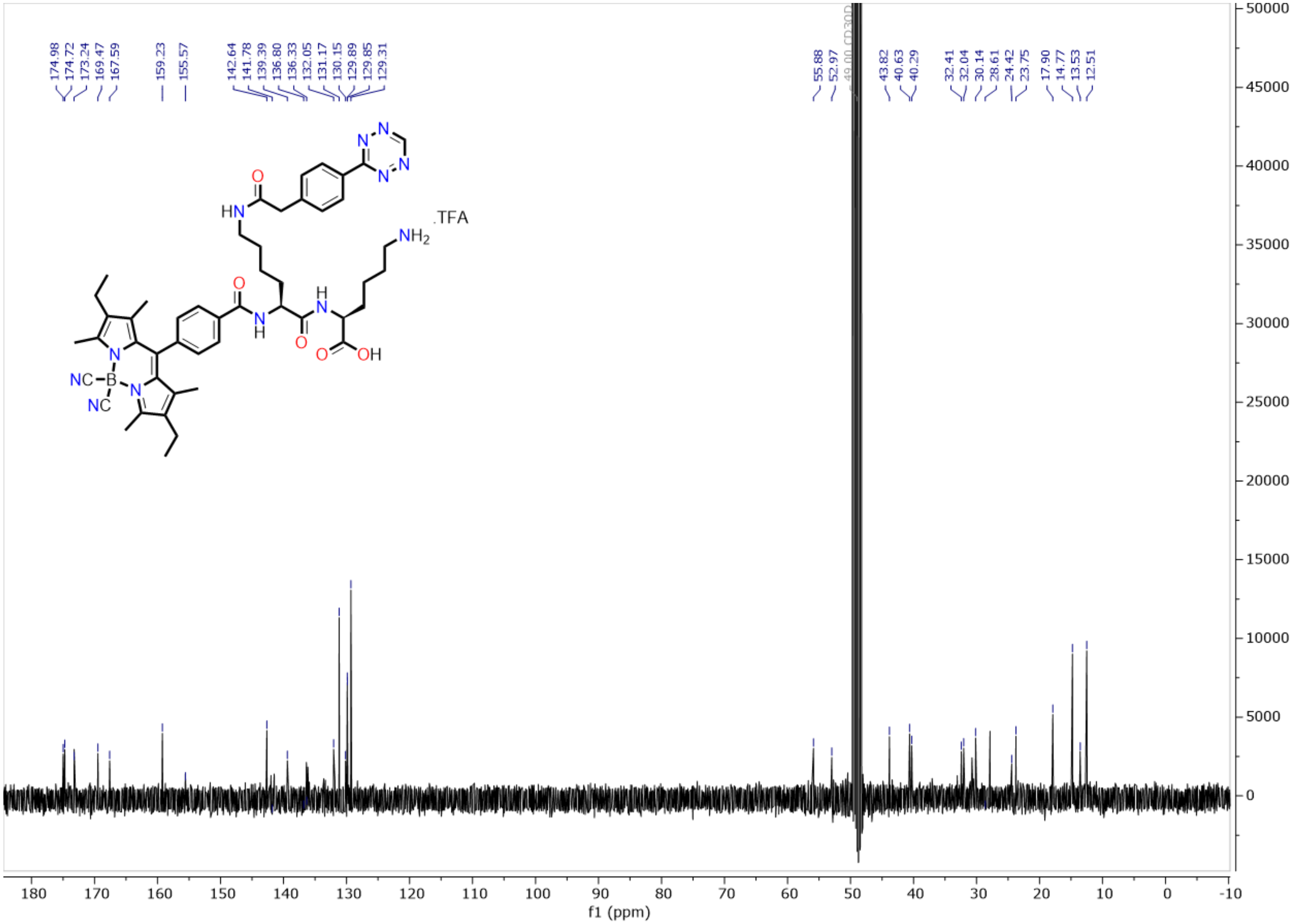

Figure S28. ^{13}C NMR spectrum for the TFA salt of **12** (125 MHz, MeOD, 25°C).

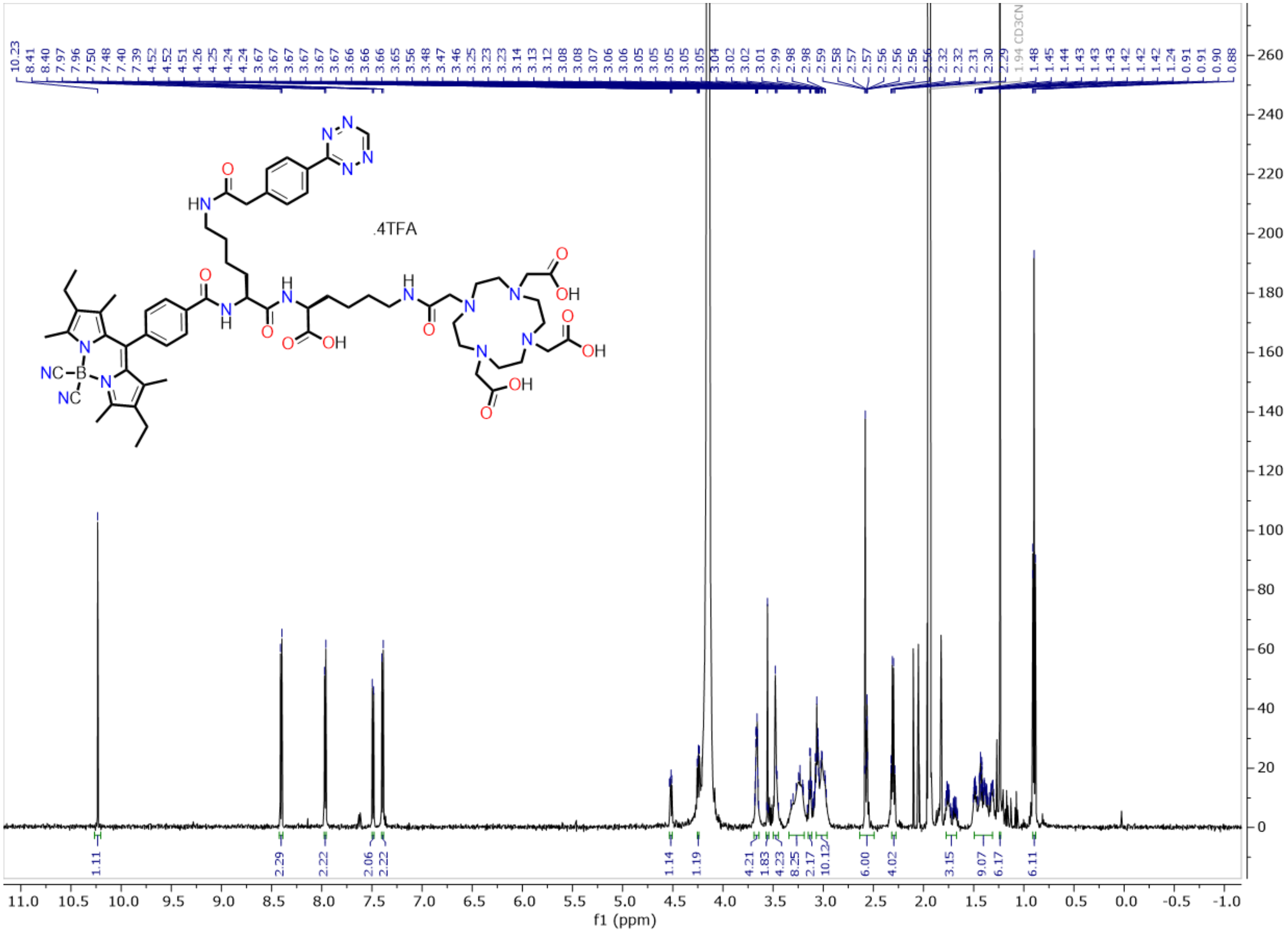

Figure S29. ^{1}H NMR spectrum of **13** (600 MHz, CD_3CN-D_2O, 25°C).

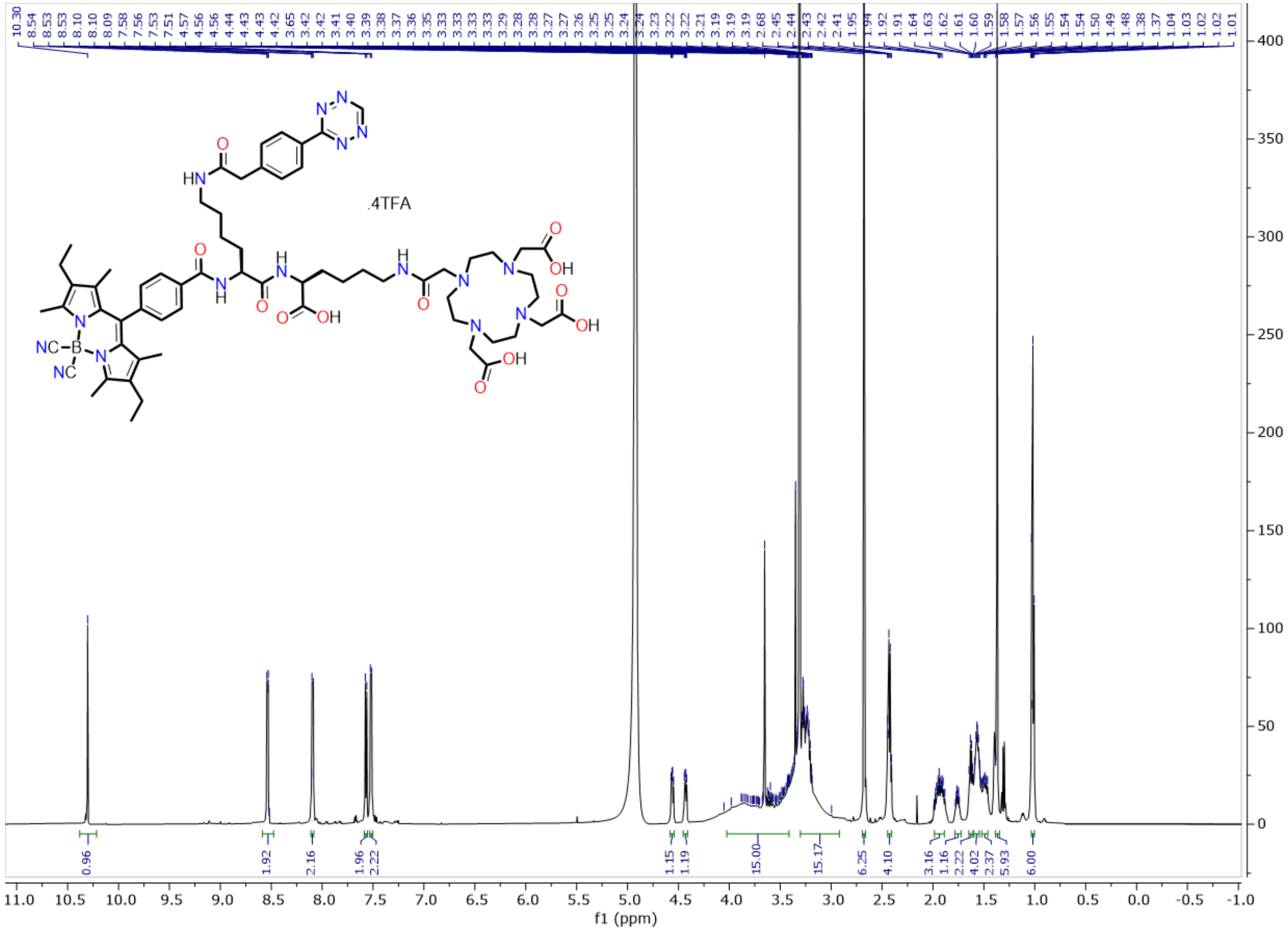

Figure S30. ^{1}H NMR spectrum of **13** (600 MHz, MeOD, 25°C).

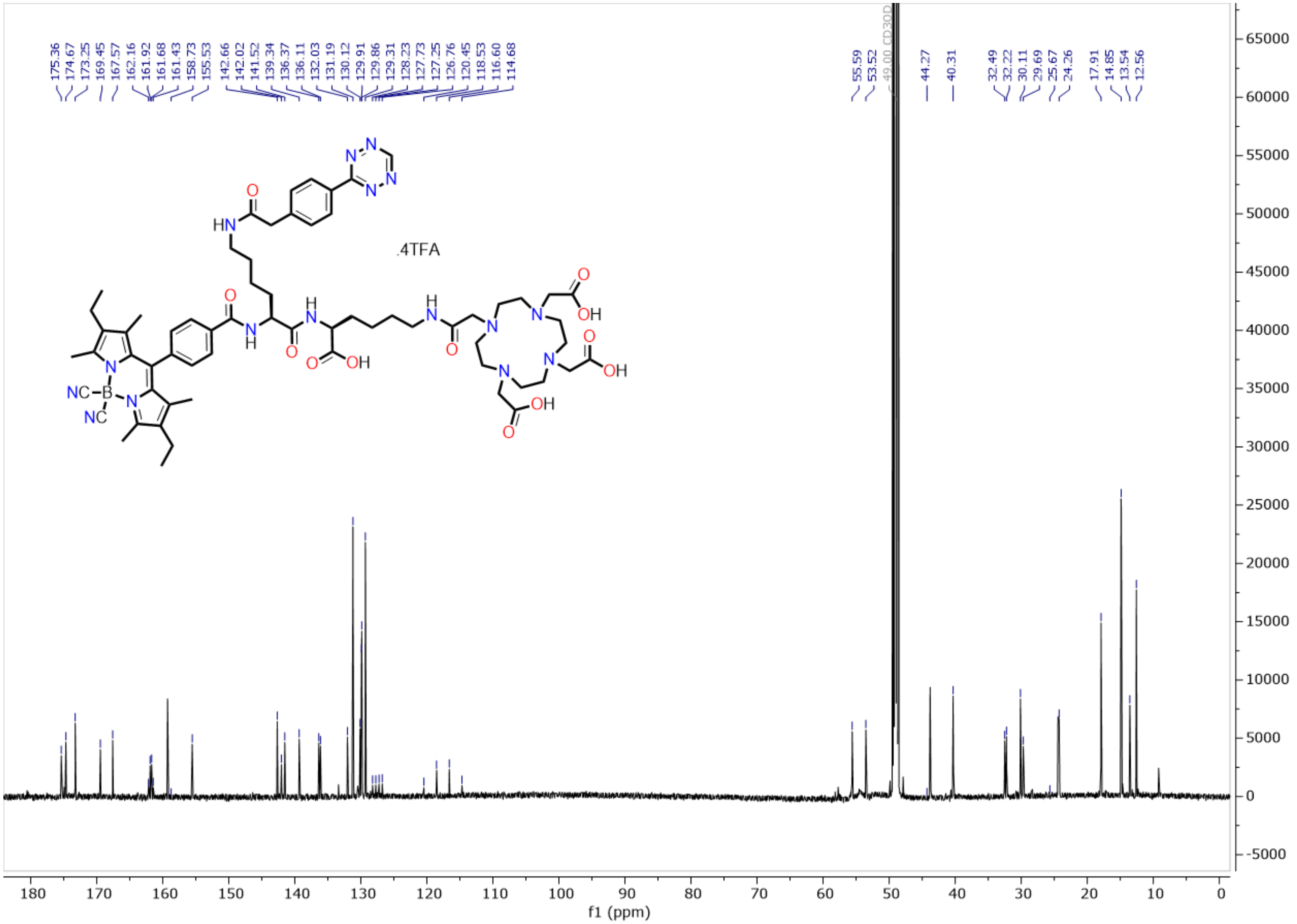

Figure S31. ^{13}C NMR spectrum of **13** (150 MHz, MeOD, 25°C).

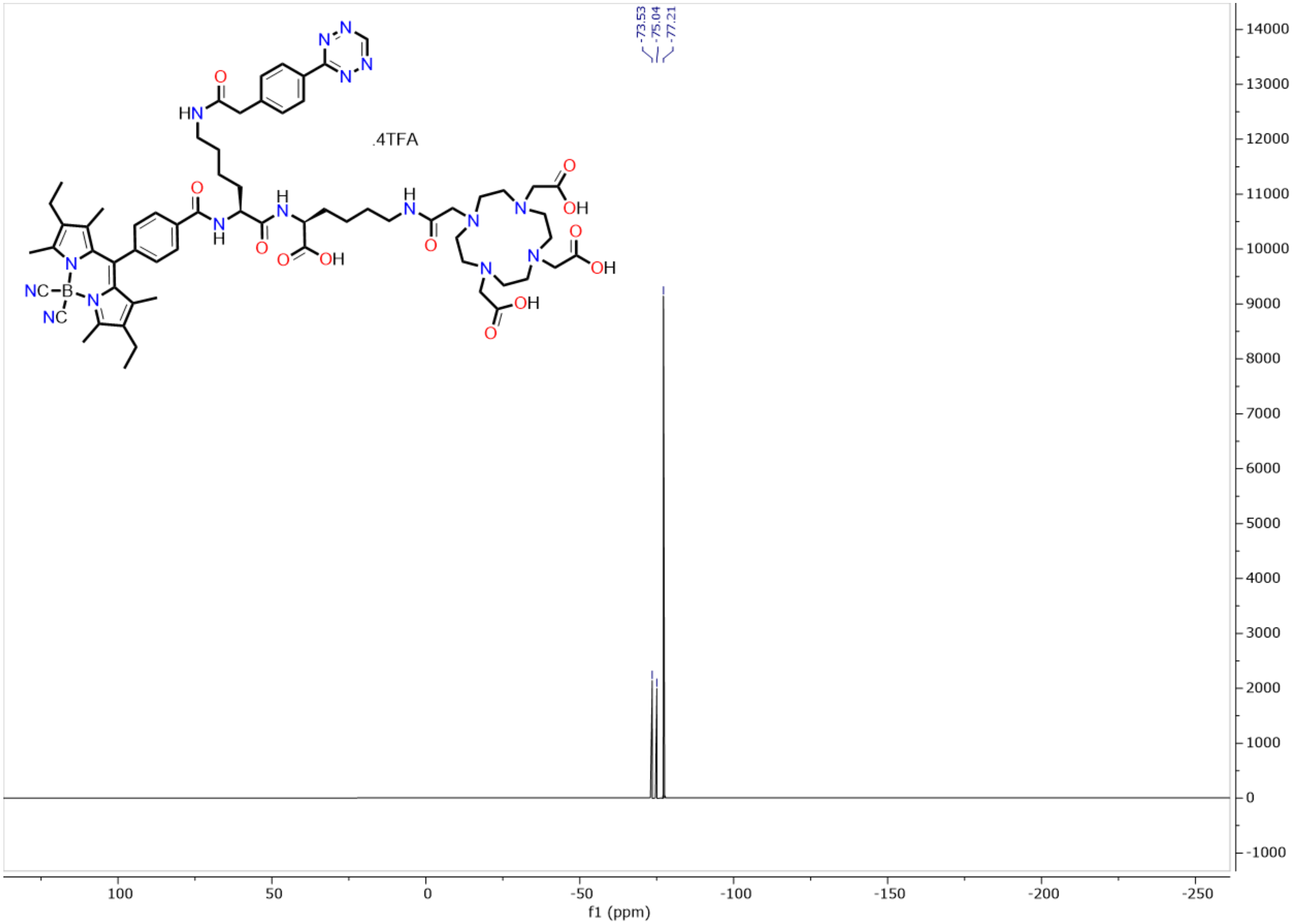

Figure S32. ^{19}F NMR spectrum of **13** (470 MHz, MeOD, 25°C).

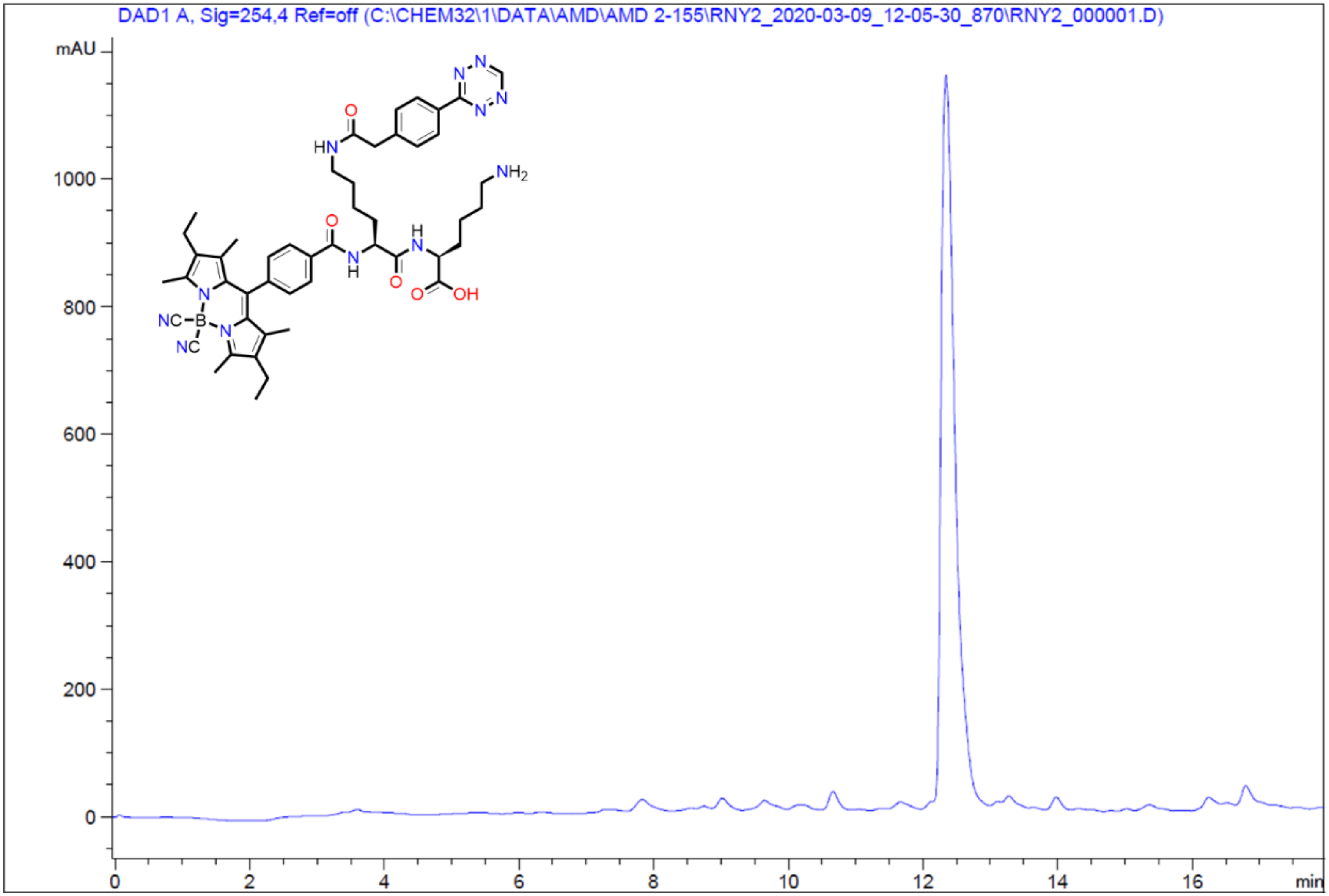

Figure S33. HPLC chromatogram for **12**.

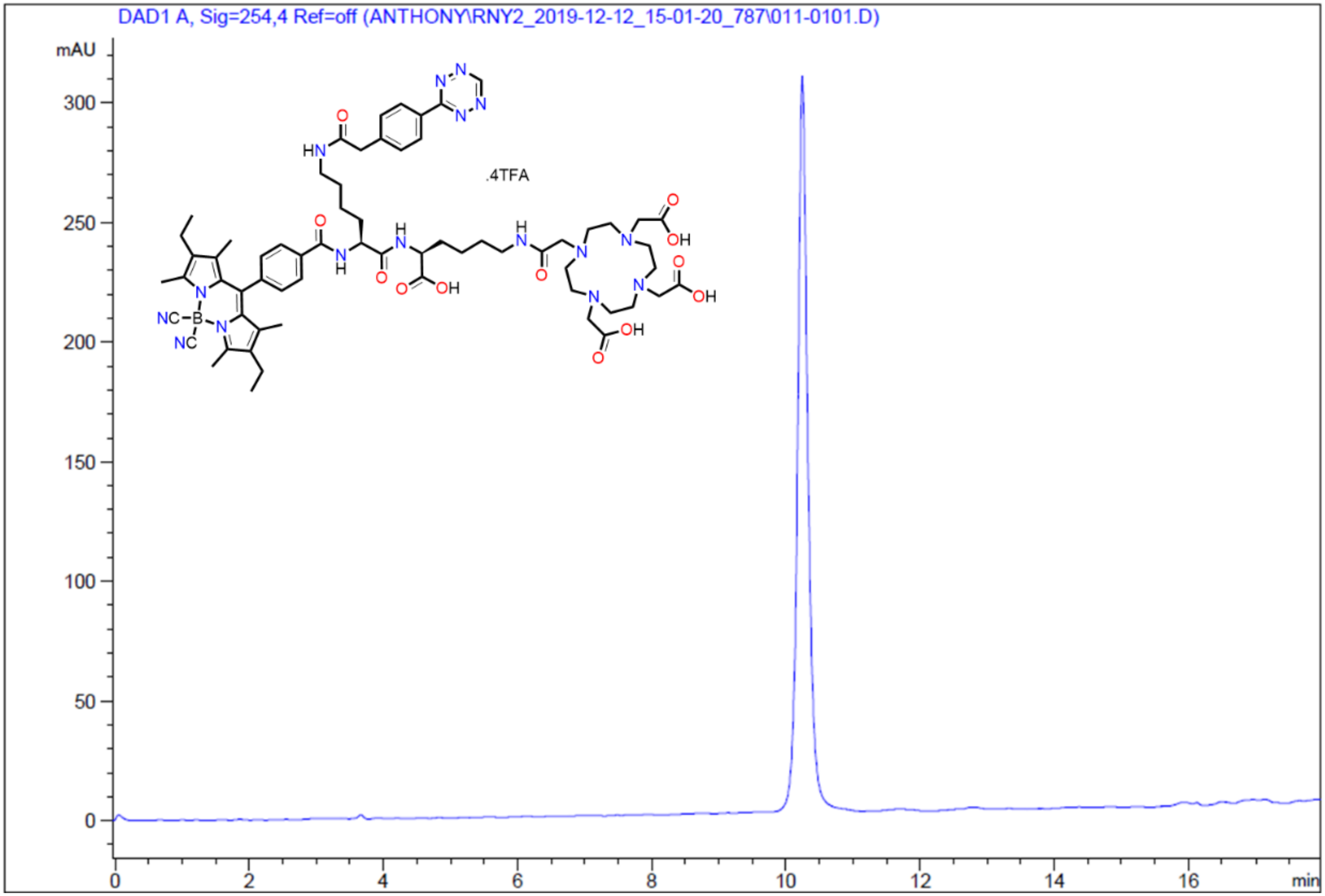

Figure S34. Analytical HPLC UV (254 nm) chromatogram for DO3A-BODIPY-Tz (**13**).

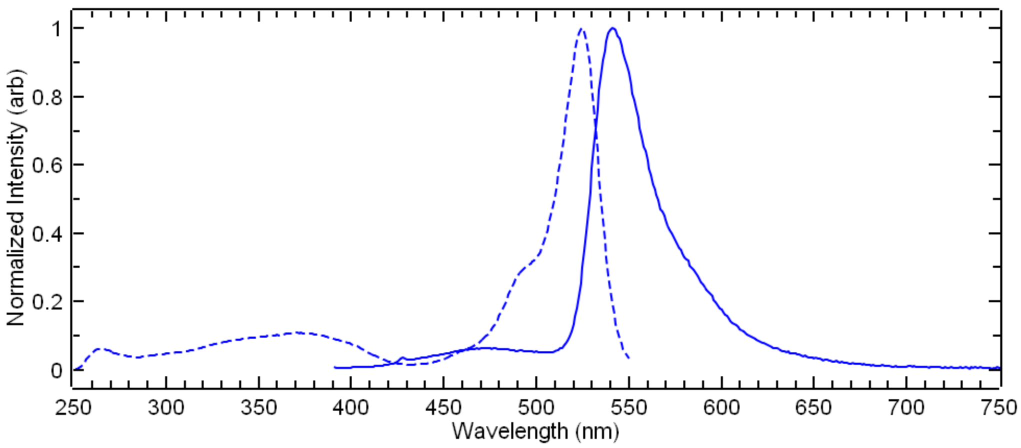

Figure S35. Excitation (dashed) and emission (solid) spectra of **7** in DMSO with Φ_{fl}, (0.87), excitation λ = 527 nm, emission λ= 541 nm.

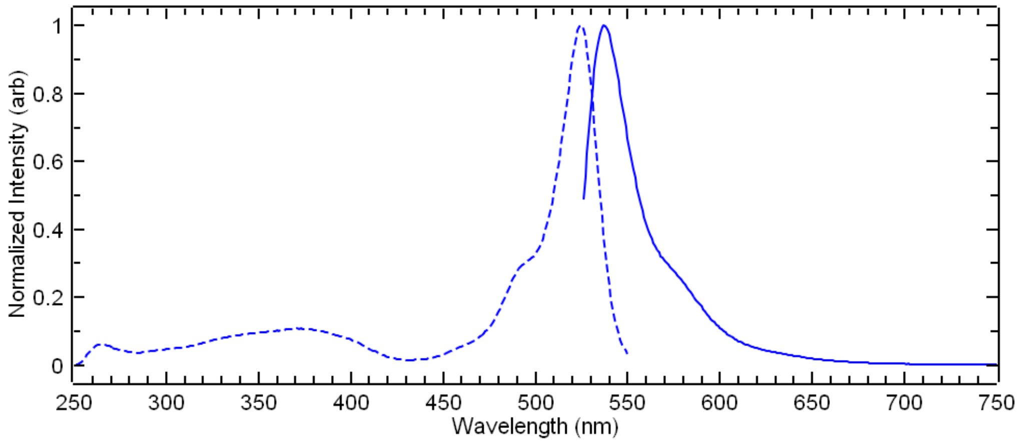

Figure S36. Excitation (dashed) and emission (solid) spectra of **17** in DMSO with Φ_{fl}, (0.79), excitation λ = 525 nm, emission λ= 537 nm

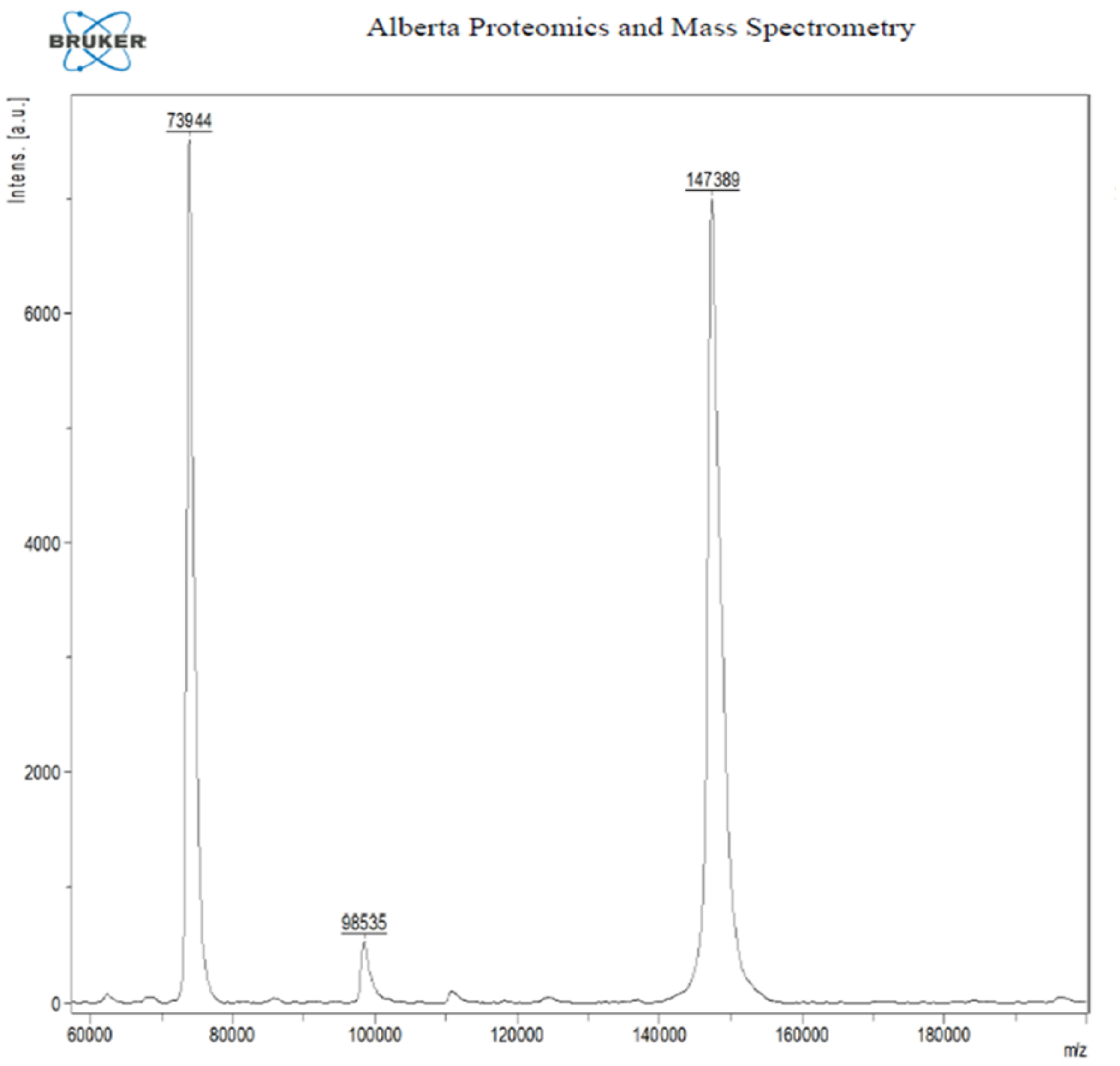

Figure S37. MALDI-TOF MS/MS for trastuzumab.

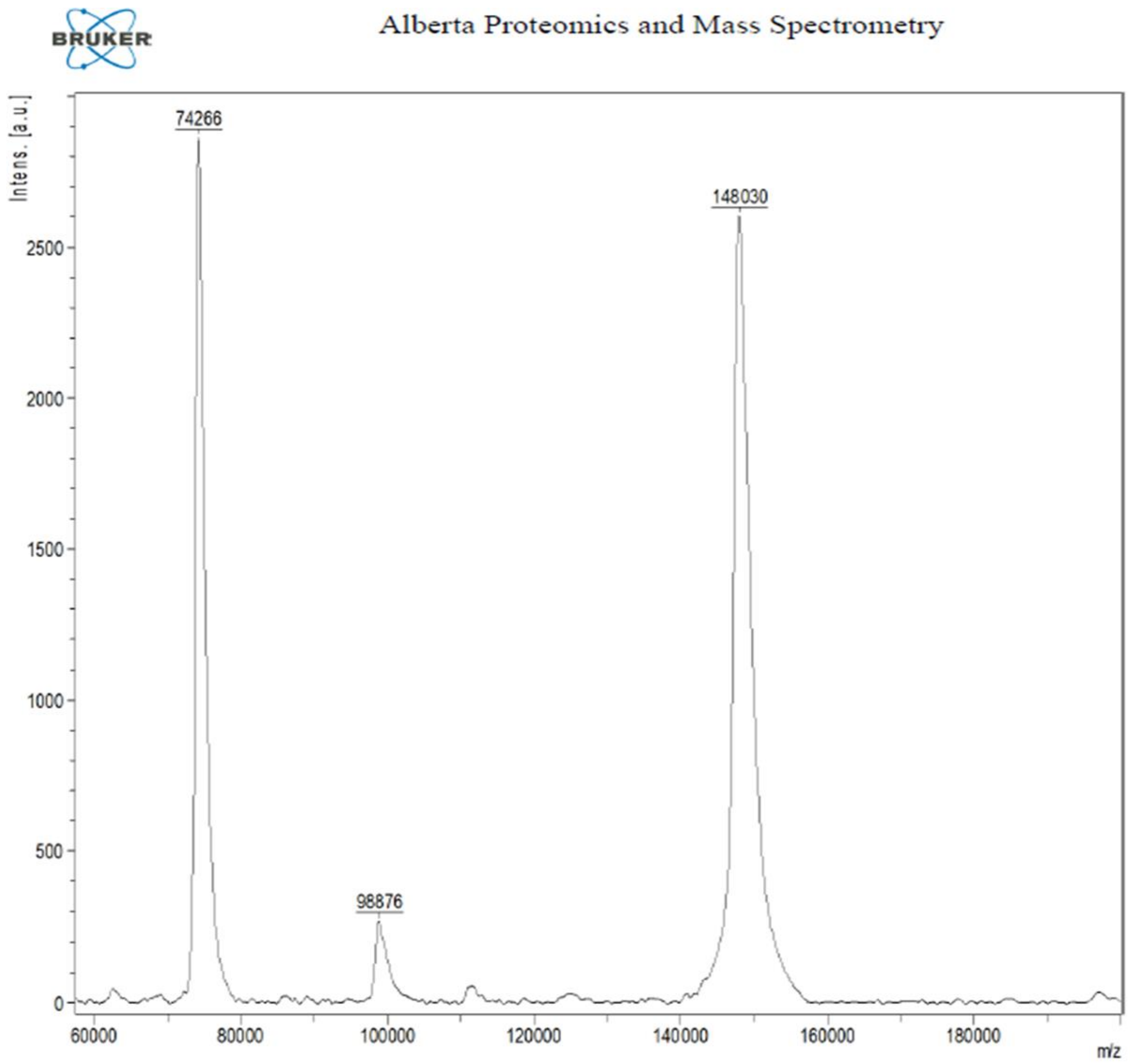

Figure S38. MALDI-TOF MS/MS for TCO-trastuzumab.

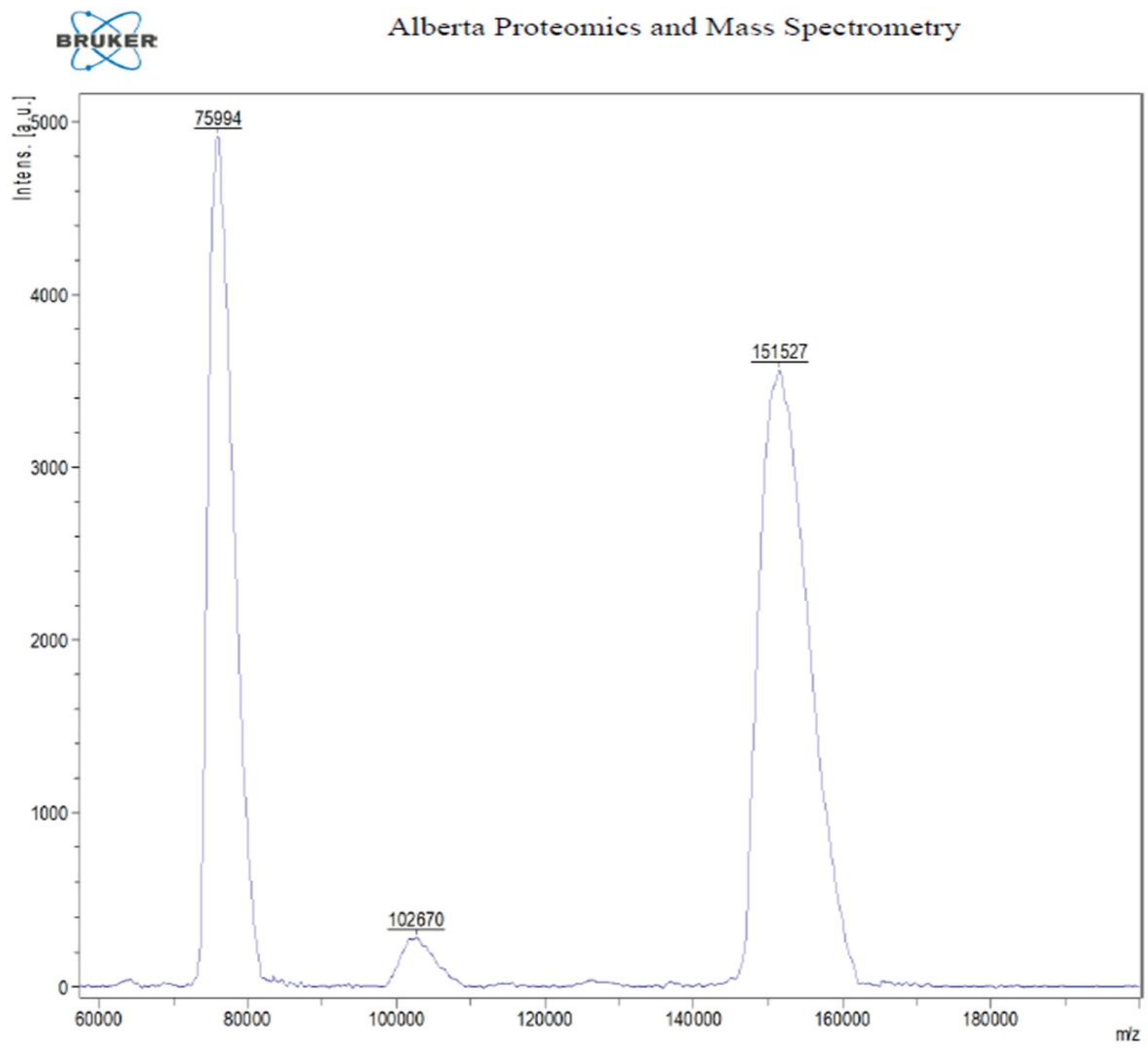

Figure S39. MALDI-TOF MS/MS for DO3A-BODIPY-scaffold-Trastuzumab **19**.

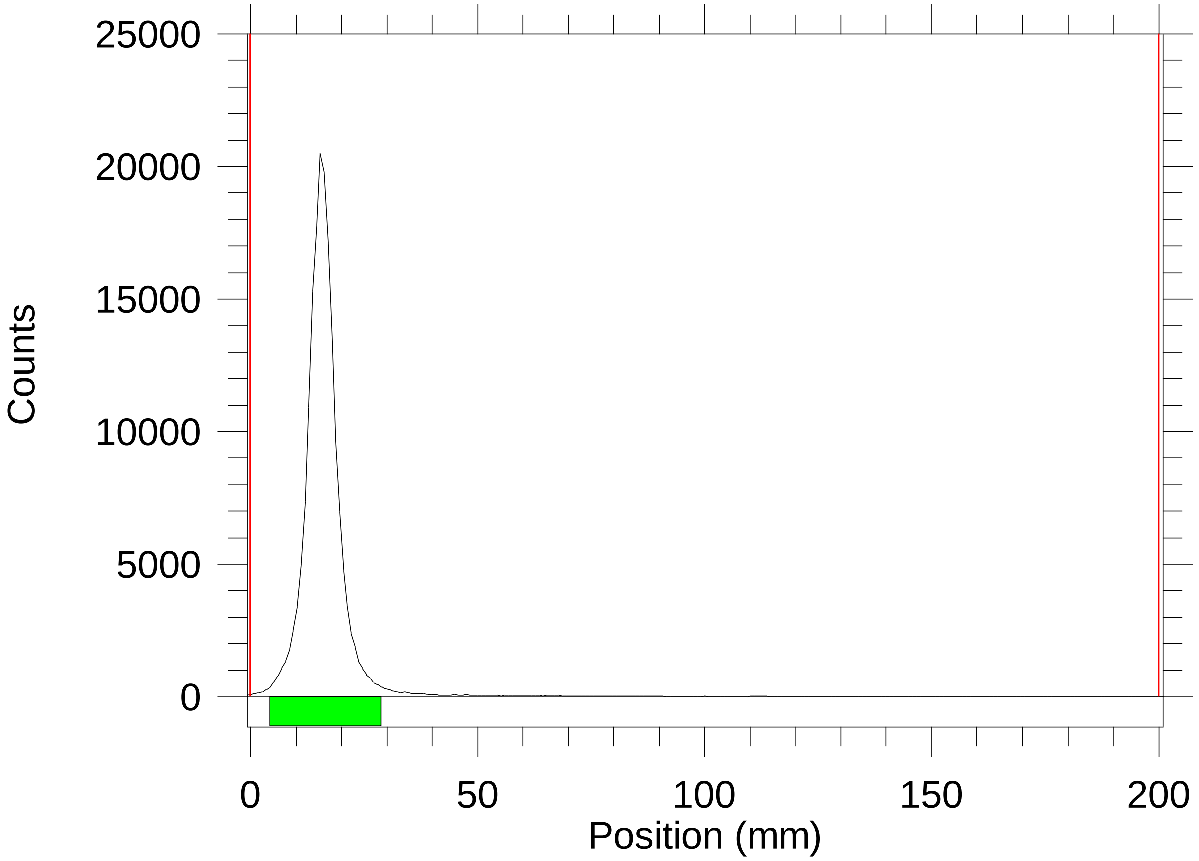

Figure S40. Representative iTLC radiochromatogram of [^{111}In]In-DO3A-BODIPY-Tz developed on iTLC-SG with EDTA (50 mM, pH 5) mobile phase. Radiometal complex remains on baseline (R_f = 0; 20 mm), while unchelated $^{111}In^{3+}$ travels at the solvent front (R_f = 1, ~100 mm).

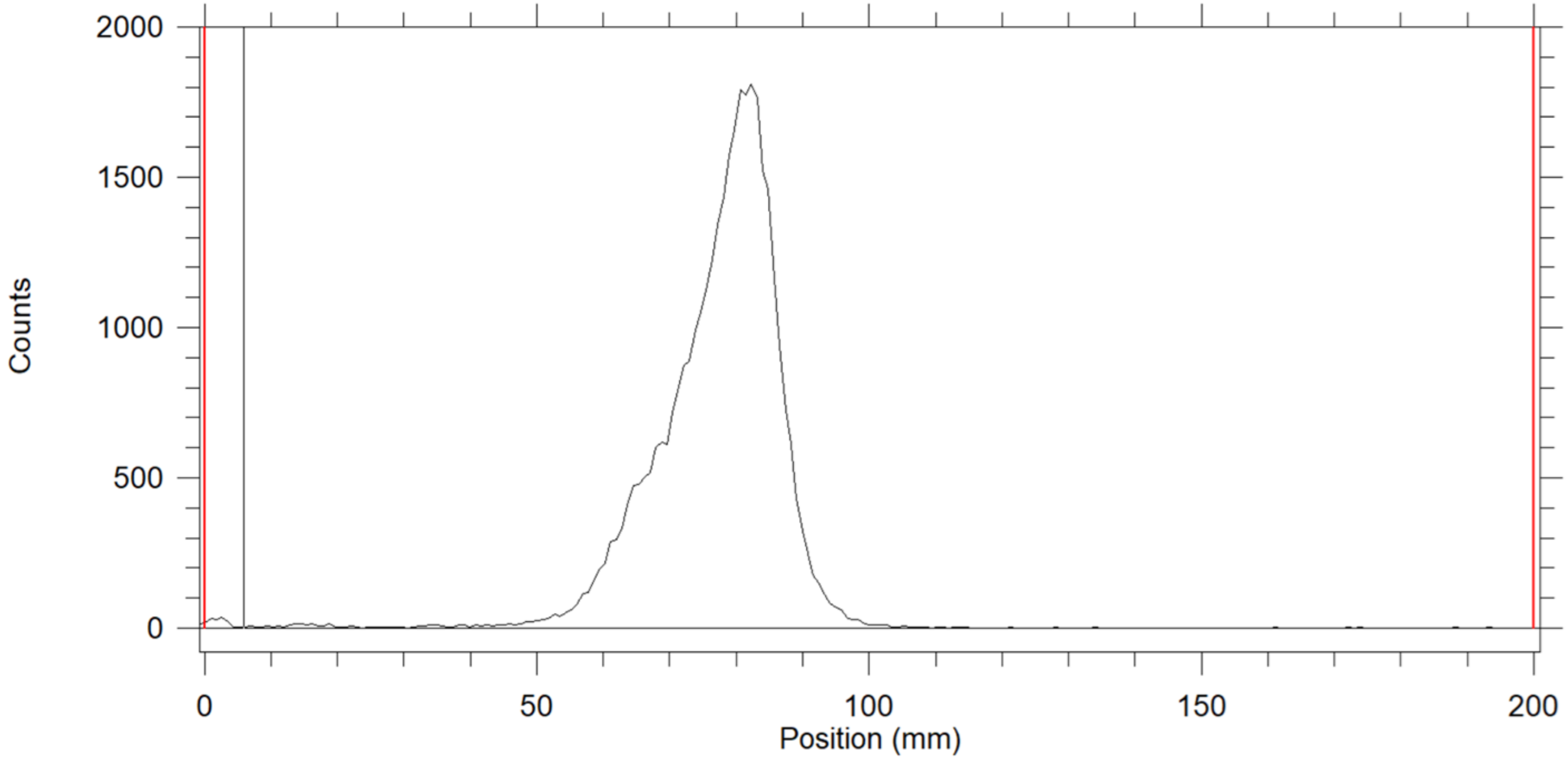

Figure S41. Represents the negative control iTLC radio-chromatogram for ^{225}Ac radiolabeling developed on aluminum-backed TLC silica gel plate and using citric acid (0.4 M, pH 4) mobile phase. Where the free activity travels to the top of the plate (R_f 0.8, 80 mm).

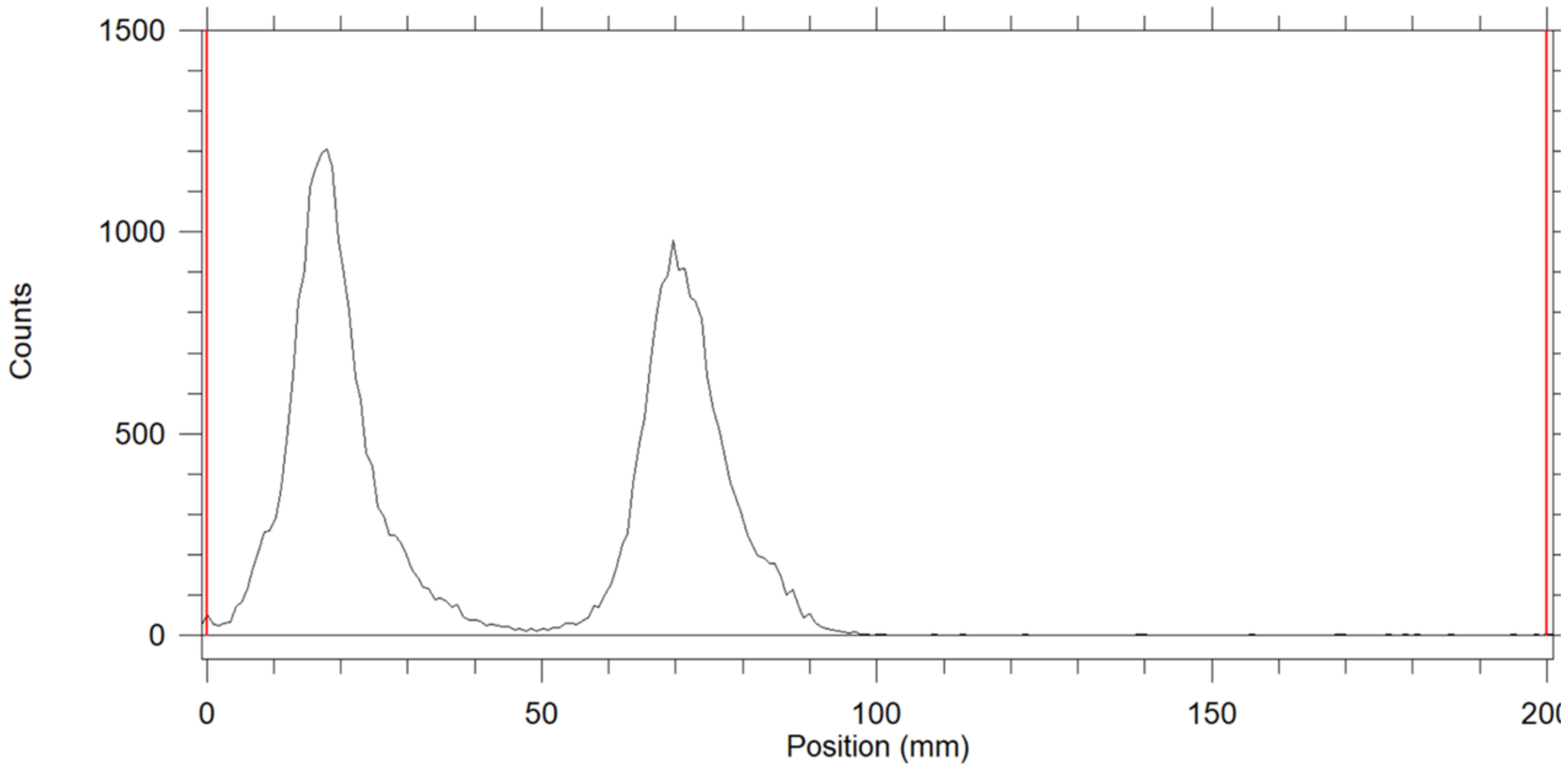

Figure S42. Representative iTLC radiochromatogram of [^{225}Ac]Ac-DO3A-BODIPY-Tz and $^{225}Ac^{3+}$ free activity developed on aluminum-backed TLC silica gel plate and using citric acid (0.4 M, pH 4) mobile phase. Radiometal complex remains on baseline (R_f = 0; 20 mm), while unchelated $^{225}Ac^{3+}$ travels at the solvent front (R_f = 0.75, 75 mm).

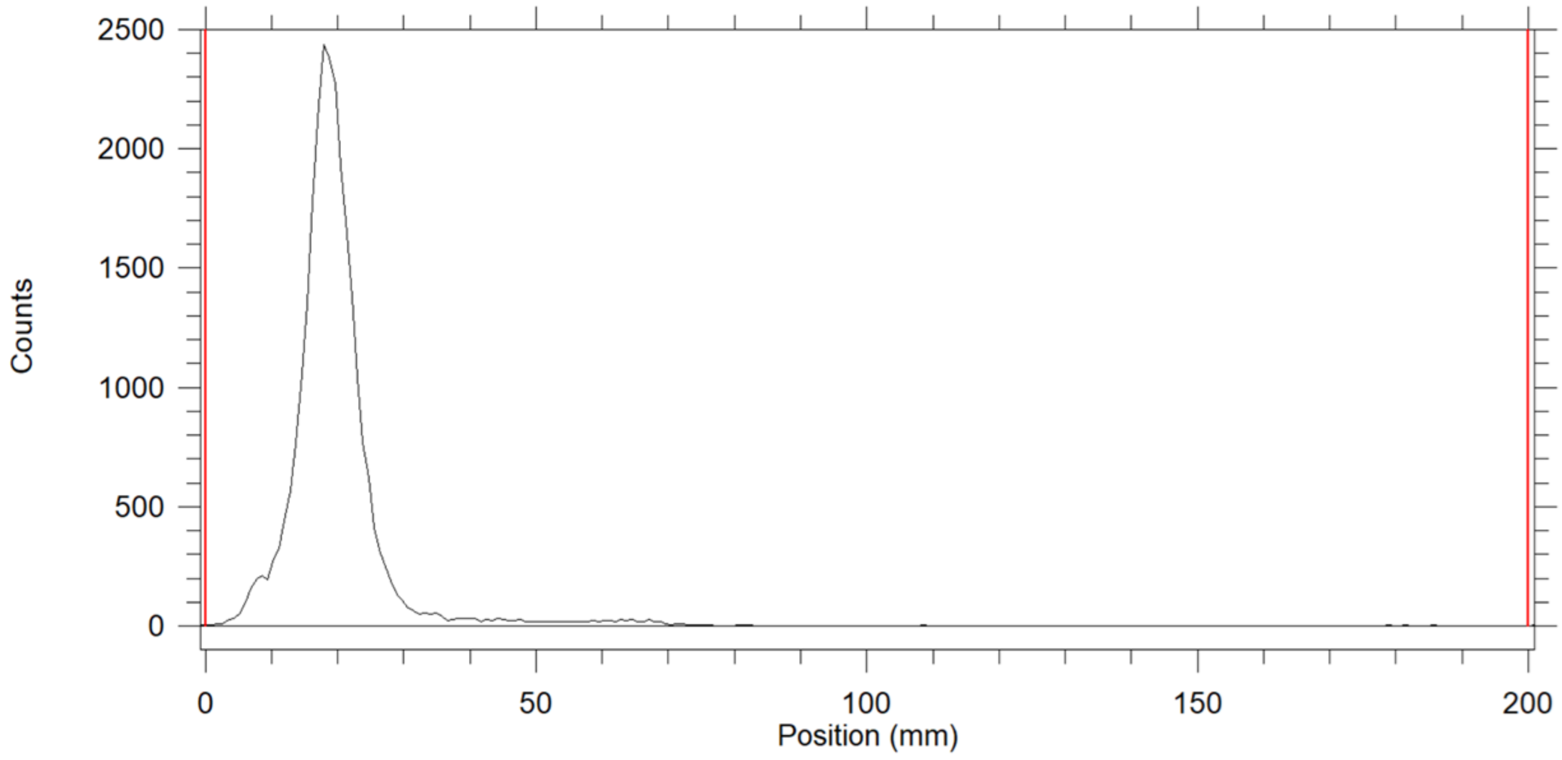

Figure S43. Representative iTLC radiochromatogram of [^{225}Ac]Ac-DO3A-BODIPY-Tz developed on aluminum-backed TLC silica gel plate and using citric acid (0.4 M, pH 4) mobile phase. Radiometal complex remains on baseline (R_f = 0; 20 mm).

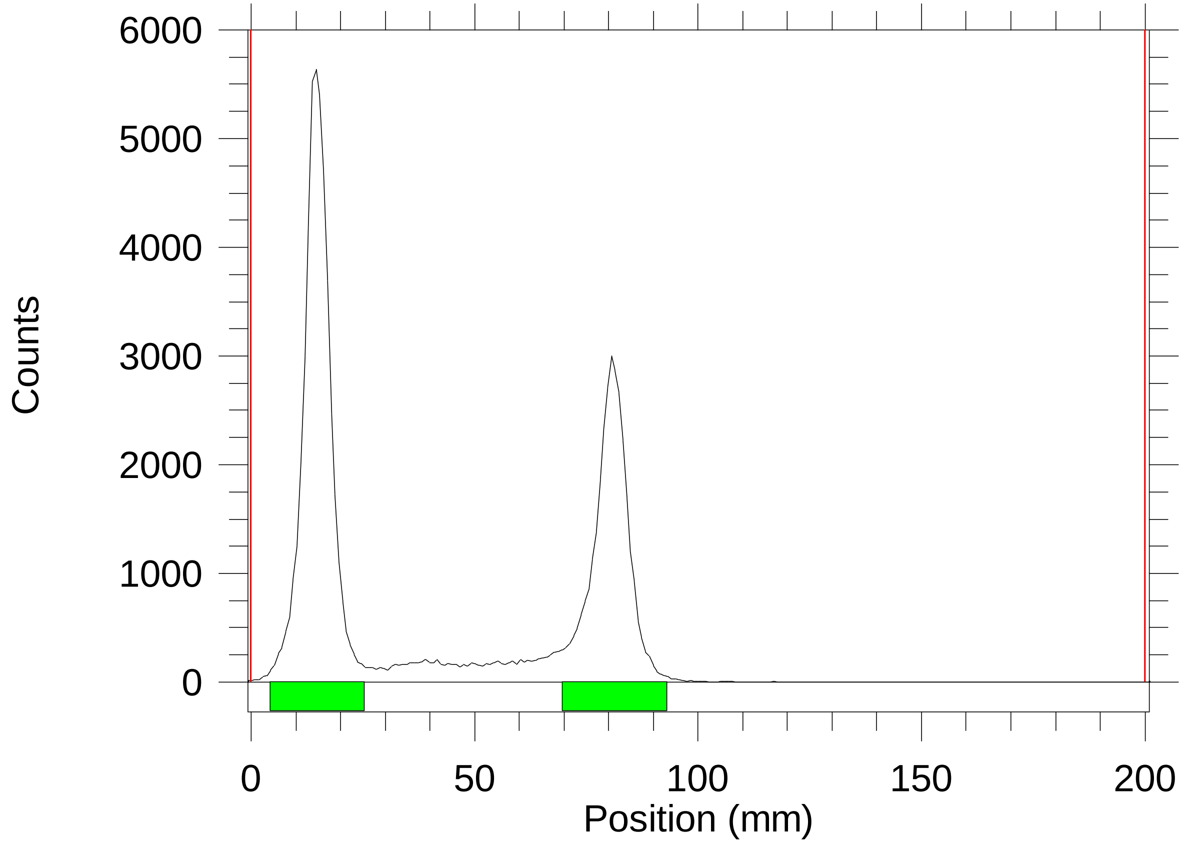

Figure S44. Representative iTLC radiochromatogram of in vitro "click" reaction between [^{111}In]In-DO3A-BODIPY-Tz or [^{225}Ac]Ac-DO3A-BODIPY-Tz and trastuzumab-TCO before PD-10 purification. Developed on iTLC-SG with EtOH/water (50:50). "Clicked" bionconjugate [^{111}In]In-DO3A-BODIPY-trastuzumab and [^{225}Ac]Ac -DO3A-BODIPY-trastuzumab remains at the baseline (R_f = 0, 20 mm) while [^{111}In]In-DO3A-BODIPY-Tz and [^{225}Ac]Ac-DO3A-BODIPY-Tz travels up the plate (R_f ~ 0.8, ~80 mm).

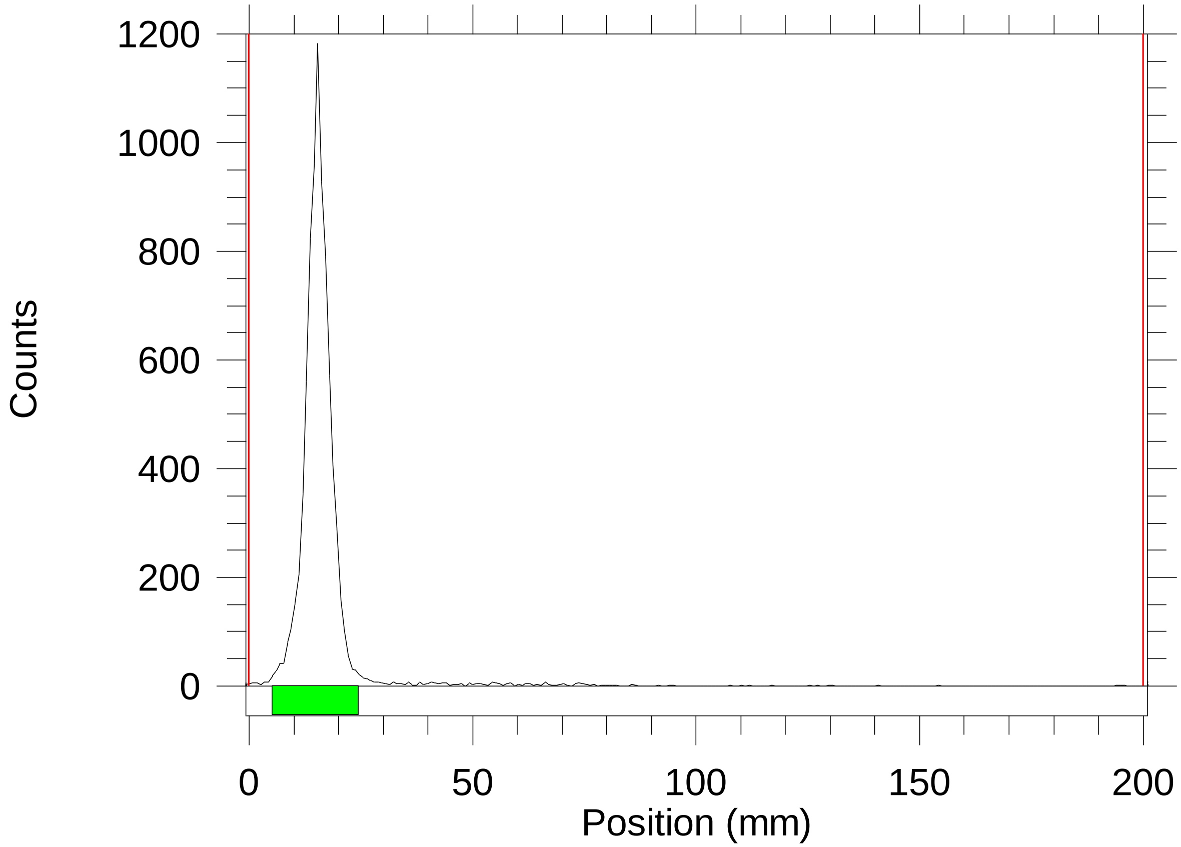

Figure S45. Representative iTLC radiochromatogram of in vitro "click" reaction between [^{111}In]In-DO3A-BODIPY-Tz or [^{225}Ac]Ac-DO3A-BODIPY-Tz and trastuzumab-TCO after PD-10 purification showing RCP > 99%. Developed on iTLC-SG with EtOH/water (50:50). "Clicked" bionconjugate [^{111}In]In-DO3A-BODIPY-trastuzumab and [^{225}Ac]Ac -DO3A-BODIPY-trastuzumab remains at the baseline (R_f = 0, 20 mm) while [^{111}In]In-DO3A-BODIPY-Tz and [^{225}Ac]Ac-DO3A-BODIPY-Tz travels up the plate (R_f ~ 0.8, ~80 mm).

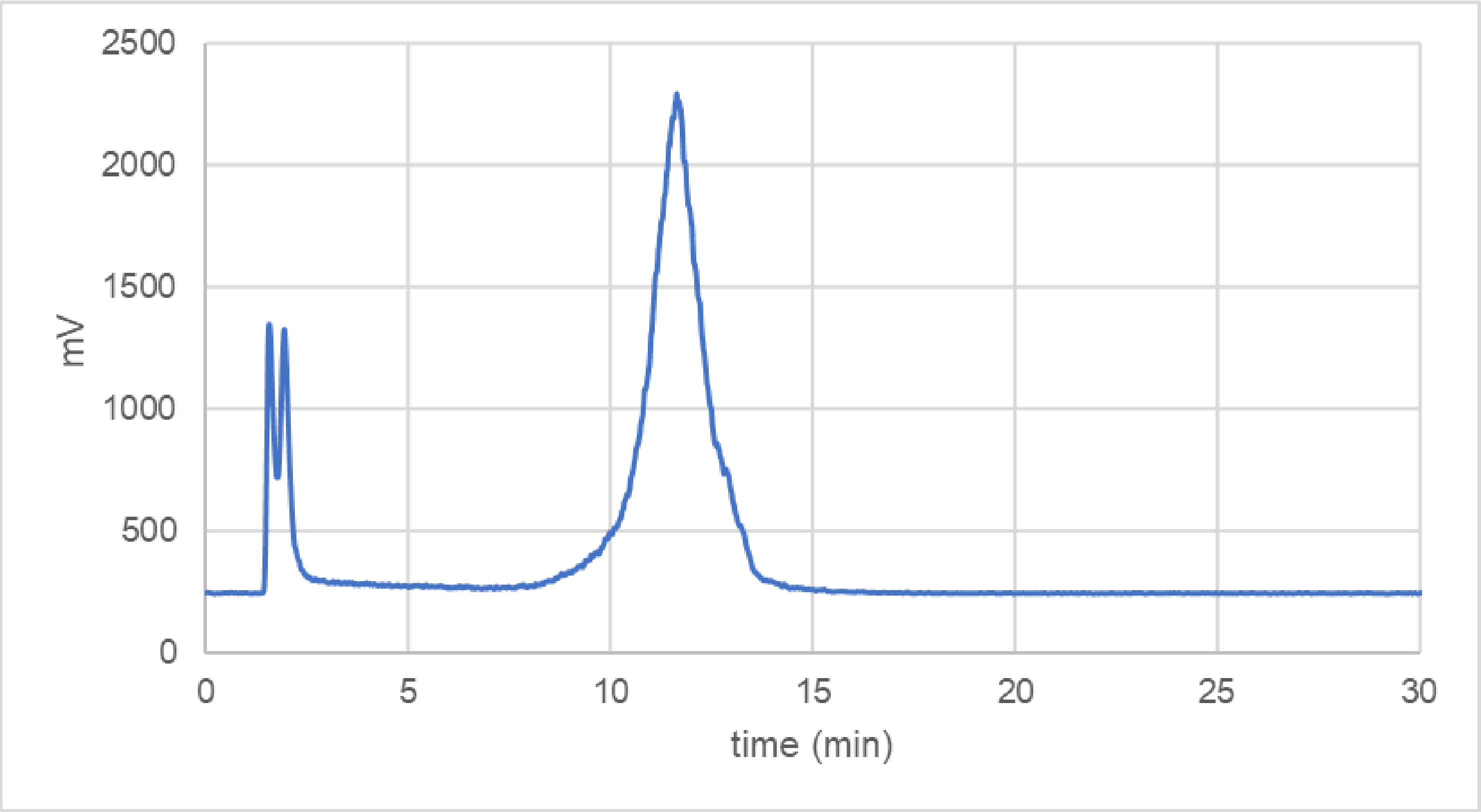

Figure S46. Representative RP-HPLC radiochromatogram of [^{111}In]In-DO3A-BODIPY-Tz. [^{111}In]In-DO3A-BODIPY-Tz: t_R = 11.6 min, uncomplexed $^{111}In^{3+}$: t_R = 1.6 min.

Table S1. Biodistribution 6 days after injection of [^{111}In]In-DO3A-BODIPY-Tz-TCO-trastuzumab in SKOV-3 (HER2+) tumors bearing nude mice. Values are mean ± SD (*n* = 4 mice) after injection of 26.3 ± 0.3 µg, 4.1 ± 0.1 MBq.

Organs	%ID/g (Day 6)
**Blood**	4.15 ± 0.92
**Fat**	0.47 ± 0.16
**Uterus**	4.94 ± 3.37
**Ovaries**	17.20 ± 11.24
**Intestine**	0.73 ± 0.16
**Spleen**	6.16 ± 1.35
**Liver**	8.97 ± 2.01
**Pancreas**	0.68 ± 0.07
**Stomach**	0.85 ± 0.21
**Adrenal glands**	1.77 ± 0.39
**Kidney**	3.46 ± 0.44
**Lungs**	2.33 ± 0.46
**Heart**	1.28 ± 0.12
**SKOV-3 tumor**	21.24 ± 5.56
**Muscle**	0.46 ± 0.11
**Bone**	1.33 ± 0.31
**Brain**	0.12 ± 0.01
